# Gene expression and metabolite levels converge in the thermogenic spadix of skunk cabbage

**DOI:** 10.1093/plphys/kiae059

**Published:** 2024-02-06

**Authors:** Haruka Tanimoto, Yui Umekawa, Hideyuki Takahashi, Kota Goto, Kikukatsu Ito

**Affiliations:** United Graduate School of Agricultural Science, Iwate University, Morioka, Iwate 020-8550, Japan; Department of Planning and General Affairs, Akita Research Institute of Food and Brewing, Araya-machi, Akita 010-1623, Japan; Department of Agriculture, School of Agriculture, Tokai University, Kumamoto 862-8652, Japan; Faculty of Agriculture, Iwate University, Morioka, Iwate 020-8550, Japan; United Graduate School of Agricultural Science, Iwate University, Morioka, Iwate 020-8550, Japan; Faculty of Agriculture, Iwate University, Morioka, Iwate 020-8550, Japan

## Abstract

The inflorescence (spadix) of skunk cabbage (*Symplocarpus renifolius*) is strongly thermogenic and can regulate its temperature at around 23 °C even when the ambient temperature drops below freezing. To elucidate the mechanisms underlying developmentally controlled thermogenesis and thermoregulation in skunk cabbage, we conducted a comprehensive transcriptome and metabolome analysis across 3 developmental stages of spadix development. Our RNA-seq analysis revealed distinct groups of expressed genes, with *selenium-binding protein 1/methanethiol oxidase* (*SBP1/MTO*) exhibiting the highest levels in thermogenic florets. Notably, the expression of *alternative oxidase* (*AOX*) was consistently high from the prethermogenic stage through the thermogenic stage in the florets. Metabolome analysis showed that alterations in nucleotide levels correspond with the developmentally controlled and tissue-specific thermogenesis of skunk cabbage, evident by a substantial increase in AMP levels in thermogenic florets. Our study also reveals that hydrogen sulfide, a product of SBP1/MTO, inhibits cytochrome *c* oxidase (COX)-mediated mitochondrial respiration, while AOX-mediated respiration remains relatively unaffected. Specifically, at lower temperatures, the inhibitory effect of hydrogen sulfide on COX-mediated respiration increases, promoting a shift toward the dominance of AOX-mediated respiration. Finally, despite the differential regulation of genes and metabolites throughout spadix development, we observed a convergence of gene expression and metabolite accumulation patterns during thermogenesis. This synchrony may play a key role in developmentally regulated thermogenesis. Moreover, such convergence during the thermogenic stage in the spadix may provide a solid molecular basis for thermoregulation in skunk cabbage.

## Teaser

Comparative transcriptomic and metabolomic analyses unveil the crucial role of gene and metabolite expression convergence in the developmentally regulated thermogenesis of *Symplocarpus renifolius*.

## Introduction

Thermogenesis, an endogenous metabolic heat-production process in specific organs or tissues, is a physiological phenomenon observed across various angiosperm species. Plant thermogenesis is often associated with the release of an odor or scent from the thermogenic organ. Notably, this mechanism attracts pollinators during the reproductive stages and concurrently creates a warm habitat advantageous for energy conservation in visiting insects (Meeuse and Raskin 1988; Seymour et al. 2003; Etl et al. 2022). A number of plants, including the titan arum (*Amorphophallus titanum*), dead-horse arum (*Helicodiceros muscivorus*), Crete arum (*Arum concinnatum*), and lords-and-ladies (*Arum maculatum*) from the Araceae family and the sacred lotus (*Nelumbo nucifera*) and American lotus (*Nelumbo lutea*) from the lotus family, exhibit thermogenesis, although the extent of their temperature increase and the timing of this phenomenon vary among species (Wagner et al. 2008; Barthlott et al. 2009; Kakizaki et al. 2010; Dieringer et al. 2014; Zheng et al. 2022).

*S*kunk cabbage (*Symplocarpus renifolius*) is a thermogenic species that flowers in late winter or early spring, a period when ambient temperatures often drop below freezing (Ito et al. 2004; Ito and Ito 2005; Onda et al. 2008). The inflorescence, known as the spadix, is capable of increasing its internal temperature in a cold environment (Onda et al. 2008). A recent phylogeographic study in the genus *Symplocarpus* suggests that such thermogenesis contributed to the expansion of the distribution of *S. renifolius* during the Ice Age (Sato et al. 2023).

In *S. renifolius*, the spadix also exhibits a unique phenomenon known as “thermoregulation”, maintaining a relatively stable internal temperature of approximately 23 °C, regardless of external air temperature fluctuations. The distinction between thermogenesis and thermoregulation can be further elucidated through thermal clamping of the flowers of thermogenic plants (Seymour et al. 2010). In thermoregulatory *S. renifolius*, a decrease in tissue temperature leads to an increase in respiration rate, a response not generally observed in flowers exhibiting only thermogenesis. The spadices of *S. renifolius* exhibit their maximum respiration rate at 15 °C, defining the lower limit of their thermoregulatory range that spans from 15 to 30 °C. Our previous study demonstrated that thermoregulation in *S. renifolius* is mediated through a biochemical equilibrium, characterized by an overall activation energy displaying a negative value within its thermoregulatory temperature range (Umekawa et al. 2016).

The spadix of *S. renifolius* is composed of florets and pith. Our previous thermoscopic analyses of the thermogenic stage of spadices revealed that the florets, located on the outer parts of the spadix, maintain higher temperatures compared to the inner pith tissue (Onda et al. 2008). Thus, it has been suggested that the thermogenesis predominantly occurs in the florets, while the pith does not directly contribute to this process in the spadix (Onda et al. 2008).

The development of the spadix, intimately linked to its thermogenic function, progresses through 3 distinct stages: the prethermogenic, female, and male stages (Uemura et al. 1993; Ito-Inaba et al. 2009). During the prethermogenic stage, which corresponds to the early developmental phase of the spadix, the organ structure is still immature and devoid of visible stigmas. This stage sets the groundwork for subsequent physiological changes and does not involve any thermogenic activity. Subsequently, the spadix transitions into the female stage, characterized by a substantial increase in thermogenesis and the commencement of thermoregulation. At this stage, the florets have reached maturity, accompanied by a pistil and a still immature stamen, as well as a pith containing vascular bundles. The spadix then progresses to the male stage, also referred to as the postthermogenic stage via the bi-sexual stage, a transient period interlinking the female and male stages. During the male stage, the stigmas vanish, pollen appears on the surface of the spadix, and thermogenesis considerably diminishes (Uemura et al. 1993). This developmental pattern found in the spadix of *S. renifolius* is an example of dichogamy, where the same plant undergoes different sex phases at different times within a single flowering period (Uemura et al. 1993; Patt et al. 1995; Albre et al. 2003; Jabbour et al. 2022).

Despite exhibiting dichogamy, being self-incompatible, and requiring pollen from other individuals for reproduction (Uemura et al. 1993), *S. renifolius* is less reliant on thermogenesis for pollinator attraction compared to other thermogenic plants. Interestingly, despite its common name “skunk cabbage”, which is shared with its North American relative *Symplocarpus foetidus*, neither of these species emit the strong, unpleasant odor typically associated with skunks during thermogenesis (Knutson 1979; Ito-Inaba 2014). This observation was corroborated by previous findings (Oguri et al. 2019), which reported that no substantial changes were observed in the compositions of floral volatiles, including odor compounds such as dimethyl sulfide (DMS), dimethyl disulfide (DMDS), and dimethyl trisulfide (DMTS) throughout the 3 developmental stages of the spadices of *S. renifolius*, including the thermogenic stage. Furthermore, there is little evidence of insects being attracted to the emitted compounds from *S. renifolius* spadices in freezing cold environments, unlike the case of *Calliphoridae* (blow flies) and the dead-horse arum (Stensmyr et al. 2002). However, physiological studies have demonstrated the crucial role of a regulated temperature (23 °C) in pollen germination and pollen tube elongation in *S. renifolius* (Seymour et al. 2009).

The mechanisms of cellular thermogenesis in animals, known as nonshivering thermogenesis, have been extensively investigated in brown adipose tissues (BAT), with mitochondrial respiration recognized as a major driver of cellular heat-production (Enerbäck et al. 1997; Cheng et al. 2021; Brownstein et al. 2022; Takeda et al. 2023). Thermogenesis can be augmented through several biochemical stratagems designed to maximize the thermal effect resulting from substrate oxidation (Chouchani et al. 2019). These include (i) elevating the rate of ATP turnover to stimulate cellular bioenergetics; (ii) allowing electron transfer chains to proceed independent of concurrent proton translocation across the inner membrane of the mitochondria, a phenomenon referred to as electron slip or leak; (iii) enabling the dissipation of the proton electrochemical gradient across the inner mitochondrial membrane in the absence of coupled ATP synthesis, a process known as proton leak; and (iv) inducing ATP hydrolysis without concomitant cellular work, a mechanism known as a futile cycle. In mammalian BAT cells, the primary thermogenic effector is the uncoupling protein 1 (UCP1), a key player in facilitating proton leak dynamics (Enerbäck et al. 1997; Golozoubova et al. 2001; Divakaruni and Brand 2011; Chang et al. 2019).

Plant thermogenesis is also impacted by mitochondrial respiration activities, including mitochondrial density (Ito-Inaba et al. 2009), and biochemical variables that decrease ATP synthesis efficiency. This predominantly involves the establishment of a proton gradient in the mitochondrial electron transport chain. A critical component in this process is the mitochondrial alternative oxidase (AOX), which enables the direct transfer of electrons from ubiquinol to oxygen, bypassing the direct proton transport (Moore et al. 2013; Selinski et al. 2018; El-Khoury et al. 2022). This mechanism aligns with the category of electron slip or leak. Similarly, rotenone-insensitive NAD(P)H dehydrogenases, internal alternative NAD(P)H:ubiquinone oxidoreductase (NDA) and external alternative NAD(P)H:ubiquinone oxidoreductase (NDB), located on both sides of the plant mitochondrial inner membrane, function as bypasses for complex I without relying on proton pumping (Kakizaki et al. 2012; Schertl and Braun 2014).

In *S. renifolius*, *AOX* transcripts and AOX proteins are remarkably abundant in the thermogenic spadix (Onda et al. 2007). Moreover, *AOX* transcripts are specifically found in the florets, particularly the petal and pistil (Sayed et al. 2016). Given the tissue-specific expression of *AOX* in the petal and pistil, it is probable that these tissues in the florets play an important role in developmentally controlled thermogenesis in *S. renifolius*, while the pith does not directly contribute to this process.

Plant thermogenesis is a complex cellular process, likely requiring the coordinated expression of multiple genes and associated metabolic changes, both developmentally and temporally. Omics-based approaches, such as next-generation sequencing, have offered valuable insights into the gene regulation and metabolic pathways associated with plant thermogenesis (Ito-Inaba et al. 2012; Ito et al. 2013; Onda et al. 2015; Zou et al. 2020; Szenteczki et al. 2022; Wang et al. 2022; Zheng et al. 2022). For instance, transcriptome analyses have identified secondary metabolic pathways associated with the genes induced during thermogenesis in *A. concinnatum* (Onda et al. 2015) and lotus (Zheng et al. 2022). Moreover, metabolomic analysis with thermogenic *Dracunculus vulgaris* has revealed temperature-sensitive metabolic changes within the thermogenic organ of this plant (Ito et al. 2013). Notably, an early effort to understand the gene expression changes during thermogenesis in *S. renifolius* has been carried out using the SuperSAGE technology (Ito-Inaba et al. 2012). In the study by Ito-Inaba et al. (2012), an initial glimpse into the gene expression landscape in thermogenic and postthermogenic spadices was provided, yet it failed to fully capture the transcriptomic complexity. This limitation was, in part, due to the inherent restrictions of the SuperSAGE technology. Most crucially, in the study by Ito-Inaba et al. (2012), key genes involved in thermogenesis, such as the gene encoding AOX, which is a central component of plant thermogenic processes, were overlooked.

Despite the recent advancements in omics analysis for thermogenic plants, as mentioned earlier, no single study to date has undertaken a comprehensive combination of transcriptomic and metabolomic analyses on the same tissue in any thermogenic plant. This has left an important gap in our understanding of the dynamic interplay between gene expression and metabolite accumulation, particularly during the development of thermogenesis in the spadix of *S. renifolius*. Our present study was designed to bridge this critical research gap by focusing on *S. renifolius*, a species notable for its robust thermoregulation in cold environments. Through comparative analysis of florets and pith tissues across various thermogenic stages of the spadices, we provide a detailed profile of gene expression and metabolic pathways throughout the developmental stages of *S. renifolius*. Furthermore, we discuss metabolic evidence that sheds light on the absence of odor emission from the thermogenic spadices of *S. renifolius*. Finally, we propose a convergence model that could potentially deepen our understanding of the mechanisms underlying thermogenesis and thermoregulation in this plant.

## Results

### Thermogenesis of *S. renifolius*

**Figure 1.**
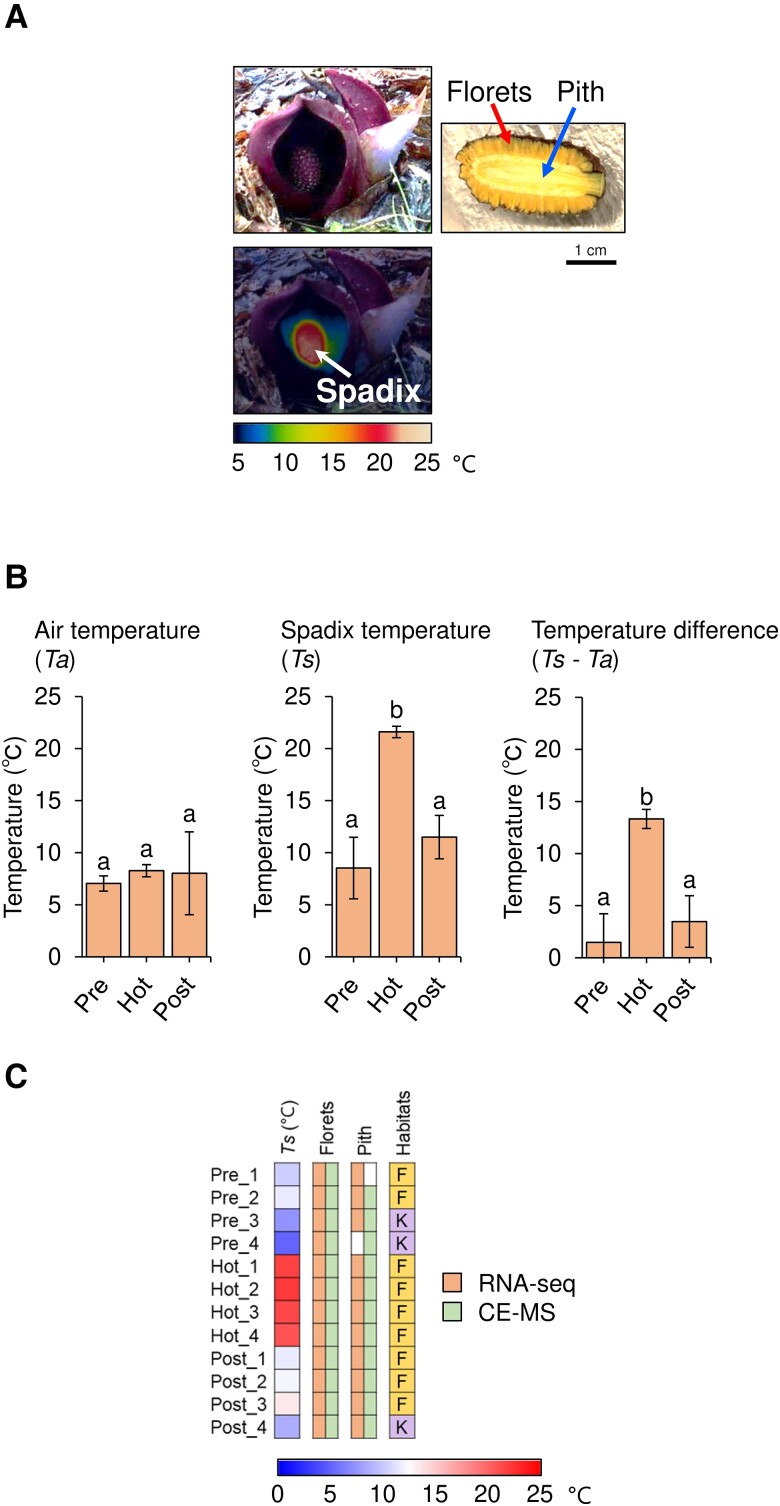
Thermogenesis of *S. renifolius*. **A)** Images showing the thermogenic stage of intact *S. renifolius* (upper left), a thermal image capturing the surface temperature (lower left), and a longitudinal section indicating the positions of florets and pith (right). **B)** Comparative data for air temperature (*Ta*), spadix temperature (*Ts*), and their difference (*Ts−Ta*). The stages of thermogenesis are denoted as “Pre” (prethermogenic), “Hot” (thermogenic), and “Post” (postthermogenic). The mean values and standard deviations from 4 independent measurements are presented in bar graphs with error bars, respectively. Statistically significant differences are indicated by different letters above the bars (Tukey–Kramer test: *P* < 0.05). **C)** Details of the analyzed samples, indicated by labels corresponding to the thermogenic stages of the spadix in *S. renifolius*. Stages are labeled as “Pre_x” for prethermogenic, “Hot_x” for thermogenic, and “Post_x” for postthermogenic samples, where “x” denotes the specific sample number. Each label also includes information on the spadix temperature (*Ts*), the sampling site [either Fujine (F) or Kanegasaki (K)], and the analysis method used (RNA-seq or CE-MS). Empty boxes in “Pre_1” and “Pre_4” denote samples that were not subjected to CE-MS or RNA-seq analysis due to insufficient tissue quantity resulting from the small size of the pith tissues at the prethermogenic spadices. CE-MS, capillary electrophoresis-mass spectrometry.

For the transcriptome and metabolome analyses, we utilized the natural habitats of *S. renifolius*. The spadix, comprising both florets and pith, is the critical organ responsible for thermogenesis in this plant (Fig. 1A). In our previous study, we demonstrated that the florets, located in the outer part of the spadix, were the primary tissues for thermogenesis (Onda et al. 2008). Therefore, in the present study, we assume that the inner pith is the less thermogenic tissue in the spadix. In our temperature measurements, we observed that the intensity of thermogenesis varied with the development of the spadix in *S. renifolius*. We classified the thermogenic stages of the spadix into 3 distinct categories based on both its morphology and temperature profile. These stages are denoted as “Pre” (prethermogenic), “Hot” (thermogenic), and “Post” (postthermogenic) (Fig. 1B and C, and Supplementary Table S1). Although the ambient air temperature (denoted as “*Ta*” in Fig. 1B) remained relatively consistent across the different thermogenic stages during the sampling period, the temperature within the spadix (denoted as “*Ts*” in Fig. 1B and C) varied in accordance with developmental stages. Consequently, both “*Ts*” and the temperature differential “*Ts*−*Ta*” served as reliable indicators of stage-specific thermogenesis in our present study (Fig. 1B). During the thermogenic stage, the spadix temperature peaked at approximately 23 °C, indicating a substantial temperature differential of nearly 15 °C compared to the surrounding air temperature (Fig. 1B).

### Global gene expression profile

Our RNA-seq analysis led to the successful identification of 118,676 transcripts from our de novo assembly (Supplementary Figs. S1 and S2A). Among these, 63,705 transcripts were longer than 1,000 base pairs, establishing a solid foundation for further investigation. Subsequent analysis was then focused on a specific subset of 15,904 sequences, selected for their homology with known amino acid sequences in Arabidopsis (*Arabidopsis thaliana*) (Supplementary Data Set 1 and Supplementary Fig. S2A).

**Figure 2.**
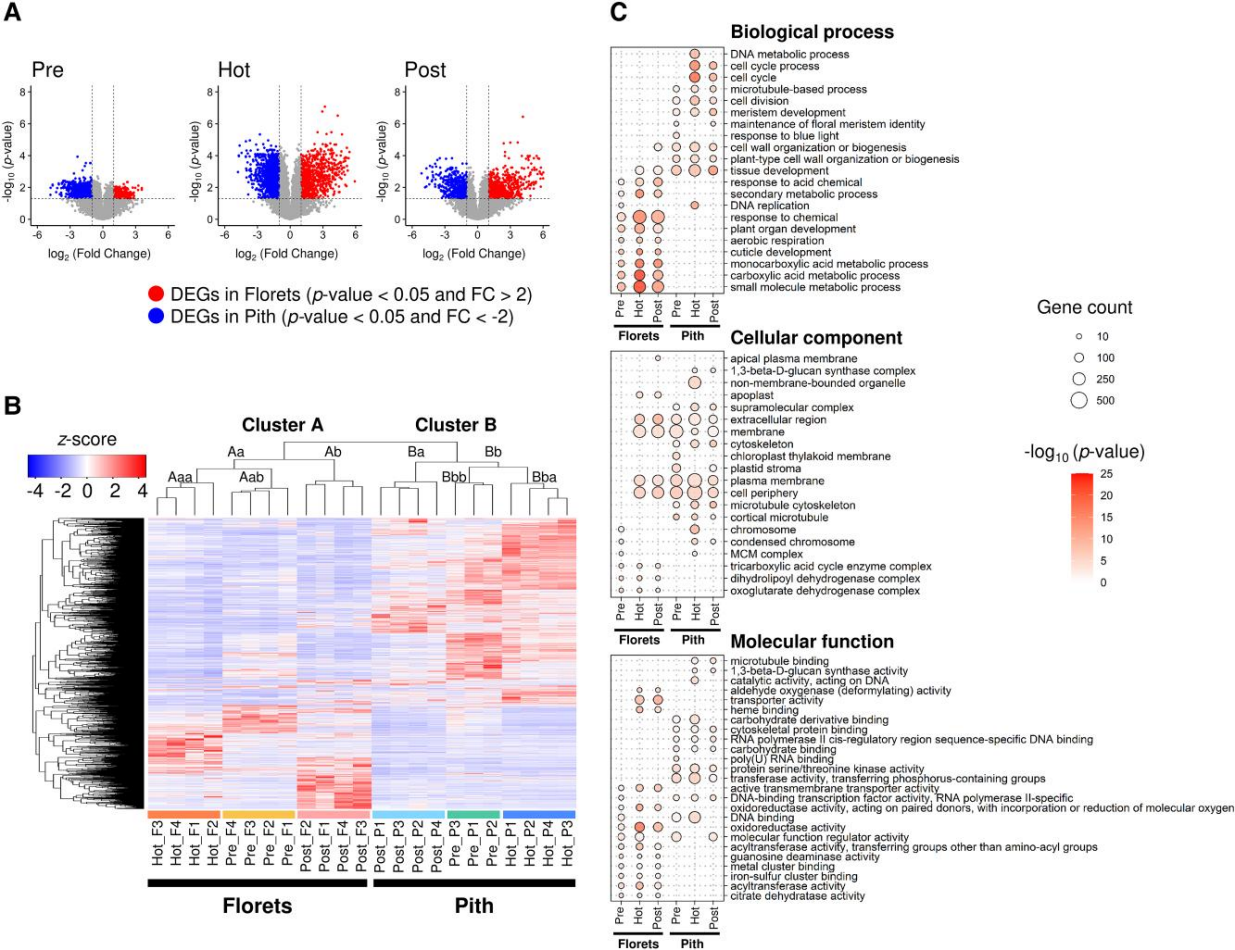
Differentially expressed genes (DEGs) in the thermogenic spadices of *S. renifolius.***A)** Volcano plots highlighting the DEGs between the florets and pith at various thermogenic stages. The threshold for DEG detection [*P*-value < 0.05 and |fold change (FC)| > 2] is shown with dashed lines. **B)** Hierarchical clustering and heatmap analysis of DEGs. Samples are designated by stage and tissue type (Fig. 1C). Normalized expression values are presented as *z*-scores based on TPM values. **C)** GO enrichment analysis for the DEGs, segregated by tissue type (florets or pith) and thermogenic stage in the spadices (Pre, Hot, or Post). Each panel corresponds to 1 of the 3 GO categories: biological process (top), cellular component (middle), and molecular function (bottom). The top 5 enriched GO terms for each tissue type and thermogenic stage were identified, and overlapping terms were excluded from the combined list. Circle size corresponds to the number of genes associated with each term, and the intensity reflects the −log_10_ (*P*-value). The stages of thermogenesis in the spadices are denoted as “Pre” (prethermogenic), “Hot” (thermogenic), and “Post” (postthermogenic). TPM, transcripts per million.

To compare gene expression profiles in thermogenic florets with those in less thermogenic pith, we identified differentially expressed genes (DEGs) for each thermogenic stage (Fig. 2A; Supplementary Fig. S2A and Supplementary Data Set 2). We first created a pooled set (5,081 genes) by combining genes from both the florets and the pith at various thermogenic stages. Redundant genes in this pool, found either in the florets or the pith, were subsequently removed, resulting in the exclusion of 1,457 genes. This analysis revealed a total of 3,624 DEGs, with 1,302 in the florets and 2,322 in the pith (Supplementary Fig. S2B and Supplementary Data Set 3).

We next investigated how the transitions between thermogenic stages associated with the expression patterns of different transcripts. We conducted clustering analysis on the DEGs (Fig. 2B). Hierarchical clustering and heatmap analysis revealed distinct clusters of DEGs, which were characterized by unique expression patterns across different thermogenic stages and tissue types. Specifically, the 23 samples examined could be clearly divided into Cluster A, corresponding to floret tissue, and Cluster B, corresponding to pith tissue. Within Cluster A, which encompasses all floret samples, the prethermogenic (Pre), thermogenic (Hot), and postthermogenic (Post) samples were further grouped into Subclusters Aab, Aaa, and Ab, respectively. Similarly, within Cluster B, less thermogenic pith samples from the Pre, Hot, and Post stages of spadices were categorized into Subclusters Bbb, Bba, and Ba, respectively. While both florets and pith undergo thermal changes during the development of the spadix, it is essential to note here that the pith exhibits a less intense thermogenic activity compared to the florets. Accordingly, our findings suggested that the samples we collected accurately reflected the tissue- and developmental stage-specific aspects of thermogenesis.

To further clarify the relationship between the gene expression profile and the thermogenic stages of the spadix, we performed a principal component analysis (PCA) on DEGs (Supplementary Fig. S3A). In our PCA, all samples collected in the present study were mapped into 6 major clusters, which primarily differentiated them in a tissue- and thermogenic stage-specific manner. Namely, PC1 accounted for 37.1% of the variance, corresponding to the differences between the florets and pith, while PC2 contributed to 23.2% of the separation, associated with transitions between different thermogenic stages (Supplementary Fig. S3A). DEGs specific to either florets or pith clearly contribute to the tissue-specific gene expression levels, as accounted for by PC1 loadings (Supplementary Fig. S3B and Supplementary Data Set 4). However, it is notable that these tissue-specific classifications do not fully explain the variance for thermogenic stage-specific gene expression, as indicated by PC2 loadings (Supplementary Fig. S3B).

To elucidate the potential biological roles of DEGs, particularly their involvement in metabolic processes, we conducted gene ontology (GO) analysis (Fig. 2C). This analysis revealed trends within the biological process category. Genes associated with the metabolic processes of “small molecules”, “carboxylic acids”, and “monocarboxylic acids”, as well as those involved in “aerobic respiration”, “plant organ development”, and “responses to chemicals”, were consistently upregulated in the florets across all developmental stages. This upregulation was notably more pronounced during the thermogenic stage. In this study, “small molecule metabolic process”, as defined in the GO, refers to the small molecules that are characterized by their low molecular weight. These molecules, which include ions, metabolites, and a variety of bioactive compounds, are involved in a range of cellular processes and functions. In contrast, in the pith collected from the thermogenic stage of spadices, referred to as “Pith Hot”, expression levels of genes associated with DNA replication and DNA metabolic processes were exclusively increased, suggesting an upregulation in cell division in the pith tissue at this stage. In the cellular component category, genes associated with components of the oxoglutarate dehydrogenase complex and the tricarboxylic acid (TCA) cycle enzyme complex were consistently found in the florets across all stages. Within the molecular function category, genes associated with oxidoreductase activity were the most intensively expressed genes in the thermogenic florets.

### Gene expression profiles in the florets and pith

**Figure 3.**
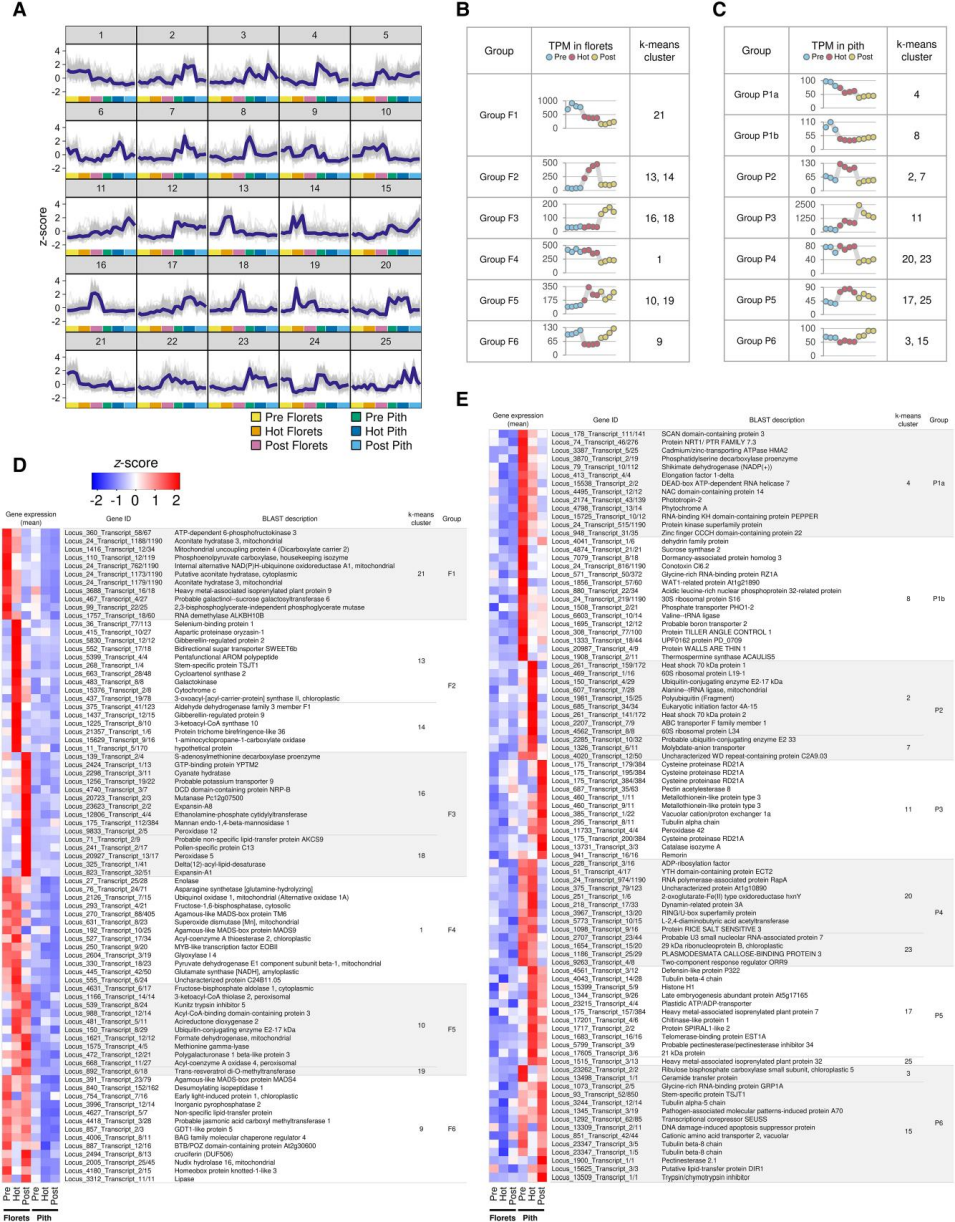
Dynamic gene expression patterns during spadix development *in S. renifolius*. **A)** Nonhierarchical k-means clustering delineates 25 distinct expression profiles for identified differentially expressed genes (DEGs). Individual gene expression trajectories within each profile are depicted in thin lines, with the average trajectory for each cluster highlighted by a bold line. Thermogenic stages and sampled tissues are denoted by color-coded bars along the horizontal axis. **B, C)** Gene expression changes in florets (**B**) and pith (**C**) throughout spadix development. DEGs are organized into 6 distinct groups based on k-means clustering. Homologous gene expression patterns between the 2 tissues are grouped and marked with identical numbers, with “F” denoting florets and “P” indicating pith in both panels. The scatter plots in both panels show TPM values, with each dot representing the calculated average expression level of a gene across analyzed samples. The dots are color-coded to indicate different thermogenic stages, and the corresponding k-means clustering numbers are provided. **D, E)** Representative genes and their expression levels in florets and pith from the groups shown in panels (**B**) and (**C**). The top 5 genes by TPM values at each thermogenic stage for each tissue were selected, with overlaps excluded from the combined list. TPM values are normalized to *z*-scores, with expression levels visualized on a heatmap. Gene identifiers, BLAST descriptions, k-mean clustering numbers, and group identifications are shown. The stages of thermogenesis in the spadices are denoted as “Pre” (prethermogenic), “Hot” (thermogenic), and “Post” (postthermogenic). TPM, transcripts per million.

To further explore the expression profiles of the DEGs, we employed a nonhierarchical k-means clustering approach, which divided them into 25 distinct clusters (Fig. 3A). These clusters represented tissue- and stage-specific expression patterns, providing insights into the dynamic changes in gene expression throughout the transitioning between thermogenic stages. For instance, certain clusters, such as Clusters 13 and 14, demonstrate a pronounced upregulation of gene expression associated with the thermogenic stage of florets. Similarly, Clusters 2 and 7 highlight unique gene expression in the pith during the thermogenic stage of spadices, exemplifying potential specialized roles in supporting the thermogenic activity of the florets or in managing stress responses under elevated temperatures in the pith.

Next, we focused on the expression patterns of DEGs. From the 25 clusters identified (Fig. 3A), we recategorized these into 6 distinct groups. Each group corresponded to a unique thermogenic status, both of the florets (Fig. 3B) and the pith (Fig. 3C). The comprehensive list of genes encompassed within each group is detailed in Supplementary Data Set 5. A particularly notable observation emerged from Group F2, comprising Clusters 13 and 14. This group contained genes that exhibited the highest expression levels during the thermogenic stage, in contrast to relatively lower levels at other stages. Such a distinct expression pattern in Group F2 suggests that the products of these genes potentially play a pivotal role in the thermogenic process. Group F1 encompassed Cluster 21 and contained genes with the highest expression levels at the prethermogenic stage, and their expression progressively decreased with advancing thermogenic stages. Group F3 encompassed Clusters 16 and 18 and contained genes with the highest expression levels in the postthermogenic stage. While these groups were most highly expressed at any one thermogenic stage, we also found groups with similar levels of expression at 2 different stages. Group F4 was derived from Cluster 1, and the expression levels of these genes were maintained from prethermogenic to thermogenic stages and decreased with the end of thermogenesis. Group F5, which included Clusters 10 and 19, contrasted with Group F4 as the expression of its genes was induced with the onset of thermogenesis and maintained at high levels even after thermogenesis ended. Group F6, on the other hand, contained Cluster 9 and showed an expression pattern that was the reverse of that seen in Group F2. We applied a similar k-means clustering approach to the DEGs in the pith, resulting in 6 distinct groups (Groups P1 to P6). Each group distinctly represents gene expression patterns observed at various thermogenic stages (Fig. 3C). The lists of representative genes in each group detail the specific functions of their products, characteristic of each thermogenic stage in both the florets and the pith (Fig. 3D and E, and Supplementary Fig. S4). Notable genes were identified in each group, highlighting their distinct roles; Group F1 includes genes involved in energy metabolism, such as *ATP-dependent 6-phosphofructokinase 3* and *mitochondrial uncoupling protein 4*. In Group F2, the presence of the *bidirectional sugar transporter SWEET6b* suggests a critical role for enhanced carbon flux during the thermogenesis. Group F4 contained *enolase*, *ubiquinol oxidase 1 (AOX)*, *fructose-1,6-bisphosphatase*, and *pyruvate dehydrogenase E1 component subunit beta-1*. These genes related to energy metabolism were expressed prior to spadix temperature increases, in contrast to *A. concinnatum AOX* expression which associated with the tissue temperature (Onda et al. 2015).

Analysis of the pith identified several genes with noteworthy expression patterns. For instance, in Group P1b, *sucrose synthase 2* is notable for its expression during the prethermogenic stage. The presence of the genes *heat shock 70 kDa protein 1* and *2* in Group P2 indicates that the increased temperature induces temperature stress in the less thermogenic pith tissues. Notably, within Group P3, the presence of the gene *cysteine (Cys) protease RD21A* highlights the potential role of its product in the postthermogenic pith tissues. For Groups P4 to P6, *YTH domain-containing protein ECT2* (Group P4), *defensin-like protein P322* (Group P5), and *glycine-rich RNA binding protein GRP1A* (Group P6) were found (Fig. 3E). The list of genes, along with their expression profiles across the thermogenic stages and GO enrichment analyses, is presented in Supplementary Data (Supplementary Figs. S4 and S5). The full dataset of the DEGs categorized by k-means clustering is provided in Supplementary Data Set 5.

We further investigated whether the individual groups, as classified above, contributed to the PCA variance related to tissue- and thermogenic stage-specific gene expression (Supplementary Fig. S3). Our results confirmed that each group associated with the florets (Groups F1 to F6) and pith (Groups P1a and P1b to P6) substantially contributed to the tissue-specific gene expression, as indicated by PC1 loadings (Supplementary Fig. S6A). In contrast, DEGs in Groups F1 to F4 and Groups P1a, P3, P5, P6 appeared to contribute more to the thermogenic stage-specific gene expression, as indicated by PC2 loadings (Supplementary Fig. S6B). Detailed data for this analysis can be found in Supplementary Data Set 6.

### Identifying the 10 genes with the highest expression levels in the florets and pith during thermogenic stage of spadices

**Figure 4.**
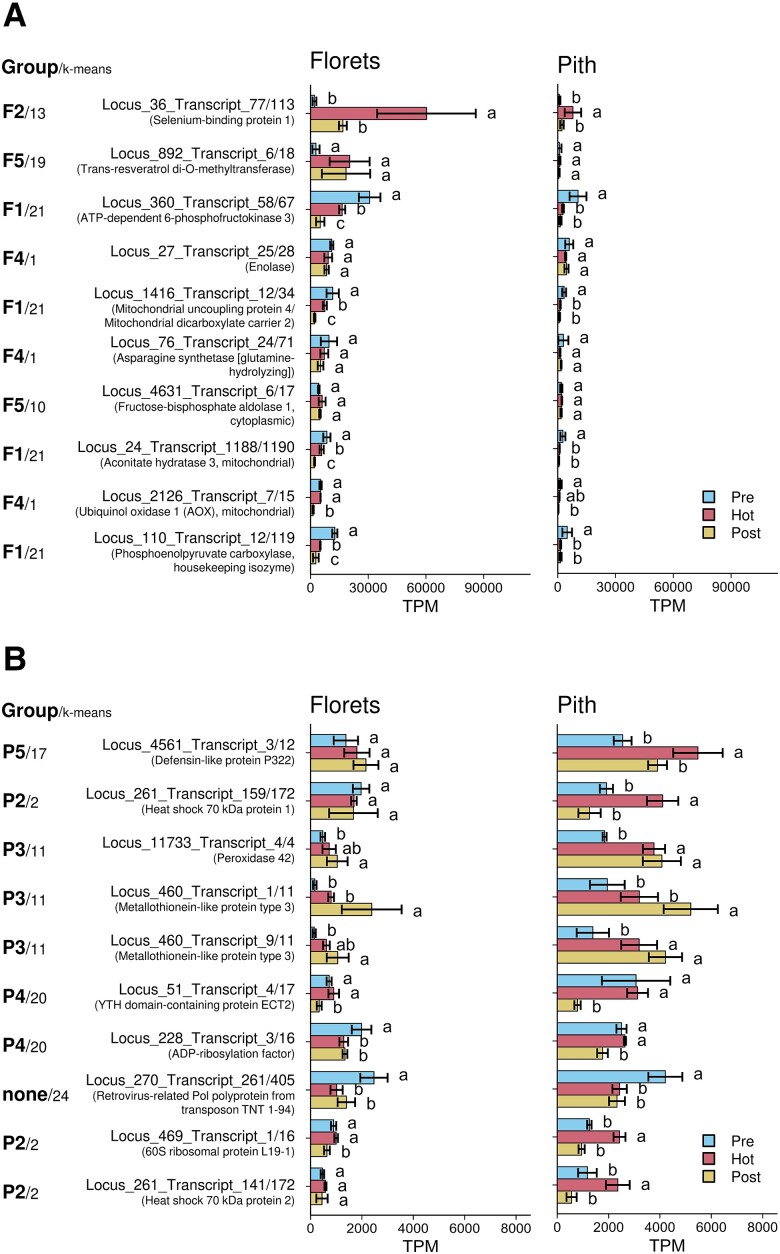
Analysis of the top 10 differentially expressed genes (DEGs) in the florets and pith during the thermogenic stage of spadices in *S. renifolius.* Comparisons of TPM values across prethermogenic (Pre), thermogenic (Hot), and postthermogenic (Post) stages of spadices for **A**) florets and **B**) pith are shown. Genes are listed according to their expression levels, and corresponding values for the prethermogenic and postthermogenic stages are also shown for comparison. The bar graphs represent the average TPM values of multiple independent samples (*n* = 4 except for prethermogenic pith: *n* = 3) ± standard deviation. Statistically significant differences, as determined by the Tukey–Kramer test (*P* < 0.05), are denoted by different letters beside the bar plots. The gene categorization based on group and k-means cluster identification (Fig. 3B and C), is indicated to the left of each panel. Gene identifiers and putative functions are also given. TPM, transcripts per million.

Our RNA-seq data identified highly expressed genes in the florets and pith of thermogenic spadices in *S. renifolius*. The top 10 genes, including their expression in the prethermogenic, thermogenic, and postthermogenic stages of spadices, are displayed in the florets (Fig. 4A) and in the pith (Fig. 4B), respectively. In the florets, the gene encoding selenium-binding protein 1 (SBP1) exhibited the highest expression level, followed by the gene encoding *trans*-resveratrol di-*O*-methyltransferase (ROMT). The gene encoding mitochondrial uncoupling protein 4/mitochondrial dicarboxylate carrier 2 was found to have the fifth highest expression level in thermogenic florets, and the gene for AOX was ranked 9th. Notably, among the top 10 genes expressed in the thermogenic florets, 4 are associated with the glycolytic pathway, including *phosphofructokinase kinase* (*PFK*), *enolase*, *aldolase*, and *phosphoenolpyruvate carboxylase* (*PEPC*), along with *aconitase*, which is part of the TCA cycle. The transcript levels of *SBP1*, *ROMT*, and *AOX* among the top 10 genes were validated using reverse transcription quantitative PCR (RT-qPCR); Supplementary Fig. S7). In contrast, in the pith of thermogenic spadices, *defensin-like protein P322* exhibited the highest expression, followed by *heat shock 70 kDa protein 1*, *peroxidase 42*, and *metallothionein-like protein type 3* (Fig. 4B).

### Metabolomic analysis

Comprehensive metabolomic analysis was conducted utilizing the same set of samples that were employed for the transcriptome analysis (Supplementary Fig. S1). This enabled the assessment of the tissue- and stage-specific accumulation of 93 metabolites, encompassing a broad range of categories such as sugars, organic acids, amino acids, and polyamines (Supplementary Tables S2 and S3, and Supplementary Data Set 7). Upon examination of the 3 thermogenic stages in each tissue, substantial alterations were observed in the metabolite accumulation profiles (Supplementary Fig. S8).

**Figure 5.**
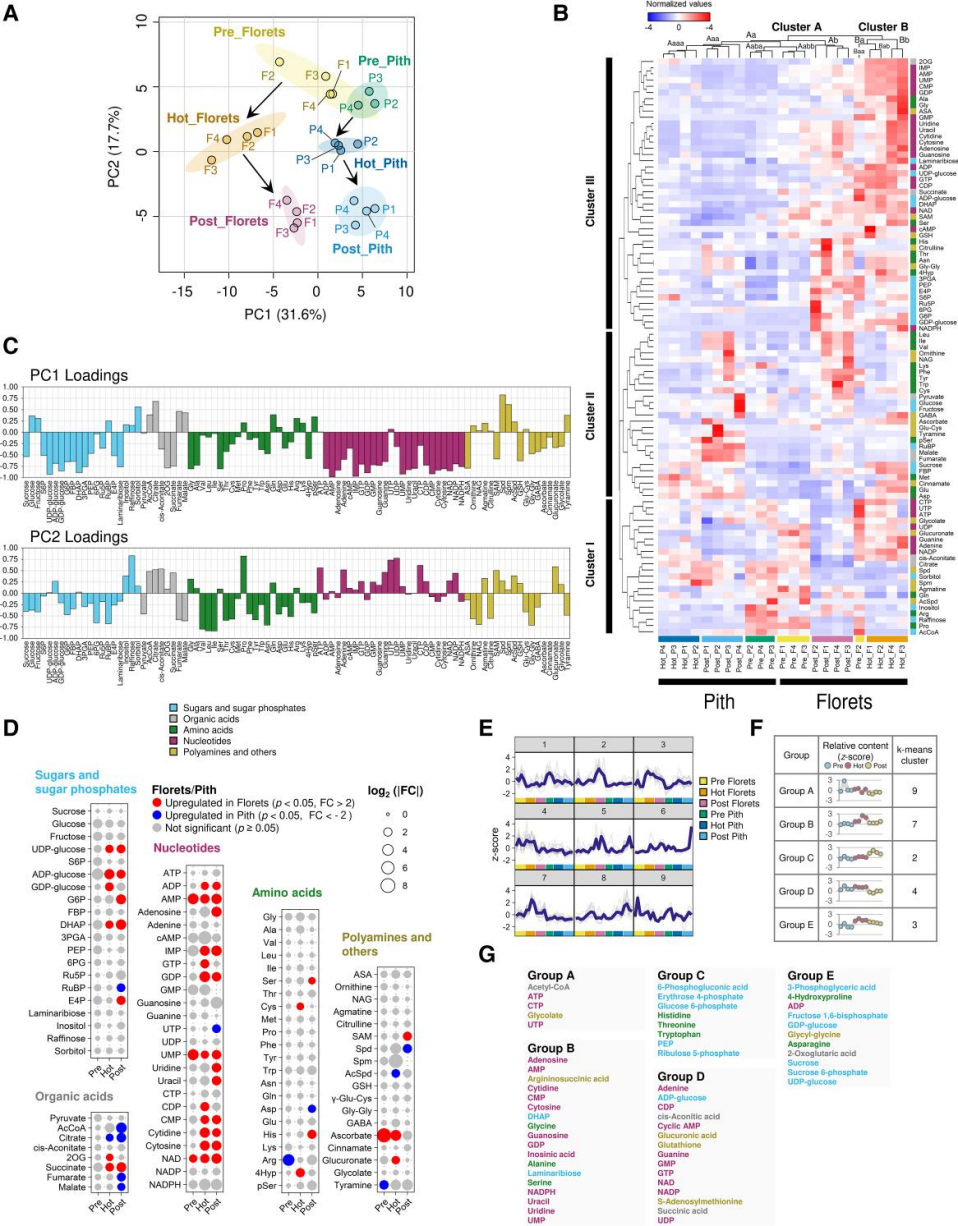
Metabolite profiling of florets and pith across various thermogenic stages in the spadices of *S. renifolius*. **A)** Principal component analysis (PCA) plot of the analyzed metabolites. PC1 (31.6%) contributes to the separation of tissues with thermogenic status, while PC2 (17.7%) reflects the transition of thermogenic stages. Colored areas for each stage and tissue represent 95% confidence intervals (MetaboAnalyst). Arrows indicate the developmental progression from the prethermogenic (Pre), through thermogenic (Hot), to postthermogenic (Post) stages of spadices in both florets and pith. **B)** Hierarchical clustering diagrams of metabolites, grouped according to a dissimilarity scale, are presented on the top (Clusters A and B) and on the left (Clusters I, II, and III). Metabolites, categorized as sugars and sugar phosphates, organic acids, amino acids, nucleotides, polyamines, and others, are denoted by different color labels. Each sample is identified by its thermogenic stage (Pre, Hot, or Post) and tissue type (F for florets, P for pith), as represented by respective identifiers (e.g. Hot_P4 refers to the 4th pith sample collected from the thermogenic stage of spadices). Normalized data of metabolite concentrations are represented by the *z*-score. **C)** PC loading values of metabolites in PC1 and PC2 from panel (**A**). Sugars and sugar phosphates, organic acids, amino acids, nucleotides, polyamines, and others are differentiated by colors as shown in the panel. **D)** Accumulation of metabolites expressed as fold changes in prethermogenic (Pre), thermogenic (Hot), and postthermogenic (Post) stages of spadices. Metabolites categorized as sugars and sugar phosphates, organic acids, amino acids, nucleotides, polyamines, and others are shown. Upregulated metabolites either in florets or pith are depicted with their fold changes in different colors. The log_2_ (|fold change (FC)|) is represented by the size of the circles, as indicated in the panel. **E)** A visualization of the changes in metabolite accumulation from prethermogenic (Pre), through thermogenic (Hot), to postthermogenic (Post) stages. Metabolites are organized into 9 distinct groups using k-means clustering. **F)** A representative display of metabolite accumulation profiles (Groups A to E) corresponding to the diverse stages of thermogenesis in the florets, alongside their respective k-means cluster numbers. Each stage of thermogenesis includes 4 biological replicates, each represented as a separate dot based on the *z*-score. **G)** Metabolites within each group are categorized into sugars and sugar phosphates, organic acids, amino acids, nucleotides, polyamines, and others, each of which is represented by a unique color.

To explore the temporal and spatial dynamics of metabolic shifts in the florets and pith tissues from prethermogenic to postthermogenic stages of spadices, we first conducted a PCA. This analysis revealed that PC1, accounting for 31.6% of the variance, represented tissue-specific differences. Conversely, PC2, which was responsible for 17.7% of the variance, corresponded to the transitions occurring between the thermogenic stages (Fig. 5A). Intriguingly, our data indicated that 6 clusters, each representing a thermogenic stage and tissue, exhibited a clear progression as indicated by the arrows in the panel (Fig. 5A). Specifically, both the florets and pith samples from prethermogenic stage of spadices were located at the top of the panel, demonstrating a degree of overlap. As the thermogenic stage advanced, each sample was found to occupy distinct positions.

We next subjected our metabolite data to hierarchical clustering analysis (HCA). The dendrogram that we derived from the variations in the examined metabolites, confirmed the results observed with PCA and provided a more detailed view of the tissue-specific differences between the florets and the pith (Fig. 5B). The HCA identified 2 major clusters, labeled A and B. Cluster A consisted of the pith tissue samples from spadices at prethermogenic, thermogenic, and postthermogenic stages, along with samples from the florets of prethermogenic and postthermogenic spadices. In contrast, Cluster B encompassed 4 samples from the florets of a thermogenic spadix (Hot_F1-F4) and one sample from the pith of prethermogenic florets (Pre_F2). This cluster could be further divided into 3 Subclusters: Baa, Bab, and Bb. Subcluster Baa contained a florets sample from a prethermogenic spadix (Pre_F2), while Bab and Bb included the 4 florets samples from thermogenic spadices (Hot_F1 to F4). Notably, the sample Pre_F2 was separated from the remaining 3 florets samples from prethermogenic spadices and positioned at the far left in the HCA plot (Fig. 5A). Indeed, the temperature difference between the spadix and the ambient environment for Pre_F2 was the largest among the 4 prethermogenic spadices studied, as detailed in Supplementary Table S1. This observation suggested that weak thermogenesis may have been occurring during our sampling, indicating that the Pre_F2 sample may represent an intermediate stage in progression toward the thermogenic stage. It is also worth noting that the gene expression profile of Pre_F2 fell into the same cluster as other florets samples from prethermogenic spadices (Fig. 2B and Supplementary Fig. S3A), suggesting that alterations in metabolite levels could potentially serve as a more direct and acute indicator of the initiation of thermogenesis in the florets. Moreover, our HCA analyses separated the examined metabolites into 3 distinct clusters (I to III; Fig. 5B). Of particular interest, Cluster III could be characterized by a marked accumulation of nucleotides, including AMP and adenosine, in thermogenic florets.

Furthermore, the profile of the PC1 loadings differed substantially from that of PC2 (Fig. 5C). It should be noted here that nucleotides were predominantly located on the negative side of the PC1 loadings, while they appeared on the positive side of the PC2 loadings.

We further explored the specific metabolites that exhibited differential accumulation in the florets compared with the pith (Fig. 5D and Supplementary Fig. S8). Of the metabolites examined, 2-oxoglutarate (2OG), guanosine triphosphate (GTP), cytidine diphosphate (CDP), cysteine (Cys), 4-hydroxyproline (4Hyp), and glucuronate were markedly upregulated in the florets, displaying a more than 2-fold increase in accumulation compared to in the pith (Fig. 5D). It is important to note here that there appeared to be a consistent trend for the florets to accumulate higher levels of nucleotides throughout the thermogenic stages.

In addition to the PCA, our analysis with nonhierarchical clustering identified 9 distinct clusters for changes in metabolites (Fig. 5E). This analysis effectively segregated the metabolite accumulation profiles in the florets into 5 discrete groups (A–E) (Fig. 5F and G). The groupings reflected the relative metabolite contents across the prethermogenic, thermogenic, and postthermogenic florets. In particular, Group B, which showed a thermogenic stage-specific increase in accumulation levels across the 3 stages, was primarily composed of nucleotides, including adenosine, AMP, and NADPH. Notably, this group also includes alanine (Ala), which may act as a precursor for mitochondrial pyruvate, potentially leading to the activation of AOX (Le et al. 2022). Group C, representing metabolites primarily accumulated in the postthermogenic stage, consisted mainly of sugar phosphates, such as 6-phosphogluconic acid (6PG), erythrose 4-phosphate (E4P), glucose 6-phosphate (G6P), phospho*enol*pyruvate (PEP), and ribulose 5-phosphate (R5P).

**Figure 6.**
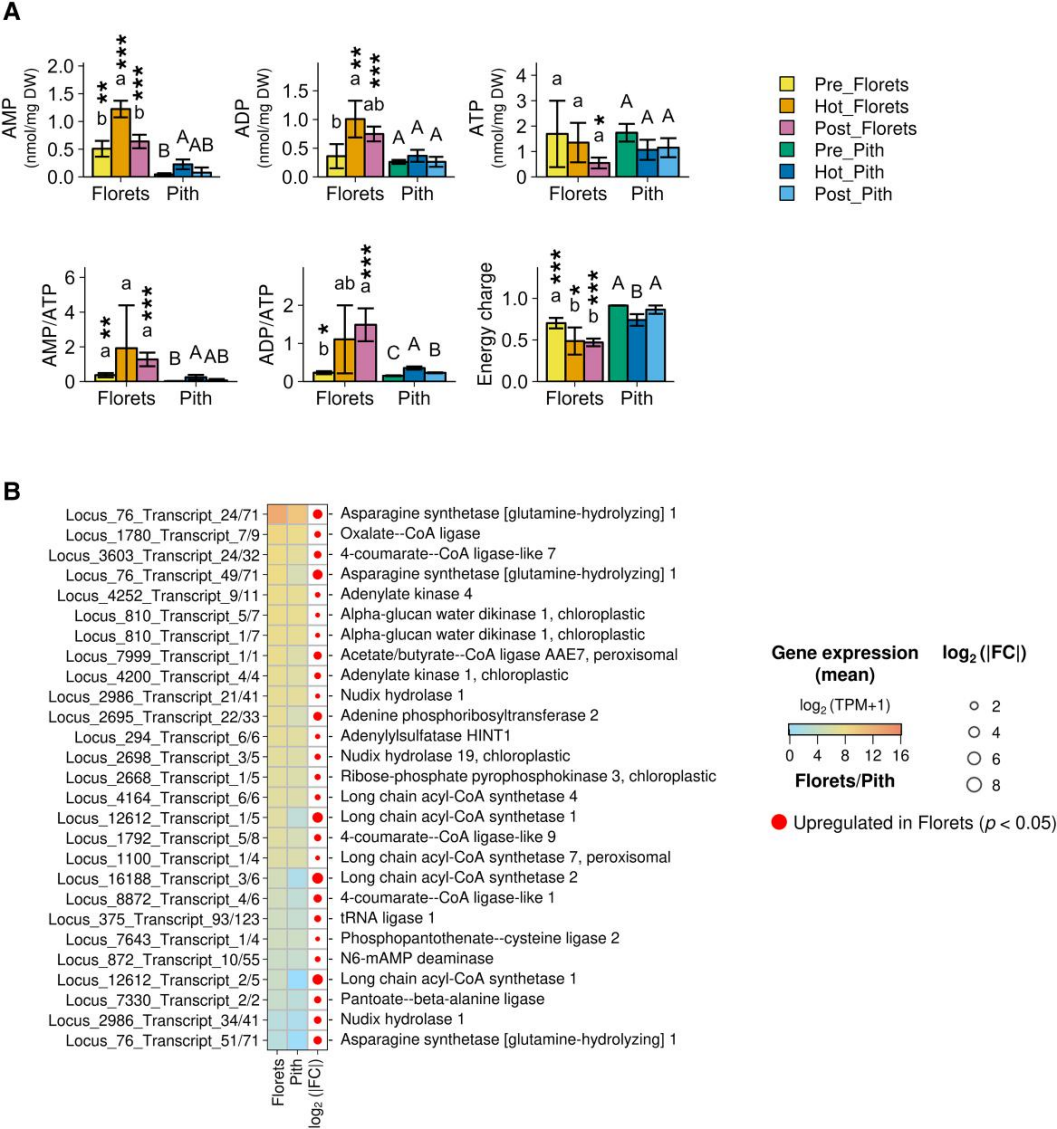
Changes in the accumulation of adenylates and genes for AMP production in the spadices of *S. renifolius*. **A)** A comparison of adenylate nucleotide levels across prethermogenic (Pre), thermogenic (Hot), and postthermogenic (Post) stages of spadices in florets and pith. Data for AMP, ADP, ATP levels, AMP/ATP, ADP/ATP ratios, and energy charge are presented. Energy charge is defined by the following formula: (ATP + ½ ADP)/(ATP + ADP + AMP). The mean values and standard deviations from independent measurements (*n* = 4 except for Pre_Pith *n* = 3) are presented in bar graphs with error bars, respectively. Statistically significant differences are denoted by different letters above the bars (Tukey–Kramer test: *P* < 0.05). Specifically, lowercase letters denote comparisons within the “florets” group, while uppercase letters are used for comparisons within the “pith” group. Significant differences between the florets and the pith are indicated by the number of asterisks: 1 asterisk (*) denotes *P* < 0.05, 2 asterisks (**) denote *P* < 0.01, and 3 asterisks (***) denote *P* < 0.005, as determined by Student's *t*-test. Each thermogenic stage (Pre, Hot, or Post) and tissue type (florets or pith) are differentiated by distinct colors. **B)** List of genes potentially contributing to AMP production in the thermogenic florets, ranked by their expression levels. Genes that are upregulated in florets, with fold changes indicated by the size of the circles, are displayed. Averaged gene expression levels (*n* = 4), denoted by (log_2_ (TPM) + 1), are shown. Circles of different sizes represent the log_2_ fold change (FC) values, ranging from 2 to 8. Filled circles indicate upregulation in the florets. Gene identifiers and their putative functional names are also provided. DW, dry weight; TPM, transcripts per million.

Given that adenosine phosphates (AMP, ADP, and ATP) play pivotal roles in cellular bioenergetics, we next investigated their levels, as well as the AMP/ATP and ADP/ATP ratios, and the energy charge in the florets and pith at various stages of the spadices (Fig. 6A). Our results revealed that only the AMP level in thermogenic florets significantly increased over the 3 thermogenic stages, and between the florets and the pith. Although there was no significant difference in energy charge values between thermogenic and postthermogenic florets, a notable decrease was observed when the thermogenic florets were compared with the prethermogenic florets. These results prompted an exploration of the genes encoding enzymes involved in the production of AMP (Fig. 6B). Our analyses identified 27 genes that showed higher expression in the florets than in the pith at the thermogenic stage. The most highly expressed gene on this list was for asparagine (Asn) synthetase, which hydrolyzes glutamine to produce Asn, simultaneously hydrolyzing ATP to AMP. The list also contained the genes for oxalate CoA ligase, 4-coumarate CoA ligase-like, and adenylate kinase. These results suggested the potential involvement of various genes related to AMP production in the thermogenic stage of the florets.

### Integrated transcriptome and metabolome analyses at the thermogenic stage of the spadix

**Figure 7.**
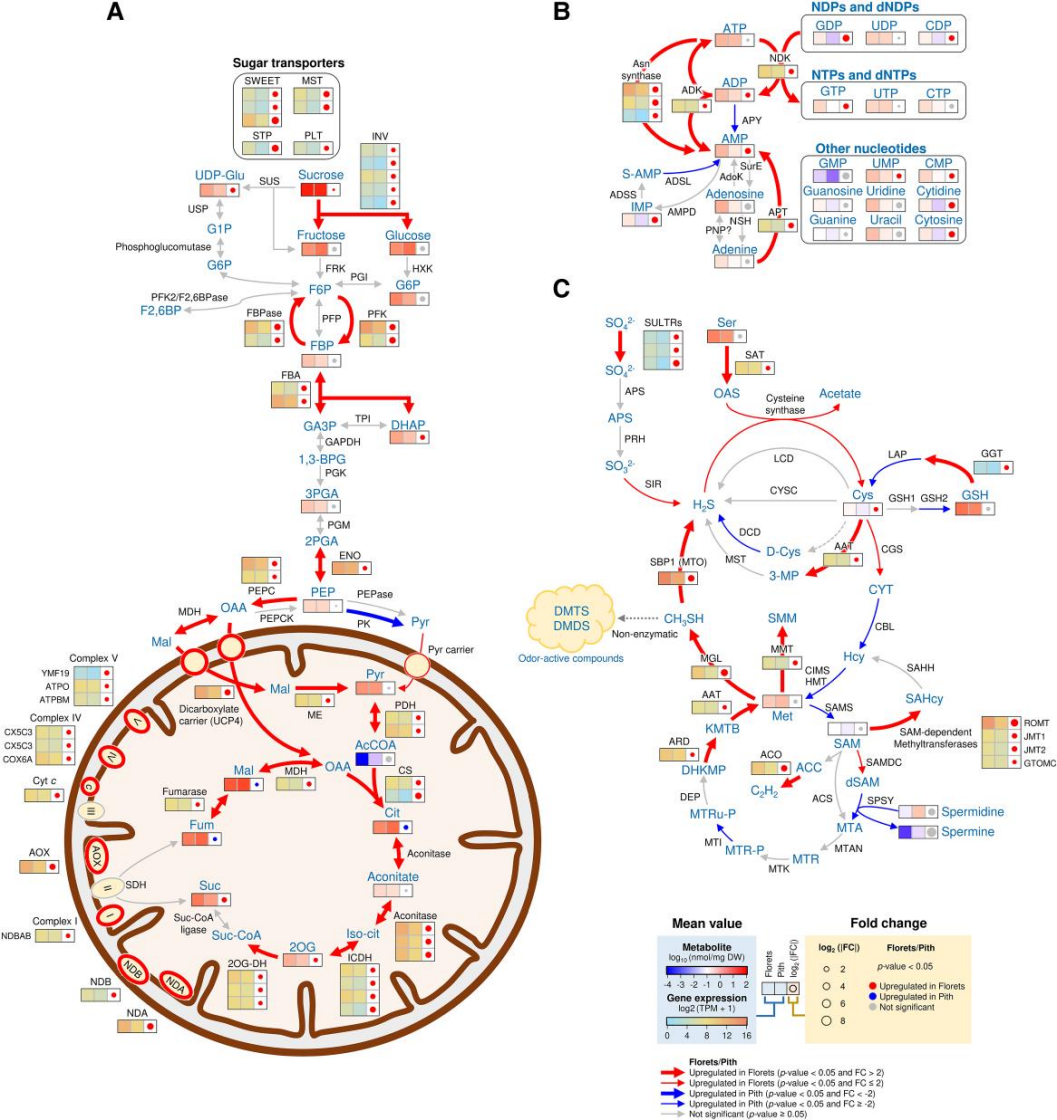
Integrated overview of transcriptome and metabolome analyses in the thermogenic florets of *S. renifolius*. **A)** A comprehensive map of the central carbon metabolic pathways and mitochondrial metabolism. **B)** Interconversion of adenylate nucleotides and the activation of associated pathways. **C)** An overview of sulfur and methionine metabolism. The diagrams in panels (**A**) to (**C**) incorporate information on gene expression and metabolite accumulation in the florets and pith at the thermogenic stage of spadices. Color gradients in the lower panel (**C**) show variations in metabolite accumulation (log_10_ (nmol/mg DW)) and gene expression levels (log_2_ (TPM) + 1). Circles of different sizes represent the log_2_ fold change (FC) values, ranging from 2 to 8. Filled red circles indicate upregulation in the florets, blue filled circles represent upregulation in the pith, and gray filled circles denote nonsignificant differences. Arrows denote upregulation trends in either the florets (red) or the pith (blue), with bold arrows indicating a *P*-value < 0.05 and FC > 2 or FC < −2. Narrow arrows show a *P*-value < 0.05 and FC ≤ 2 or FC ≥ −2, and gray arrows point to nonsignificant results (*P*-value ≥ 0.05). DW, dry weight; TPM, transcripts per million. 2OG-DH, 2-oxoglutarate dehydrogenase; ACO, aconitate hydratase; AOX, alternative oxidase; ATPBM, ATP synthase subunit beta; ATPO, ATP synthase subunit O; CS, citrate synthase; CX5C3, cytochrome *c* oxidase subunit 5C-3; COX6A, cytochrome *c* oxidase subunit 6a; Cyt c, cytochrome *c*; dicarboxylate carrier (UCP4), dicarboxylate carrier (mitochondrial uncoupling protein 4); ENO, enolase; FBA, fructose-bisphosphate aldolase; FBPase, fructose-1,6-bisphosphatase; FRK, fructokinase; Fumarase, fumarate hydratase; GAPDH, glyceraldehyde-3-phosphate dehydrogenase; HEX, hexokinase; ICDH, isocitrate dehydrogenase; INV, invertase; ME, malic enzyme; MST, monosaccharide transporter; NDA, internal alternative NAD(P)H:ubiquinone oxidoreductase; NDB, external alternative NAD(P)H:ubiquinone oxidoreductase; NDBAB, NADH dehydrogenase [ubiquinone] 1 beta subcomplex subunit 10-B; PDH, pyruvate dehydrogenase; PEPase, phospho*enol*pyruvate phosphatase (bifunctional purple acid phosphatase); PEPC, phospho*enol*pyruvate carboxylase; PEPCK, phospho*enol*pyruvate carboxykinase; PFK, ATP-dependent 6-phosphofructokinase; PFK2/F2,6BPase, phosphofructokinase-2/fructose-2,6-bisphosphatase; PFP, pyrophosphate-fructose 6-phosphate 1-phosphotransferase; PGI, glucose-6-phosphate isomerase; PGK, phosphoglycerate kinase; PGM, phosphoglycerate mutase; PK, pyruvate kinase; PLT, polyol transporter; Pyr carrier, mitochondrial pyruvate carrier; STP, sugar transport protein; Suc-CoA ligase, succinate-CoA ligase; SUS, sucrose synthase; SWEET, sugars will eventually be exported transporter; UCP, mitochondrial uncoupling protein; USP, UDP-sugar pyrophosphorylase; TPI, triosephosphate isomerase; 1,3-BPG, 1,3-bisphosphoglycerate; 2OG, 2-oxoglutarate; 2PGA, 2-phosphoglycerate; 3PGA, 3-phosphoglycerate; AcCOA, acetyl-CoA; Cit, citrate; DHAP, dihydroxyacetone phosphate; F2,6BP, fructose 2,6-bisphosphate; F6P, fructose 6-phosphate; FBP, fructose 1,6-bisphosphate; Fum, fumarate; G1P, glucose-1-phosphate; G6P, glucose 6-phosphate; GA3P, glyceraldehyde 3-phosphate; Iso-cit, isocitrate; Mal, malate; OAA, oxaloacetate; PEP, phospho*enol*pyruvate; Pyr, pyruvate; Suc, succinate; Suc-CoA, succinyl-CoA; UDP-Glu, uridine diphosphate glucose; ADK, adenylate kinase; ADSL, adenylosuccinate lyase; ADSS, adenylosuccinate synthetase; AMPD, AMP deaminase; APY, apyrase; Asn synthase, asparagine synthetase; APT, adenine phosphoribosyltransferase; adoK, adenosine kinase; NDK, nucleoside diphosphate kinase; NSH, ribonucleoside hydrolase; PNP, purine nucleoside phosphorylase; SurE, 5′-nucleotidase SurE; ADP, adenosine diphosphate; AMP, adenosine monophosphate; ATP, adenosine triphosphate; CDP, cytidine diphosphate; CMP, cytidine monophosphate; CTP, cytidine triphosphate; GDP, guanosine diphosphate; GMP, guanosine monophosphate; GTP, guanosine triphosphate; IMP, inosine monophosphate; S-AMP, adenylosuccinate; UDP, uridine 5′-diphosphate; UMP, uridine 5′-monophosphate; UTP, uridine triphosphate. AAT, aspartate aminotransferase; ACCO, 1-aminocyclopropane-1-carboxylate oxidase; ACCS, 1-aminocyclopropane-1-carboxylate synthase; APS, ATP sulfurylase; ARD, 1,2-dihydroxy-3-keto-5-methylthiopentene dioxygenase; CBL, cystathionine beta-lyase; CGS, cystathionine gamma-synthase; CIMS, 5-methyltetrahydropteroyltriglutamate-homocysteine methyltransferase; CYSC, bifunctional L-3-cyanoalanine synthase/cysteine synthase; DCD, D-cysteine desulfhydrase; DEP, bifunctional methylthioribulose-1-phosphate dehydratase/enolase-phosphatase E1; GGT, glutathione hydrolase; GSH1, glutamate-cysteine ligase; GSH2, glutathione synthetase; GTOMC, tocopherol *O*-methyltransferase; HMT, homocysteine *S*-methyltransferase; JMT, jasmonic acid carboxyl methyltransferase; LAP, leucine aminopeptidase; LCD, L-cysteine desulfhydrase; MGL, methionine gamma-lyase; MMT, methionine *S*-methyltransferase; MTAN, 5′-methylthioadenosine/*S*-adenosylhomocysteine nucleosidase; MTK, methylthioribose kinase; MTI, methylthioribose-1-phosphate isomerase; PRH, 5′-adenylylsulfate reductase; ROMT, *trans*-resveratrol di-*O*-methyltransferase; SAHH, adenosylhomocysteinase; SAMDC, *S*-adenosylmethionine decarboxylase; SAMS, *S*-adenosylmethionine synthase; SBP1/MTO, selenium-binding protein 1/methanethiol oxidase; SIR, sulfite reductase 1 [ferredoxin]; SPSY, spermine synthase; SULTR, sulfate transporter; MST, thiosulfate/3-mercaptopyruvate sulfurtransferase; 3-MP, 3-mercaptopyruvate; ACC, 1-aminocyclopropane-1-carboxylate; APS, adenosine 5′-phosphosulfate; C_2_H_2_, ethylene; CH_3_SH, methanethiol; Cys, L-cysteine; CYT, cystathionine; D-Cys, D-cysteine; DHKMP, 1,2-dihydroxy-3-keto-5-methylthiopentene; DMDS, dimethyl disulfide; DMTS, dimethyl trisulfide; GSH, glutathione; H_2_S, hydrogen sulfide; Hcy, homocysteine; KMTB, α-ketomethylthiobutyrate; Met, L-methionine; MTA, 5-methylthioadenosine; MTR, 5-methylthioribose; MTR-P, 5-methylthioribose 1-phosphate; MTRu-P, 5-methylthioribulose-1-phosphate; OAS, O-acetylserine; SAHcy, *S*-adenosyl-L-homocysteine; SAM, *S*-adenosylmethionine; Ser, L-serine; SMM, *S*-methylmethionine; SO_3_^2−^, sulfite; SO_4_^2−^, sulfate; dSAM, *S*-adenosylmethioninamine (decarboxy-AdoMet).

We next focused on a comparative analysis of gene expression and metabolite accumulation in the florets and the pith in the same thermogenic spadix. This in-depth comparison uncovered specific changes associated with the molecular processes in the florets of the thermogenic spadix. Our results clearly indicated an upregulation of carbohydrate metabolism, including glycolysis, the subsequent TCA cycle, and the electron transport pathway, in the thermogenic florets (Fig. 7A). Our data indicate a potential involvement of a futile cycle in the florets, comprising ATP-dependent 6-phosphofructokinase (PFK) and fructose-1,6-bisphosphatase (FBPase). Transcript levels for *PFK* and *FBPase* were examined using RT-qPCR, which demonstrated their increased coexpression with *AOX* (Supplementary Fig. S9). Nevertheless, to fully understand the biological implications of the futile cycle in thermogenic florets, further research is needed to elucidate its specific biochemical functions and physiological roles in *S. renifolius*.

We also explored the metabolic pathways for nucleotides in the thermogenic florets (Fig. 7B). Our findings revealed a thermogenic floret-specific increase in the AMP level that was accompanied by an upregulated expression of AMP-producing pathways such as Asn synthase and ADK. This appeared to occur through the activation of interconversions between nucleotides, which was executed by nucleoside diphosphate kinase (NDK). Conversely, the pentose phosphate, pyrimidine nucleotide synthesis, and purine nucleotide synthesis pathways were relatively inactive in the thermogenic florets (Supplementary Fig. S10). Given that the total adenylate accumulation levels remained relatively constant and did not depend on thermogenic status (Supplementary Fig. S11), it seems that the interconversion of nucleotides, rather than their de novo synthesis, plays a crucial role in AMP accumulation in the thermogenic florets. Detailed data on the nucleotide levels are provided in Supplementary Data Set 7.

As previously noted, the most highly expressed gene in the thermogenic florets was *SBP1* (Fig. 4A). SBP1 could potentially serve 2 functions: as a selenium (Se) binding protein (SBP) or as a methanethiol oxidase (MTO) (Eyice et al. 2018; Pol et al. 2018). If SBP1 functions as an SBP, the Se content in the thermogenic spadix would be expected to be high, corresponding to the highest expression of SBP1 in this organ. However, our analysis revealed that the Se levels across various organs, including the spadix, during the thermogenic stage were undetectable (Supplementary Table S4). SBP1 may therefore function as an MTO in *S. renifolius* thermogenesis. Our alignment of the amino acid sequence for SrSBP1, isolated from the thermogenic florets of *S. renifolius*, with those of bacterial, *Caenorhabditis elegans*, and human MTOs, which have previously been demonstrated to function as MTOs (Eyice et al. 2018; Pol et al. 2018; Philipp et al. 2022; Philipp et al. 2023), revealed that residues essential for MTO activity are well conserved in SrSBP1 (Supplementary Fig. S12). Consequently, we henceforth refer to our identified gene, originally termed *SBP1*, as *SBP1/MTO* to reflect the potential dual roles of its product.

This presumption of the MTO role prompted us to further investigate sulfur metabolism in the thermogenic florets using transcriptome and metabolome data. We thereby observed a coordinated upregulation of pathways involved in methionine metabolism. More specifically, we observed an elevation in expression for 2 genes encoding pivotal enzymes within this process—aspartate aminotransferase, which produces methionine, and methionine gamma-lyase, responsible for its degradation (Fig. 7C). Collectively, these enzymes accelerate the production of methanethiol, a substrate for MTO. If MTO activity is low, methanethiol can undergo a nonenzymatic conversion into odor-active compounds such as DMTS and DMDS. However, if SBP1/MTO functions as an MTO, an increase in hydrogen sulfide concentrations within thermogenic florets would be anticipated. Based on this hypothesis, we subsequently examined the concentration of hydrogen sulfide and elucidated its impact on mitochondrial respiration, as detailed in the next section.

### Hydrogen sulfide concentration and its impact on mitochondrial respiration in thermogenic florets

**Figure 8.**
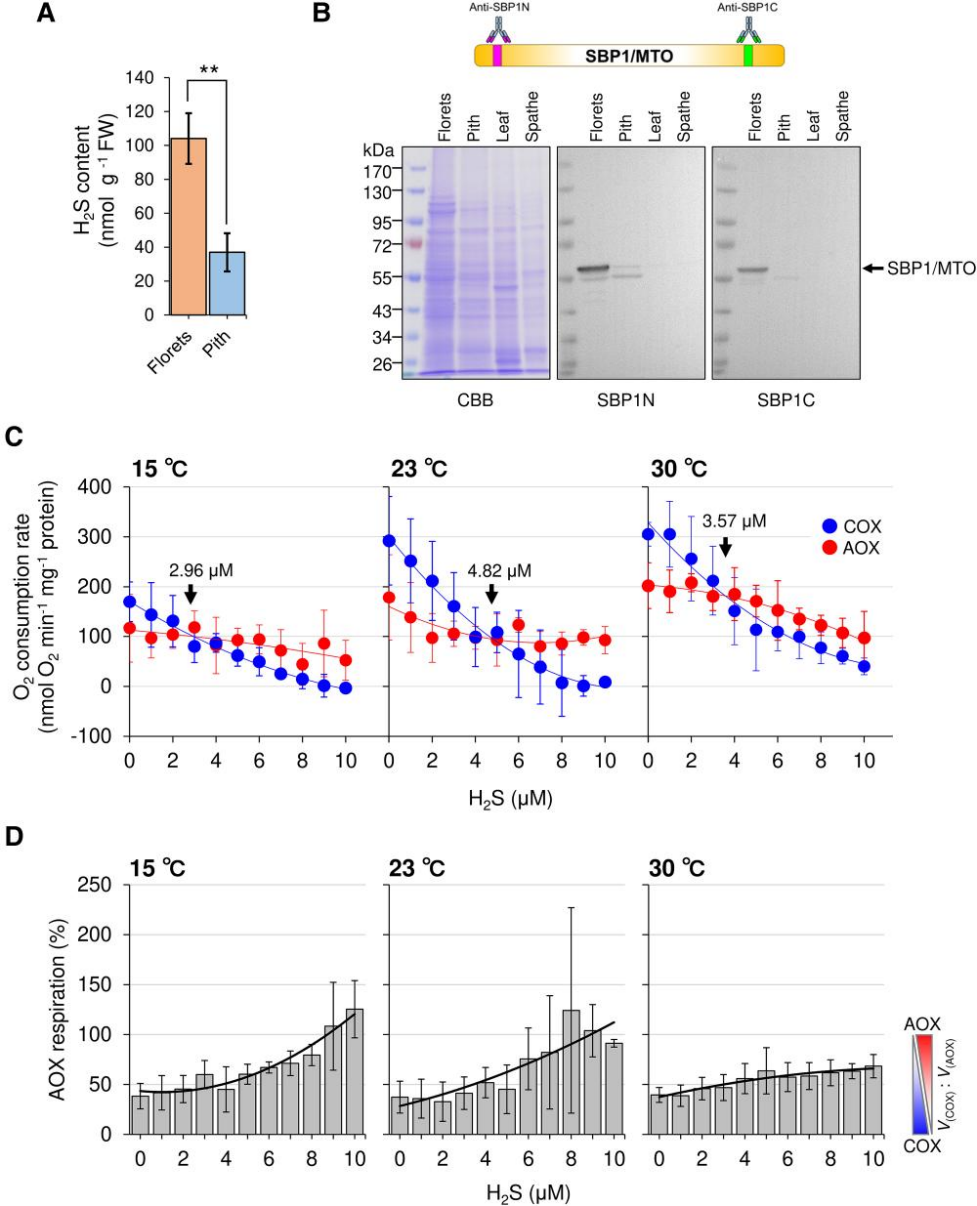
Impact of hydrogen sulfide on the respiration capacities of terminal oxidases in the mitochondria from thermogenic florets of *S. renifolius*. **A)** Endogenous hydrogen sulfide content in the thermogenic stage of the spadix. The analysis was performed on the florets and pith from an *S. renifolius* thermogenic spadix. Results are presented from 3 biological replicates. The error bars show the mean ± standard deviation. Statistically significant differences are indicated by 2 asterisks based on Welch’s *t*-test (*P* = 0.00434). **B)** Western blot analysis using total proteins extracted from thermogenic florets. Two primary antibodies targeting different epitopes, one for the N-terminal region (SBP1N) and the other for the C-terminal region (SBP1C) of the SBP1/MTO protein, were used. The protein loading was standardized to 20 μg per lane. Coomassie Brilliant Blue (CBB) staining is shown for reference. **C)** Effects of hydrogen sulfide on AOX- and COX-mediated respiration capacities under various temperatures. AOX and COX capacities at indicated hydrogen sulfide concentrations are shown with fitted curves as indicated in the panel. Arrows indicate the points of intersection between the curves of the 2 oxidases. Assays were performed in triplicate and error bars display the mean ± standard deviation. **D)** Percentage of AOX capacities in total oxygen consumption by different terminal oxidases. The ratio of AOX respiration was calculated as the AOX respiration rate (*V*_(AOX)_) divided by the sum of the AOX and COX respiration rates (*V*_(AOX)_ + *V*_(COX)_). Assays were performed in triplicate and error bars display the mean ± standard deviation. In panels (**C**) and (**D**), quadratic curve fitting has been applied to the measurements. FW, fresh weight; SBP1/MTO, selenium-binding protein 1/methanethiol oxidase; AOX, alternative oxidase; COX, cytochrome *c* oxidase.

*SBP1/MTO* is specifically expressed within the florets of thermogenic spadices. We hypothesized that SBP1/MTO plays a role in the production of hydrogen sulfide, a byproduct catalyzed by MTO. To test this hypothesis, we compared hydrogen sulfide concentrations in both the florets and pith tissues from the thermogenic stage of spadices. Our data showed a marked elevation in hydrogen sulfide concentration within the florets in contrast to in the pith (Fig. 8A). The level of hydrogen sulfide in the thermogenic florets is higher than that in Arabidopsis leaves [approximately 30 nmol g^−1^ fresh weight (FW)] (Birke et al. 2015; Chen et al. 2020). In our western blot analysis, which targeted 2 distinct epitopes of SBP1/MTO in various samples from the thermogenic stage of *S. renifolius*, we observed a pronounced accumulation of SBP1/MTO proteins in the florets with both antibodies (Fig. 8B). Additionally, we detected a faint but detectable accumulation in the pith with the antibodies against the N-terminal epitope. In contrast, SBP1/MTO proteins were undetectable in both the leaf and spathe tissues with both antibodies. Finally, we confirmed the existence of SBP1/MTO in thermogenic florets through our protein purification and subsequent analysis using nanoscale liquid chromatography coupled to tandem mass spectrometry (nano-LC-MS/MS) (Supplementary Figs. S13 and S14).

Given the specialized role of florets in enhancing respiration during thermogenesis, and the considerable accumulation of hydrogen sulfide, a byproduct catalyzed by MTO, within these tissues, we decided to determine the effects of hydrogen sulfide on both AOX- and cytochrome *c* oxidase (COX)-mediated respiration capacities. To achieve this, we utilized purified mitochondria from thermogenic florets supplied with substrates specific to each respiratory pathway (Supplementary Fig. S15). Our respiration assays were carried out at 15, 23, and 30 °C, thus covering the full thermoregulatory range of this plant species (Seymour et al. 2010) (Fig. 8C). Notably, hydrogen sulfide displayed a more substantial inhibitory effect on COX-mediated respiration at all 3 temperatures examined. In contrast, AOX-mediated respiration showed relative insensitivity to the presence of hydrogen sulfide across the tested temperature range. Interestingly, under conditions of lower temperatures and higher concentrations of hydrogen sulfide in our experiments, mitochondrial respiration seemed to shift toward AOX-mediated respiration. Specifically, the intersection point between the 2 fitting curves for COX- and AOX-mediated respirations altered depending on the temperature, showing the lowest hydrogen sulfide concentration (2.96 µM) at 15 °C (Fig. 8C). In the presence of 10 µM hydrogen sulfide at 15 °C, the lowest temperature end of the thermoregulatory range (Seymour et al. 2010), mitochondrial respiration appeared to be exclusively dependent on AOX-mediated respiration (Fig. 8D). Assuming that the water content of the florets is about 90% and that water is evenly distributed across the tissue, and that our methylene blue method specifically detects the hydrogen sulfide (Li 2015), the average concentration of hydrogen sulfide in the cells of the thermogenic florets would be 90 µM. Although the actual concentrations in various cellular components, including mitochondria in the thermogenic florets, would require further investigation, our results fundamentally support the view that higher concentrations of hydrogen sulfide within the thermoregulatory temperature range promote AOX-mediated respiration in the thermogenic florets.

### Upregulation of genes associated with hydrogen peroxide scavenging in thermogenic florets

Considering that hydrogen peroxide is a product of MTO, alongside hydrogen sulfide (Supplementary Fig. S7B), we explored expression profiles of genes related to the scavenging of hydrogen peroxide by comparing the profiles between the florets and pith, and by investigating other possible genes that produce reactive oxygen species (Supplementary Fig. S16A). In the florets, we observed a higher expression of genes related to peroxidases, catalase, and superoxide dismutase as compared to the pith. As mentioned earlier, our analyses revealed that transcripts for *SBP1/MTO* were coexpressed with *AOX* (Supplementary Fig. S7A). AOX functions as a homodimer, and its reduced form is active, while the oxidized form is inactive (Umbach and Siedow 1993; Gelhaye et al. 2004; Vanlerberghe 2013; Selinski et al. 2018; Umekawa and Ito 2019). We thus further investigated the expression of genes related to glutathione metabolism, which is involved in the reduction of AOX proteins (Supplementary Fig. S16B). Our data suggested an upregulation in the thermogenic florets not only of *L-ascorbate peroxidase* (*APX*) but also of genes for other glutathione metabolism-related enzymes, including glutathione peroxidase, glutathione reductase, and glutathione hydrolase.

### One-carbon (C1) metabolism in thermogenic florets

Next, we focused on the C1 metabolism because formaldehyde is also a product of MTO activity (Supplementary Fig. S7B). Our transcriptome and metabolome analyses revealed a coordinated activation of the C1 metabolism, suggesting that the formaldehyde produced by SBP1/MTO is being efficiently reused (Supplementary Fig. S17). In particular, our transcriptome analysis indicated a significant increase in transcripts for formate dehydrogenase, categorized as Group F5, in the thermogenic florets (Fig. 3D and Supplementary Fig. S4). Formic acid is further utilized in the C1 metabolism, contributing to the synthesis of serine and deoxythymidine monophosphate, essential components of nucleotide metabolism, highlighting the interconnection of these metabolic pathways in thermogenic florets.

## Discussion

Our present study investigated thermogenesis in the skunk cabbage through an integrated approach combining transcriptome and metabolome analyses.

### Distinct roles of florets and pith in thermogenesis of the spadix

In exploring the transcriptome and metabolome of *S. renifolius*, we observed distinct patterns of gene expression and metabolite accumulation within both the florets and pith tissues across various thermogenic stages in the spadices. Our analyses revealed a notable upregulation of the carbohydrate metabolism and the mitochondrial electron transport pathway in the florets at the thermogenic stage of spadices, thereby suggesting their direct involvement in thermogenesis. In contrast, the gene expression and metabolic activities in the pith display markedly different profiles, indicating distinct roles from those in the thermogenic florets. Notably, the elevated gene expression for *heat shock 70 kDa proteins* in the pith during the thermogenic stage [Fig. 3E (Group P2) and Supplementary Fig. S4 (Group P2, Hot)] suggests that the heat generated in the florets imposes stress on the pith tissues. Additionally, in the pith of the prethermogenic spadices, we observed a high expression of *sucrose synthase 2* [Fig. 3D (Group P1b) and Supplementary Fig. S4 (Group P1b, Pre)]. In Arabidopsis, SUCROSE SYNTHASE 2 is shown to modulate metabolic homeostasis and to direct carbon toward starch synthesis in developing seeds (Angeles-Núñez and Tiessen 2010). Our findings suggest that the pith may indirectly contribute to thermogenesis by synthesizing sucrose, which could be partially transported to the florets and utilized as substrates during the thermogenic stage. Thus, our trans-omics analyses in the present study strongly suggest that the florets, rather than the pith tissue, are primarily responsible for thermogenesis in *S. renifolius*.

Our study also revealed specific gene expression of *Cys proteinase RD21A* in the pith during the postthermogenic stage (Fig. 3E (Group P3) and Supplementary Fig. S4). This finding provides a contrasting perspective to the earlier transcriptomic study (Ito-Inaba et al. 2012), which analyzed the whole spadix and postulated that Cys protease plays a role in the cessation of thermogenesis in the spadix. In our present study, however, we observed an increased Cys protease gene expression during the postthermogenic stage in the pith tissues. This implies that the roles of Cys protease, especially in the pith, might extend beyond simply contributing to the cessation of thermogenesis. Cys protease could also participate in other physiological processes, such as the recycling of endogenous nutrients, as previously reported (Liu et al. 2018).

### Convergence in gene expression and metabolite accumulation patterns in the thermogenic florets

**Figure 9.**
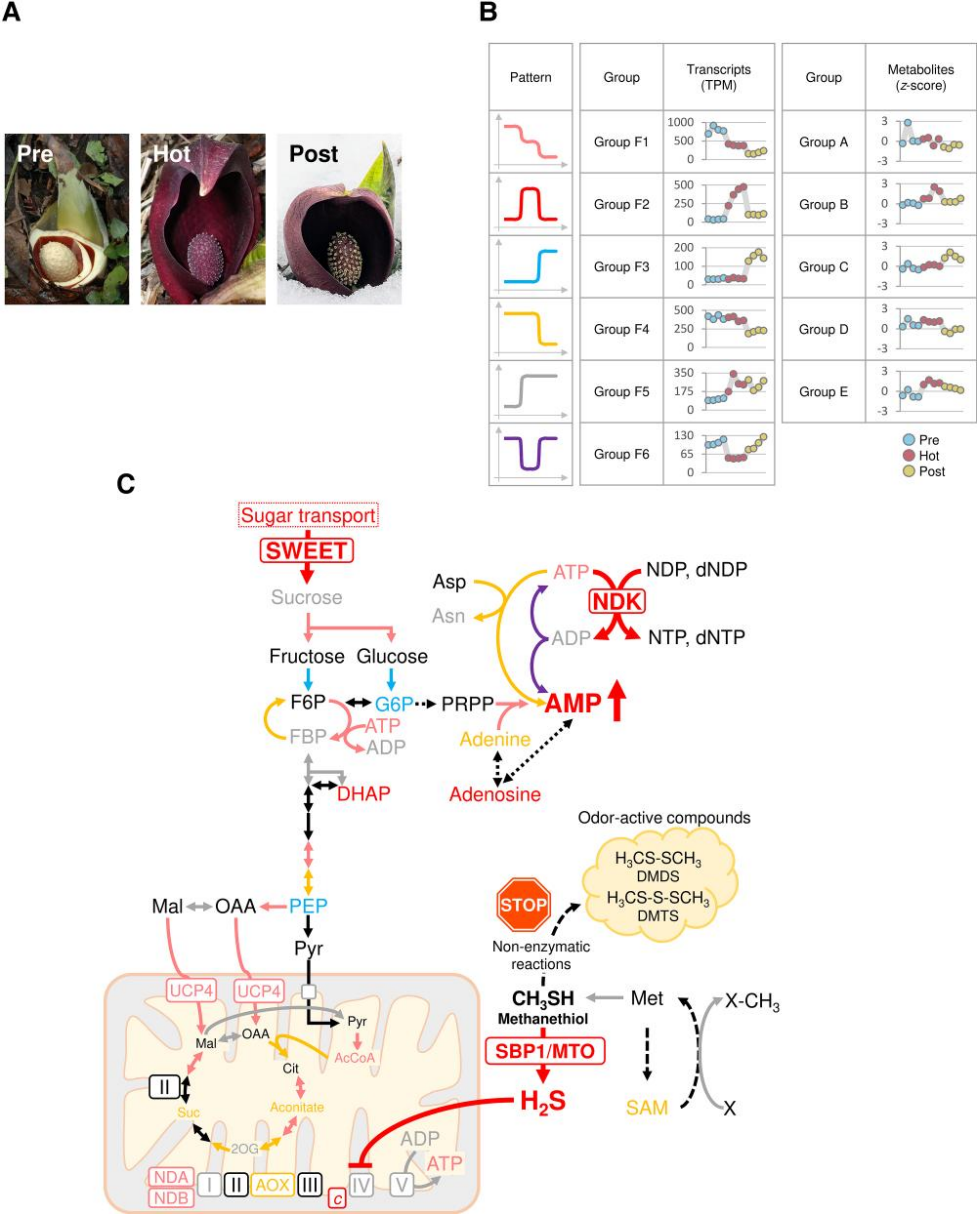
Convergence and unification of gene expression and metabolite accumulation patterns in the florets of the thermogenic spadices of *S. renifolius*. **A)** Representative images of *S. renifolius* at prethermogenic (Pre), thermogenic (Hot), and postthermogenic (Post) stages. In the prestage, the spathe is removed to reveal the spadix. **B)** Patterns of gene expression and metabolite accumulation in the florets across different stages. The symbols in the panel represent distinct gene expression/metabolite accumulation patterns in each group. The groups presented correspond to those for transcriptomic analysis (Fig. 3B) and for metabolomic analysis (Fig. 5F). **C)** Schematic overview of changes in gene expression and metabolic pathways during the development of the florets. Note that distinct patterns of gene expression during development largely merge maximally at the thermogenic stage, as shown by different symbols represented in the panel (**B**). Key shifts during the thermogenic stage include the upregulation of central carbon metabolism and mitochondrial respiratory chain components, including AOX. The SWEETs stimulate the glycolytic pathway. Increased ATP consumption is mediated by the NDK-driven nucleotide interconversion and the expression of *asparagine synthetase*. The elevation of AMP stimulates catabolic processes, thereby triggering a surge in metabolic heat-production. SBP1/MTO produces hydrogen sulfide by decomposing methanethiol, which represses COX activity and redirects metabolic flux toward the AOX respiratory pathway, contributing to metabolic heat-production. A shift toward methanethiol degradation and a reduction in the production of malodorous compounds (DMDS, DMTS) substantially diminishes the odor release from an intact spadix of *S. renifolius*. SBP1/MTO, selenium-binding protein 1/methanethiol oxidase; AOX, alternative oxidase; COX, cytochrome *c* oxidase; NDA, internal alternative NAD(P)H:ubiquinone oxidoreductase; NDB, external alternative NAD(P)H:ubiquinone oxidoreductase; NDK, nucleoside diphosphate kinase; SWEET, sugars will eventually be exported transporter; DMDS, dimethyl disulfide; DMTS, dimethyl trisulfide; AcCOA, acetyl-CoA; ADP, adenosine diphosphate; AMP, adenosine monophosphate; Asn, asparagine; Asp, aspartate; ATP, adenosine triphosphate; Cit, citrate; dNDP, deoxynucleoside diphosphate; dNTP, deoxynucleoside triphosphate; DHAP, dihydroxyacetone phosphate; F6P, fructose 6-phosphate; FBP, fructose 1,6-bisphosphate; G6P, glucose 6-phosphate; Mal, malate; NDP, nucleoside diphosphate; NTP, nucleoside triphosphate; OAA, oxaloacetate; PEP, phospho*enol*pyruvate; PRPP, 5-phosphoribosyl 1-pyrophosphate; SAM, *S*-adenosylmethionine; Suc, succinate; 2OG, 2-oxoglutarate.

Our analysis identified distinct groups of gene expression and metabolite accumulation patterns associated in a stage-specific thermogenesis manner in *S. renifolius*. These patterns demonstrate the dynamics of gene expression and metabolite accumulation throughout the developmental stages in the florets of this plant (Fig. 9). This intricate interplay includes a total of 6 gene expression patterns (Groups F1 to F6) and 5 metabolite accumulation patterns (Groups A to E); the changes in each component depend on the thermogenic status of this plant.

In these expression/accumulation profiles, certain genes and metabolites may be critical for initiating thermogenesis, while others may contribute to its maintenance, regulation, or even termination. For example, sugar transporters, including sugars will eventually be exported transporter (SWEET), may be involved in the initiation of thermogenesis by enhancing the glycolytic pathway in the florets as previously reported in the carpels of lotus (Zheng et al. 2022). During the thermogenic stage, AOX acts as a component of cyanide-insensitive mitochondrial respiration, utilizing a steady and sufficient supply of reduced equivalents for its function. Notably, even in the prethermogenic stage, the level of *AOX* transcripts is already high, although determining the protein accumulation remains necessary, suggesting its alternative role in responding to cold rather than for thermogenesis, as seen in other nonthermogenic plants (Umbach et al. 2009; Heidarvand and Maali Amiri 2010; Wang et al. 2011; Wang et al. 2012). Similarly, the expression levels of genes for alternative dehydrogenases (NDA and NDB) are heightened in the florets during the prethermogenic stage of spadices, suggesting a role for NDA and NDB in enhancing tolerance to the environmental stresses encountered where *S. renifolius* grows (Sweetman et al. 2019; Jethva et al. 2023).

Thus, genes and metabolites in each group (Fig. 9) play a role at each developmental stage, and some of these may have roles apart from thermogenesis. However, a systematic convergence or merging in the expression of genes and accumulation of metabolites occurs exclusively at the female stage, where stage-specific thermogenesis takes place. This convergence, which may seem coincidental but is presumably inherent and programmed to this plant, serves as a crucial mechanism for controlling developmentally regulated thermogenesis in *S. renifolius*.

### The role of SBP1/MTO in hydrogen sulfide accumulation, AOX, and thermogenesis

One of the important findings from our present study was the identification of the gene encoding SBP1/MTO, which emerged as the most highly expressed transcript in the thermogenic spadix of *S. renifolius*. This unexpected finding is important for our understanding of the physiological aspects of thermogenesis in this plant.

SBP1 was initially recognized for its capacity to bind with the radioactive isotope of selenium (Bansal et al. 1989; Elhodaky and Diamond 2018). However, recent studies have revealed that SBP1 in bacteria, humans and nematode functions as an MTO (Eyice et al. 2018; Pol et al. 2018; Philipp et al. 2022). Crucially, this enzyme is capable of converting methanethiol, a precursor for malodorous sulfur-containing organic compounds such as DMS, into a less volatile form, hydrogen sulfide. A genetic deficiency in human SBP1, in fact, impairs this conversion process, leading to conditions like extra-oral halitosis (Pol et al. 2018).

We observed a higher accumulation of hydrogen sulfide in the thermogenic florets in this study (Fig. 8A), and the product of *SrSBP1/MTO* isolated from *S. renifolius* exhibited conserved amino acids that are essential for MTO activities (Supplementary Fig. S12). This suggested that the elevated accumulation of hydrogen sulfide in thermogenic florets arises from the active functioning of SBP1/MTO in this tissue. Importantly, this provides a rational explanation for the lack of odor in *S. renifolius*, recalling a historical report on the smells of *S. foetidus*: “an uninjured skunk cabbage flower has a faintly sweetish smell that gives no hint of the mephitic odor produced by any damaged part of the plant—a scent that one observer described as a mixture of skunk, putrid meat, and garlic” (Knutson 1979).

Hydrogen sulfide inhibits respiration by repressing the activity of COX (Martin and Maricle 2015), and overexpression of *SBP1* has been observed to repress the mitochondrial respiration in prostate cancer cells (Elhodaky et al. 2020). It has also been shown that SBP1 acts as an endogenous stimulator of adipocyte differentiation by synthesizing hydrogen sulfide (Randi et al. 2021). In plants and marine invertebrates, sulfide treatment has been shown to upregulate *AOX* and enhance cyanide-tolerant alternative respiratory pathways (Bremer et al. 2021; Fang et al. 2022; Zhong et al. 2023). Based on our current findings for the temperature sensitivities against hydrogen sulfide (Fig. 8C and D), we propose a scenario for the thermoregulation of *S. renifolius*. Namely, the hydrogen sulfide generated through the enzymatic activity of SBP1/MTO modulates the balance between the 2 primary mitochondrial respiration pathways. Specifically, under conditions of elevated hydrogen sulfide concentration, a decrease in temperature leads to the suppression of COX-mediated mitochondrial respiration, and AOX-mediated respiration may become dominant, further promoting a metabolic pathway toward enhanced thermogenesis. Although the respiration analysis utilizing isolated mitochondria may not fully represent the actual cellular conditions within the florets of thermogenic spadices, the findings of this study provide insights into the mechanism of thermoregulation in *S. renifolius*. Specifically, observations regarding the intersection point of AOX- and COX-mediated respiration under varying concentrations of hydrogen sulfide (Fig. 8C) demonstrate a dynamic interplay among temperature, hydrogen sulfide levels, and mitochondrial respiration. In our previous study, which employed thermodynamic analysis on intact spadices of *S. renifolius*, we demonstrated that thermoregulation in this species is governed by a negative activation energy that involves biochemical pre-equilibrium reactions, including a rate-determining reaction catalyzed by the mitochondrial terminal oxidases AOX and COX (Umekawa et al. 2016). Therefore, changes in the equilibrium point of the biochemical reactions for thermogenesis, which utilize already established gene expression levels, are pivotal in thermoregulation. Thus, our current study further suggests that hydrogen sulfide, the product of SBP1/MTO, can act as an effector in altering the biochemical equilibrium point, thereby controlling cellular respiration and contributing to thermoregulation in the spadices of *S. renifolius*.

Accordingly, our finding of the extremely high level of *SBP1/MTO* transcripts may shed light on fundamental questions surrounding *S. renifolius*: why the thermogenic spadix does not emit a strong odor, contrary to its English name, skunk cabbage, and how this plant can regulate its spadix temperature under fluctuating ambient conditions. Nevertheless, a more extensive investigation is warranted to thoroughly understand the physiological role, including the temperature dependency of its activities, of SBP1/MTO in *S. renifolius*.

### Association between nucleotide levels and thermogenesis

In addition to these findings, our metabolome analysis demonstrated an elevation in nucleotide metabolism, including the turnover of ATP. Specifically, our study showed an increase in AMP levels in thermogenic florets (Fig. 6A). Because AMP has been shown to indicate a low-energy status within the cell (Baena-González et al. 2007), a thermogenic specific increase in AMP may act as a signal to stimulate a set of metabolic pathways for ATP homeostasis. This, in turn, activates additional metabolic pathways for exergonic reactions, ultimately linked to thermogenesis. This increase in AMP is likely driven by various enzymes, such as Asn synthetase, that utilize ATP as an energy source, consequently producing AMP (Figs. 6B and 9C). Furthermore, the increased capacities of the mitochondrial alternative respiration pathway in the florets of thermogenic spadices, as previously reported by Onda et al. (2007) could decrease the ATP/ADP ratio; this change may, in turn, result in an increased level of AMP through the upregulated gene expression of *ADK* (Fig. 6B). NDK, an enzyme involved in the interconversion between NTP and NDP, also contribute to this process. Interestingly, a previous report on the thermoregulatory plant *D. vulgaris* has shown that the level of cellular AMP rises in association with increased respiration in thermogenic tissue (Ito et al. 2013). Although AMP functions as an allosteric activator for PFK in mammals, the regulation of plant PFK and glycolysis is not substantially influenced by adenylates, including AMP (Plaxton 1996; Traut 2008; Sola-Penna et al. 2010; Brüser et al. 2012). Therefore, further studies are required to clarify the precise effects and detailed mechanisms of increased AMP on thermogenesis in *S. renifolius*.

### Sulfur and carbon recycling for thermogenesis

Given that methanethiol is catabolized by MTO to form hydrogen sulfide, which can then be utilized in C1 metabolism (Supplementary Fig. S17), sulfur is incorporated into Cys (Fig. 7C), a pivotal precursor for the majority of sulfur-containing metabolites (Takahashi et al. 2014). Intriguingly, our analysis demonstrates that accumulation of Cys in thermogenic florets is highest during developmental stages (Fig. 5D and Supplementary Table S2). In the C1 metabolism, carbon is ultimately converted into CO_2_, which, in the form of bicarbonate, is catabolized to oxaloacetate by PEPC, and it furnishes substrates for the TCA cycle (Sayed et al. 2016). Thus, our findings further suggest a coordinated recycling mechanism that contributes to energy-efficient thermogenesis in this plant.

### Convergence and the existence of largely unknown genes in *S. renifolius*

Convergence, the process wherein multiple biological functions merge to achieve a specific outcome, is pivotal to biological complexity and adaptability (Meinzer 2003; Baena-González and Sheen 2008). Our present study further suggests that the convergence of different gene and metabolite groups, rather than singular “thermogenic genes”, is responsible for developmentally controlled thermogenesis in *S. renifolius*. This evolutionarily acquired strategy confers the adaptability, resilience, and robustness necessary for survival in the cold environments where it blooms.

It should also be noted that in the present study, among the 63,705 sequences longer than 1,000 base pairs, approximately three-fourths did not show homology to Arabidopsis genes. This lack of homology further suggests the existence of a large proportion of currently unknown genes, some of which could extend or enrich our understanding of the proposed convergence mechanism for thermogenesis. Therefore, investigating these unknown genes may lead to the discovery of additional mechanisms responsible for developmentally controlled thermogenesis in *S. renifolius*.

### Concluding remarks

Our integrative approach, which combines transcriptomic and metabolomic analyses, substantially advances our understanding of thermogenesis in *S. renifolius*. Our collective findings highlight a convergence of gene expression and metabolite accumulation patterns during thermogenesis and, importantly, the high expression of *SBP1/MTO*, along with the potential function of SBP1/MTO, in *S. renifolius*. Our study also lays a solid foundation for future investigations into the molecular mechanisms underlying thermoregulation in *S. renifolius*.

## Materials and methods

### Plant materials

A total of 4 spadices at the prethermogenic, thermogenic, and postthermogenic stages of skunk cabbage (*S. renifolius*) were collected for concurrent transcriptome and metabolome analyses. The sampling was conducted from late March to early April in 2015 from their natural habitats situated in Fujine (39°18′52″N, 141°02′02″E) and Kanegasaki (39°14′18″N, 141°02′58″E), within the Iwate Prefecture of northern Honshu, Japan. Upon sampling, the florets and pith were promptly excised from the spadices using a sharp cutter blade and then immediately flash-frozen in liquid nitrogen. The frozen tissues were subsequently ground into a fine powder with a pestle and mortar under a continuous flow of liquid nitrogen for subsequent experimental analyses.

### Temperature measurements

Both the spadix and ambient temperatures at the study sites were measured using a portable digital thermometer (TR-52, T&D Corporation, Nagano, Japan). The sensor probe was inserted with minimal tissue disruption, as previously described (Ito et al. 2004; Onda et al. 2008). Thermal images of the intact *S. renifolius* were captured using an infrared camera (Teledyne FLIR LLC, Wilsonville, OR, USA).

### Normalized cDNA synthesis and de novo assembly

Total RNA isolation from thermogenic florets was performed using an RNeasy Plant Mini Kit (Qiagen, Hilden, Germany) in accordance with the manufacturer’s instructions. From the extracted total RNA, 43.2 μg was used to synthesize the normalized cDNA and construct a cDNA library (Eurofins Genomics, Tokyo, Japan). The generated cDNA was sequenced using the MiSeq platform and subsequently de novo assembled through Eurofins’ proprietary pipeline, which employs a multi-k-mer approach powered by the Velvet and Oases software packages.

### Functional annotation of transcripts

Homology BLAST searches were executed against both the UniProtKB reviewed FASTA dataset (Swiss-Prot; https://www.uniprot.org/help/downloads) and the translated CDS from the Araport11 genome release (https://www.arabidopsis.org/index.jsp) to identify the entries most similar to each gene. Sequences with e-values less than 10^−5^ were considered known genes and subjected to further analysis. This resulted in a set of 15,904 nonduplicate sequences that were used for the quantitative transcriptome analysis using RNA-seq. GO annotation was performed based on the Arabidopsis (*A. thaliana*) accession number from the Araport11 genome release.

### Quantitative RNA-seq analysis

For the quantitative RNA-seq analysis, total RNA was extracted from up to 100 mg of the frozen tissue powder using the RNeasy Plant Mini Kit, following the manufacturer’s instructions. This isolated RNA was subjected to single-end read sequencing on a HiSeq3000 instrument (Illumina Inc., San Diego, CA, USA). The resulting Illumina sequencing reads were then mapped to the reference sequencing library consisting of 15,904 transcripts using Strand NGS software (www.strand-ngs.com). The mapping was conducted in accordance with the manufacturer’s defaults, except to allow counting of reads with homology to multiple sequences. Raw counts for each sample were normalized using the transcripts per million (TPM) method. We further categorized genes as “expressed” if they exhibited a TPM value greater than 1 in at least 1 out of 3 or 4 samples from the same sampling category: prethermogenic (Pre), thermogenic (Hot), and postthermogenic (Post) stages of spadices.

### Differential gene expression analysis

Differentially expressed genes (DEGs) were identified through an organwise comparison between the florets and the pith of the spadix at various thermogenic stages. We conducted a quantitative analysis using 4 biological replicates, except for the prethermogenic stage pith samples, where only 3 replicates were used. The Strand NGS statistical replicate analysis tool was employed for statistical analysis, operating under Benjamini–Hochberg-corrected moderated *t*-test conditions. This approach adjusts for small sample sizes to improve the estimation of gene variances (Benjamini and Hochberg 1995; Smyth 2004). We considered gene expression significant if the *P*-value was less than 0.05 and the absolute fold change exceeded 2.0. Unless otherwise stated, all parameters adhered to the software’s default settings. For the identified DEGs, we counted the number of gene overlaps between thermogenic stages in both florets and pith, and subsequently drew a Venn diagram. The gene expression level data obtained were then imported into the R software for both hierarchical and nonhierarchical (k-means) clustering. Before clustering analysis, TPM values were converted to *z*-scores for each gene by the “genescale” function included in the “genefilter” package obtained from bioconductor.org. Hierarchical clustering analysis and drawing of heatmaps with dendrograms were performed using the heatmap.2 function of the “gplots” package. Nonhierarchical clustering was performed with the k-means function in the base package of R using *z*-score normalized data as well as hierarchical clustering.

### GO enrichment analysis

Functional GO annotation of *S. renifolius* genes was performed based on sequence homology with CDS of Arabidopsis. The latest version of the GO list (ATH_GO_GOSLIM.txt) was downloaded from Araport11. GO enrichment analysis was conducted using the Strand NGS software suite, in conjunction with RNA-Seq. For this analysis, the criteria for the enrichment of GO terms were set to a *P*-value less than 0.05. To reduce redundancy in similar GO terms, the enriched GO terms were summarized using REVIGO (http://revigo.irb.hr/), and subsequently sorted in descending order by their respective *P*-value. During this summarization, a “small” result size setting (0.5) was employed, and the database was selectively refined to include only terms applicable to Arabidopsis.

### Metabolite analysis by capillary electrophoresis-mass spectrometry

Metabolite extraction and measurements were performed according to previously described methods (Takahashi et al. 2012; Ito et al. 2013). Briefly, metabolites were extracted from snap frozen tissue with ice-cold 50% (v/v) methanol containing 50 μM methionine sulfone and 1,4-piperazinediethanesulfonic acid as internal standards, for 5 min at 4 °C. After the extract was centrifuged at 15,000*×g* for 5 min, the supernatant passed through an ultrafiltration column with a cutoff of 5 kDa (Amincon, Millipore, Billerica, MA, USA) to obtain a filtrate. The separation and quantification of the metabolites were performed using the capillary electrophoresis system (1100 series, Agilent Technologies, Santa Clara, CA, USA), with uncoated fused-silica capillaries (GL Sciences, Torrance, CA, USA) and 1 M formic acid running buffer (pH 1.9) for cation assays and with polyethylene glycol-coated capillaries (DB-WAX, J&W Scientific, Folsom, CA, USA) and 20 mM ammonium acetate (pH 9.0) for anion assays. The quantification of metabolites was executed using ChemStation software (Agilent Technologies).

### Analysis of metabolome data

Metabolome data analysis was performed with the web-based version of MetaboAnalyst 5.0 (https://www.metaboanalyst.ca/MetaboAnalyst/). Metabolome quantification data were formatted with Microsoft Excel, and data scaling was normalized by specifying auto-scaling. PCA was performed on the normalized data using MetaboAnalyst, and the loadings were calculated in Excel from the MetaboAnalyst output. For the identification of upregulated metabolites, we performed *t*-tests under conditions where significant absolute fold changes were > 2 and false discovery rate adjusted *P*-values were < 0.05. Hierarchical clustering and k-means clustering were performed on data normalized to *z*-score using the same methods as the gene expression analysis described above. To estimate the energy status of cells, adenylate energy charge was calculated by using the following formula: (ATP + ½ ADP)/(ATP + ADP + AMP).

### Reverse transcription quantitative PCR

Transcript expression levels were analyzed using RT-qPCR. Total RNA was extracted with the RNeasy Plant Mini Kit, and cDNA synthesis was performed using the ReverTra Ace qPCR RT Master Mix and gDNA Remover (TOYOBO, Osaka, Japan). qPCR was performed on a Dice Thermal Cycler (TP-800, Takara Bio, Otsu, Japan) using the THUNDERBIRD SYBR qPCR Mix (TOYOBO) and the primers listed in Supplementary Table S5.

### Alignment analysis

Amino acid sequences of MTO or SBP1 were obtained from GenBank (https://www.ncbi.nlm.nih.gov/genbank/). The downloaded fasta files were imported into Geneious Prime 2019 (https://www.geneious.com/) and aligned using MUSCLE.

### Determination of endogenous hydrogen sulfide and selenium

The concentration of hydrogen sulfide in each tissue of *S. renifolius* during the thermogenic stage was measured by the methylene blue method (Li 2015). In brief, 0.2 g of frozen tissue powder was suspended in extraction buffer (20 mM Tris-HCl, 10 mM EDTA, 20 mM zinc acetate, pH 8.0). The supernatant containing the sulfide was then treated with FeCl_3_ reagent (30 mM FeCl_3_ dissolved in 1.2 M HCl) and DMPD reagent [20 mM *N*,*N*-dimethyl-*p*-phenylenediamine dihydrochloride (DMPD) dissolved in 7.2 M HCl]. The resulting methylene blue coloration was measured at an absorbance of 667 nm. The calibration curves were generated using sodium sulfide nonahydrate as a reference. For Se determination, approximately 0.05 g of frozen tissue powder was treated with 35% (v/v) nitric acid and subjected to microwave-assisted digestion. The Se content was determined by inductively coupled plasma mass spectrometry (ICP-MS; 7700x, Agilent Technologies), with ^78^Se monitored and with ^128^Te used as an internal standard.

### Total protein extraction and western blotting

Protein extraction was initiated by suspending powdered, frozen tissue in a specialized extraction buffer, composed of 50 mM Tris-HCl, 150 mM NaCl, 1% (v/v) Triton X-100, 0.5% (w/v) sodium deoxycholate, 1% (w/v) SDS, 1 mM EDTA, 28 μM E-64, and cOmplete ULTRA, Mini EDTA-free tablets (Roche Diagnostics, Mannheim, Germany). The homogenates were subsequently subjected to centrifugation at 20,000*×g*, 4 °C. The protein content in the collected supernatant was then quantified with the Pierce BCA Protein Assay Kit (Thermo Fisher Scientific, Waltham, MA, USA) as per the manufacturer’s instructions. For western blotting analysis, aliquots of the protein samples (each containing 20 μg of total protein) were resolved on 9% (w/v) acrylamide gels, and transferred onto a polyvinylidene difluoride (PVDF) membrane (ImmobilonR-P PVDF Membrane, Millipore) (Ito et al. 2020). Anti-SBP1/MTO primary antibodies were generated though the immunization of rabbits with 2 distinct peptides, HGAKGPGYASPLEA-Cys and Cys-SAWDNQFYPELKQKG, corresponding to the N-terminus and C-terminus of SBP1/MTO, respectively. The primary antibody was used at a dilution of 1:50,000. As a secondary antibody, HRP-conjugated anti-Rabbit IgG antibody (Invitrogen, Carlsbad, CA, USA) was used at a dilution of 1:2,000. Signal detection for the target protein was performed using a chemiluminescence-based technique, by the Amersham ECL start Western Blotting Detection Reagent (Cytiva, Tokyo, Japan).

### Purification of mitochondria and respiratory capacity measurements

Mitochondria were purified from the florets at the thermogenic stage of the spadices using previously described methods with minor modifications (Umekawa et al. 2016; Umekawa and Ito 2019). These modifications included adjustments to the composition of the grinding medium, wash medium, and Percoll-containing buffer. The grinding medium consisted of 0.3 M mannitol, 20 mM MOPS-KOH, 2 mM EDTA, 2 mM pyruvate, 0.2% (w/v) BSA, 7 mM Cys, and EDTA-free cOmplete ULTRA tablets (Roche), pH 7.5. The wash medium contained 0.3 M mannitol, 20 mM MOPS-KOH, 2 mM EDTA, 2 mM pyruvate, and 0.2% (w/v) BSA, pH 7.5. The Percoll-containing buffer comprised 21% (v/v) Percoll, 0.3 M sucrose, 20 mM MOPS-KOH, 5 mM KH_2_PO_4_, 10 mM KCl, 1 mM MgCl_2_, and 0.1% (w/v) BSA, pH 7.5. Isolated mitochondria were aliquoted and stored at −80 °C. Respiration of frozen-thawed mitochondria was measured in respiration buffer (0.3 M sucrose, 20 mM MOPS-KOH, 5 mM KH_2_PO_4_, 10 mM KCl, 1 mM MgCl_2_, 0.1% (w/v) BSA, pH 7.5) with a Clark-type polarographic oxygen electrode (Oxy1, Hansatech Instruments, King's Lynn, UK). Oxygen consumption via COX capacity was determined by the addition of 2 mM ascorbate, 0.5 mM *N*, *N*, *N”*, *N′*-tetramethyl-*p*-phenylenediamine and 0.006% (w/v) horse myocardium cytochrome *c*, with AOX inhibited by 0.1 mM *n*-propyl gallate. AOX capacity was measured in the presence of 1 mM durohydroquinoane, 10 mM pyruvate and 0.5 mM KCN. To evaluate the oxygen consumption rates in the presence of hydrogen sulfide, a freshly prepared sodium sulfide nonahydrate stock solution was added in each respiration assay.

### Purification of SBP1/MTO protein

The cytoplasmic fraction was recovered from the supernatant obtained during the mitochondrial isolation process described above. Before the purification process, the crude extracts were centrifuged at 20,000*×g* for 15 min at 4 °C to remove any precipitates. After centrifugation, the supernatant was subjected to ammonium sulfate fractionation. Proteins precipitated with 40% to 60% saturated ammonium sulfate were pelleted by centrifugation at 20,000*×g* for 10 min at 4 °C. The pellet was washed twice with 60% saturated ammonium sulfate and dissolved in Tris-buffer (50 mM Tris-HCl, 150 mM NaCl, pH 7.6). Then, fractions containing SBP1 were exchanged with start buffer (50 mM Tris-HCl, 1 M ammonium sulfate, pH 7.6) using a PD-10 column (Cytiva) and applied to a HiTrap Phenyl HP column (Cytiva) equilibrated with the start buffer. A decreasing linear ammonium sulfate gradient from 1 M to 0 M over 10 column volumes (cv) was used for protein elution. All buffers used for purification experiments contained EDTA-free cOmplete ULTRA tablets (Roche).

### Purification of SBP1/MTO by antibody-immobilized column

An immunoaffinity chromatography column (IAC) was prepared by immobilizing 1 mg of anti-SBP1N antibody on a HiTrap NHS-activated HP Column (Cytiva) according to the manufacturer’s instructions. Following the further purification by the HiTrap Phenyl HP column, the buffer containing the target protein was then exchanged into the binding buffer (50 mM Tris-HCl, 150 mM NaCl, pH 7.6), and subsequently applied to the antibody-coupled column. Antibody-antigen reactions were carried out for 60 min at 25 °C, followed by the removal of unbound proteins with 10 cv of binding buffer. Proteins bound to immobilized antibodies were eluted with MCE buffer [3 M MgCl_2_, 75 mM HEPES-NaOH, 25% ethylene glycol (v/v), pH 7.1] (Ben-David and Firer 1996).

### Protein identification by nano-LC-MS/MS

Proteins obtained by IAC were electrophoresed on a 9% polyacrylamide gel. Coomassie Brilliant Blue (CBB)-stained bands were excised with a scalpel and in-gel digested with Trypsin/Lys-C Mix, Mass Spec Grade (Promega, Madison, WI, USA), and ProteaseMAX Surfactant, Trypsin Enhancer (Promega), according to the manufacturer’s instructions. Nano-LC-MS/MS was performed using the LTQ Orbitrap XL system (Thermo Fisher Scientific) by the method previously described (Ito et al. 2011; Kakizaki et al. 2012). Mass spectra were submitted to MASCOT Server 2.3.02 (Matrix Science, Tokyo, Japan) and searched against the SWISS-PROT database, as well as a known *S. renifolius* amino acid sequence database containing 142 entries.

### Data transformation and statistical analysis

Standard data processing and statistical analyses were conducted using Microsoft Excel and R (version 4.2.2). R was utilized for various analyses, including hierarchical clustering, heatmap generation, and k-means clustering. Additionally, *t*-tests, Tukey’s Honest Significant Difference (HSD) tests with the “multcomp” package, and Steel-Dwass tests with the “NSM3” package were performed in R. For data analysis, the “tidyr” and “dplyr” packages in R were used. The PCA with the transcriptome data was performed using the “prcomp” function, and the graphs were plotted using “ggplot2”.

### Accession numbers

The Illumina read sequencing data were deposited in DDBJ Sequence Read Archive with accession numbers DRA005977 and DRA016902. The CDS sequences from *S. renifolius* are available in DDBJ (https://www.ddbj.nig.ac.jp/index-e.html). The accession numbers of the sequences are as follows: *SrSBP1* (LC717500), *SrROMT* (LC775304), *SrAOX* (AB183695), *SrEF1α* (AB677835), *SrPFPβ* (LC775303), *SrPFK* (LC775301), and *SrFBPase* (LC775302).

## Supplementary Material

kiae059_Supplementary_Data

## Supplementary data

The following materials are available in the online version of this article.

**Supplementary Figure S1**. Flowchart of RNA-seq and metabolome analyses in this study.

**Supplementary Figure S2**. Workflow of RNA-Seq data analysis for differentially expressed genes (DEGs) in this study.

**Supplementary Figure S3**. Principal component analysis (PCA) based on expression patterns of differentially expressed genes (DEGs) in the florets and pith at various thermogenic stages of the spadices of *S. renifolius*.

**Supplementary Figure S4**. Comprehensive gene expression profiling across various thermogenic stages in *S. renifolius*.

**Supplementary Figure S5**. Enriched Gene Ontology (GO) terms identified by k-means clustering in the florets and pith of *S. renifolius*.

**Supplementary Figure S6**. Contribution of differentially expressed genes (DEGs) to tissue-specific and developmentally controlled thermogenesis in the spadix of *S. renifolius*.

**Supplementary Figure S7**. Verification of gene expression in various tissues in the thermogenic stage of *S. renifolius*.

**Supplementary Figure S8**. Differential metabolite profiling across various thermogenic stages in *S. renifolius*.

**Supplementary Figure S9**. Tissue-specific expression of genes involved in the ATP-consuming futile cycle at the thermogenic stage of *S. renifolius*.

**Supplementary Figure S10**. Metabolic pathways involved in the de novo synthesis of purine and pyrimidine nucleotides in the spadices of *S. renifolius*.

**Supplementary Figure S11**. Nucleotide interconversions and accumulation levels of metabolites.

**Supplementary Figure S12**. Comparative alignment of deduced methanethiol oxidase (MTO) and selenium-binding protein 1 (SBP1) amino acid sequences.

**Supplementary Figure S13**. Purification of selenium-binding protein 1/methanethiol oxidase (SBP1/MTO) protein.

**Supplementary Figure S14**. Identification of *S. renifolius* selenium-binding protein 1/methanethiol oxidase (SBP1/MTO) by nanoscale liquid chromatography coupled to tandem mass spectrometry (nano LC-MS/MS).

**Supplementary Figure S15**. Experimental procedures for measuring respiration capacities using purified mitochondria from thermogenic florets of *S. renifolius*.

**Supplementary Figure S16**. Reactive oxygen species (ROS) dynamics, glutathione metabolism, and gene expression levels in the thermogenic florets of *S. renifolius*.

**Supplementary Figure S17**. Metabolic pathway of one-carbon compounds in the thermogenic florets of *S. renifolius*.

**Supplementary Table S1**. Thermogenic inflorescences of *S. renifolius*.

**Supplementary Table S2**. Concentrations of metabolites in the florets and pith across different thermogenic stages in *S. renifolius*.

**Supplementary Table S3**. Tissue- and thermogenic stage-specific statistical analysis of metabolite accumulation in *S. renifolius*.

**Supplementary Table S4**. Selenium contents in various organs of *S. renifolius*.

**Supplementary Table S5**. Sequences of the primers used for RT-qPCR.

**Supplementary Data Set 1**. Transcripts per million (TPM) normalized expression values of genes identified in *S. renifolius*.

**Supplementary Data Set 2**. Differentially expressed genes (DEGs) between florets and pith at various thermogenic stages in *S. renifolius*.

**Supplementary Data Set 3**. Catalogue of genes constituting the Venn diagram of differentially expressed genes (DEGs).

**Supplementary Data Set 4**. Principal component analysis (PCA) loadings of differentially expressed genes (DEGs) between the florets and pith tissues at various thermogenic stages in the spadices of *S. renifolius*.

**Supplementary Data Set 5**. Catalog of differentially expressed genes (DEGs) grouped by k-means clustering in the florets and pith tissues at various thermogenic stages of spadices in *S. renifolius*.

**Supplementary Data Set 6**. Detailed information as to principal component analysis (PCA) loading values of differentially expressed genes (DEGs) categorized by various groups for florets and pith tissues in *S. renifolius*.

**Supplementary Data Set 7**. Metabolite concentrations across various thermogenic stages in *S. renifolius*.

## Dive Curated Terms

The following phenotypic, genotypic, and functional terms are of significance to the work described in this paper:

ATP CHEBI: CHEBI:15422

PGM Gramene: AT1G78050
PGM Araport: AT1G78050

UCP1 Gramene: AT3G54110
UCP1 Araport: AT3G54110

JMT Gramene: AT1G19640
JMT Araport: AT1G19640

DCD Gramene: AT1G48420
DCD Araport: AT1G48420

LCD Gramene: AT3G62130
LCD Araport: AT3G62130
