# Supplementary material for: Gene expression and metabolite levels converge in the thermogenic spadix of skunk cabbage
Source: Plant Physiol. 2024 Feb 6;195(2):1561–85. doi: 10.1093/plphys/kiae059 (PMC11142342; doi:10.1093/plphys/kiae059)
Supplement: kiae059_Supplementary_Data [file kiae059_supplementary_data.zip › Tanimoto et al_Supplemental_Figures_S1_S17_Supplemental_Tables S1_S5.pdf]

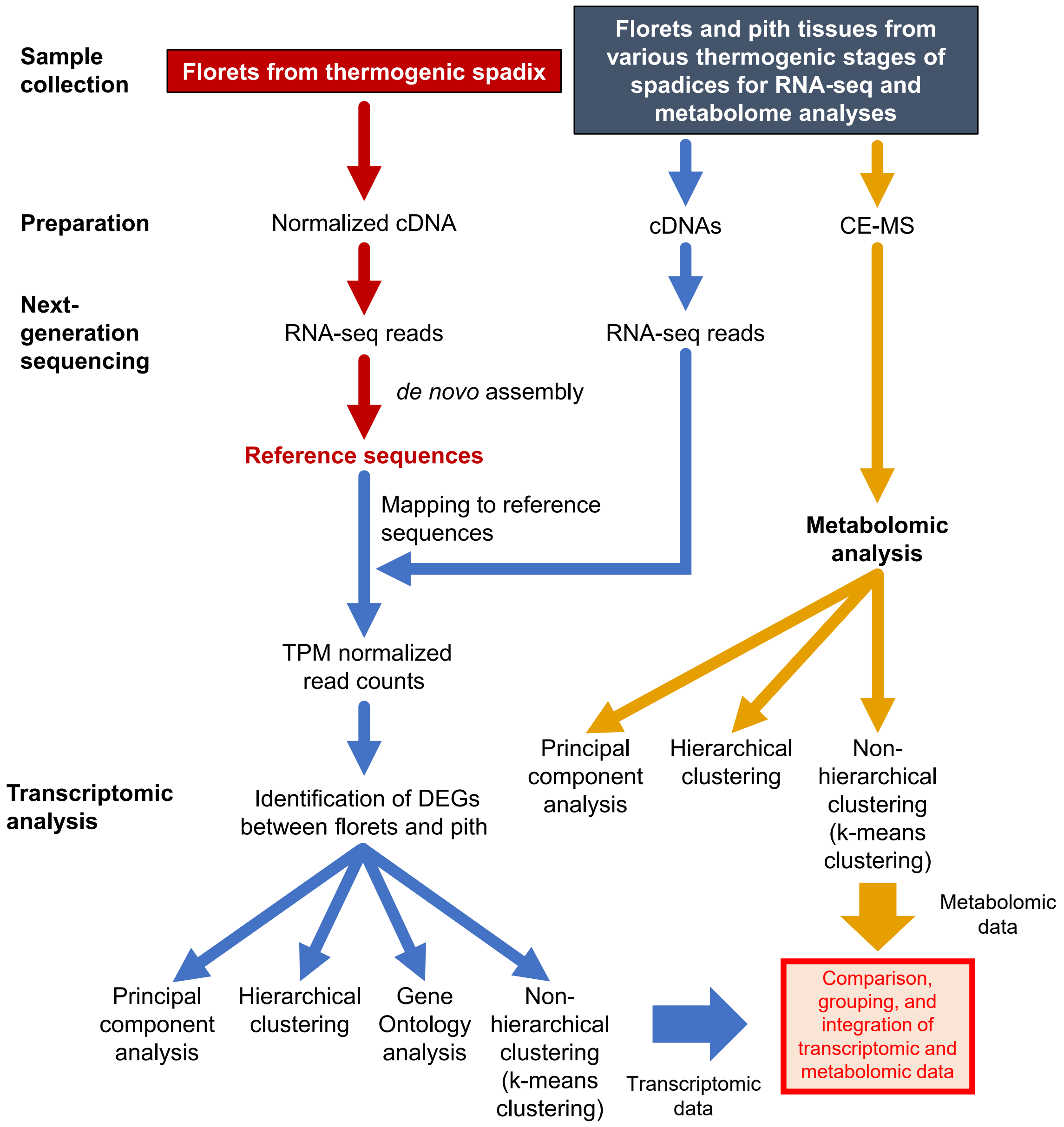

Supplemental Figure S1. Flowchart of RNA-seq and metabolome analyses in this study.
Red arrows denote the construction of normalized cDNA and *de novo* transcriptome assembly for creating reference sequences. Blue arrows indicate RNA-seq and transcriptome analyses across thermogenic stages. Orange arrows detail the metabolome analysis via capillary electrophoresis-mass spectrometry (CE-MS).

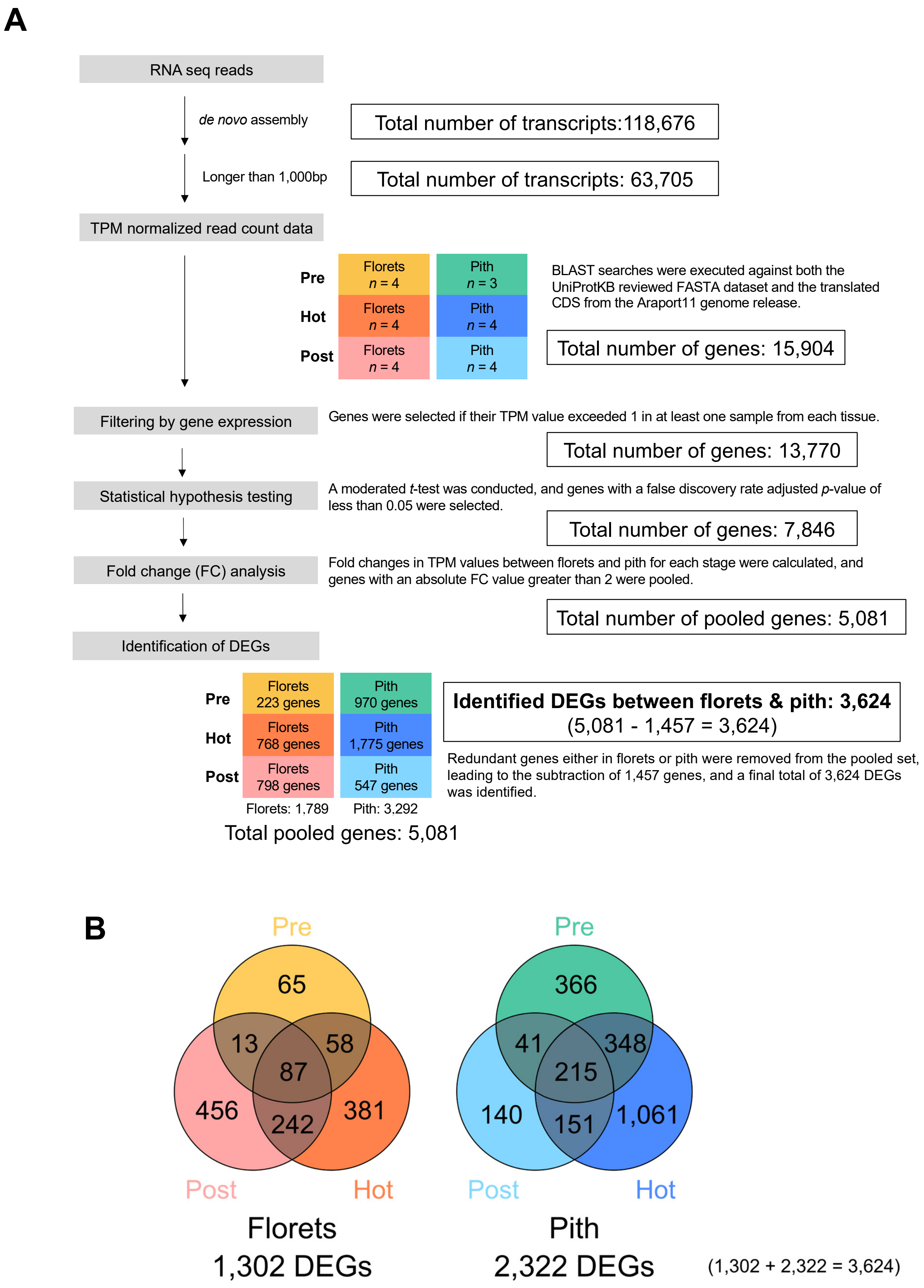

Supplemental Figure S2. Workflow of RNA-Seq data analysis for differentially expressed genes (DEGs) in this study.
(A) The collected TPM normalized read count data were initially filtered based on gene expression levels, followed by statistical analyses including a moderated *t*-test, which adjusts for small sample sizes to improve the estimation of gene variances (Smyth, 2004). Subsequent fold change (FC) analysis identified DEGs between florets and pith. The number of genes at each step, as well as the total number of identified DEGs, is also given. (B) The Venn diagrams display the shared genes and those distinctive to each thermogenic stage, identified as DEGs among the different stages (Pre, Post, and Hot) for both florets and pith. The numbers indicate genes that are exclusive to one thermogenic stage or shared among stages. The stages of thermogenesis in the spadices are denoted as ‘Pre’ (pre-thermogenic), ‘Hot’ (thermogenic), and ‘Post’ (post-thermogenic). TPM, transcripts per million.

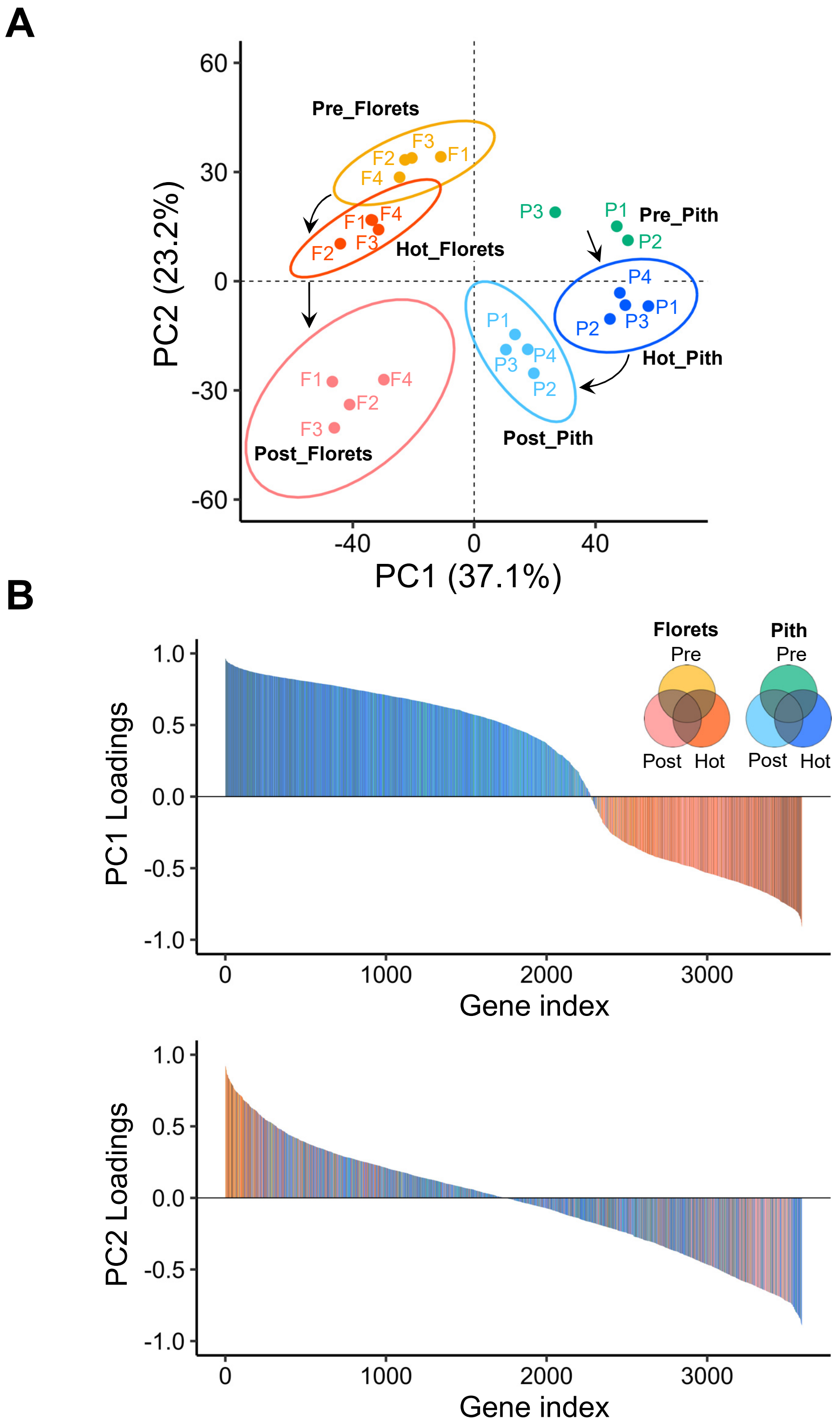

Supplemental Figure S3. Principal component analysis (PCA) based on expression patterns of differentially expressed genes (DEGs) in the florets and pith at various thermogenic stages of the spadices of *S. renifolius*.

(A) PCA plot of the DEGs. PC1 (37.1%) contributes to the separation of tissues based on thermogenic status, while PC2 (23.2%) reflects the transition across thermogenic stages. Sample labels are combinations of tissue type (florets or pith) and thermogenic stage (pre-thermogenic (Pre), thermogenic (Hot), and post-thermogenic (Post) spadices), e.g., 'Pre_Florets' indicates the floret samples from the pre-thermogenic stage of spadices. Arrows indicate the developmental progression of the spadices. The circled areas for each stage represent the 95% confidence intervals for the respective sample clusters. Arrows indicate the developmental trajectory of the spadices. Data for the Pre_Pith samples were excluded from this statistical analysis because the R software ('stat_ellipse' from 'ggplot2' package) could not process them due to an insufficient number of samples. (B) The PCA loading plots of individual DEGs, showing the significant DEGs that affect the PC1 and PC2 dimensions, as depicted in the PCA plots from Panel A. The genes are positioned according to their descending contribution to PCA loading and are indicated with a 'gene index' as displayed in the panel. The Venn diagram depicting gene categories based on different colors, corresponding to tissue types and thermogenic statuses of the spadices of *S. renifolius*. The color coding is consistent with the PCA loading panels for PC1 and PC2.

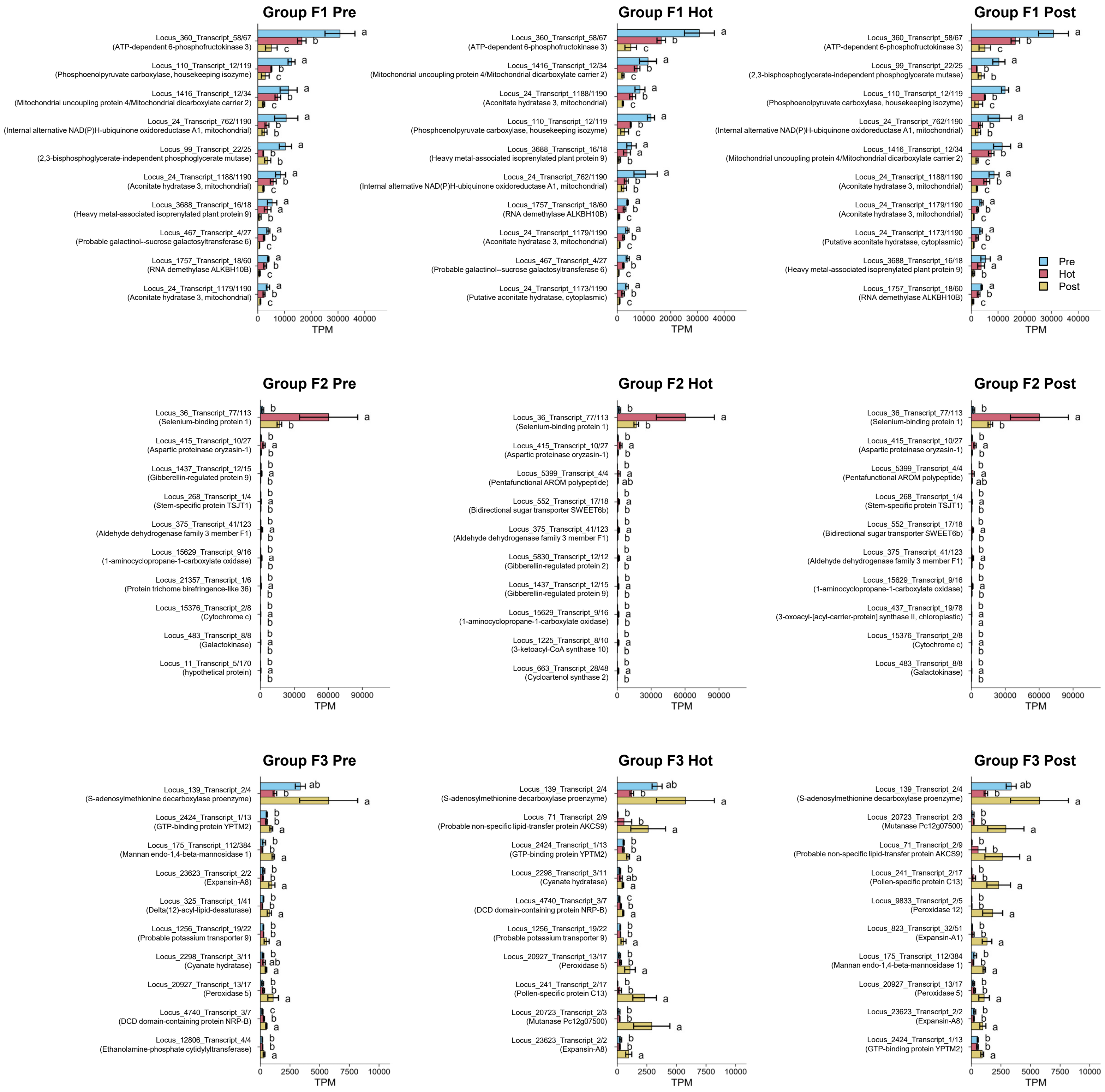

Supplemental Figure S4. Comprehensive gene expression profiling across various thermogenic stages in *S. renifolius.*
The 10 highest expressed genes in florets or pith during the pre-thermogenic (Pre), thermogenic (Hot), and post-thermogenic (Post) stages of spadices are presented. Expression levels are quantified as TPM values, with groups F1-F6 representing florets and groups P1a-P6 representing pith, as shown in Figs. 3B and 3C. The bar graphs represent the average TPM values of multiple independent samples (n = 4 except for pre-thermogenic pith: n = 3) ± standard deviation. Statistically significant differences among the stages, ascertained using the Tukey-Kramer test ($p < 0.05$), are denoted by unique letters next to the corresponding bar plots. Gene identifiers and putative functions are also given. TPM, transcripts per million.

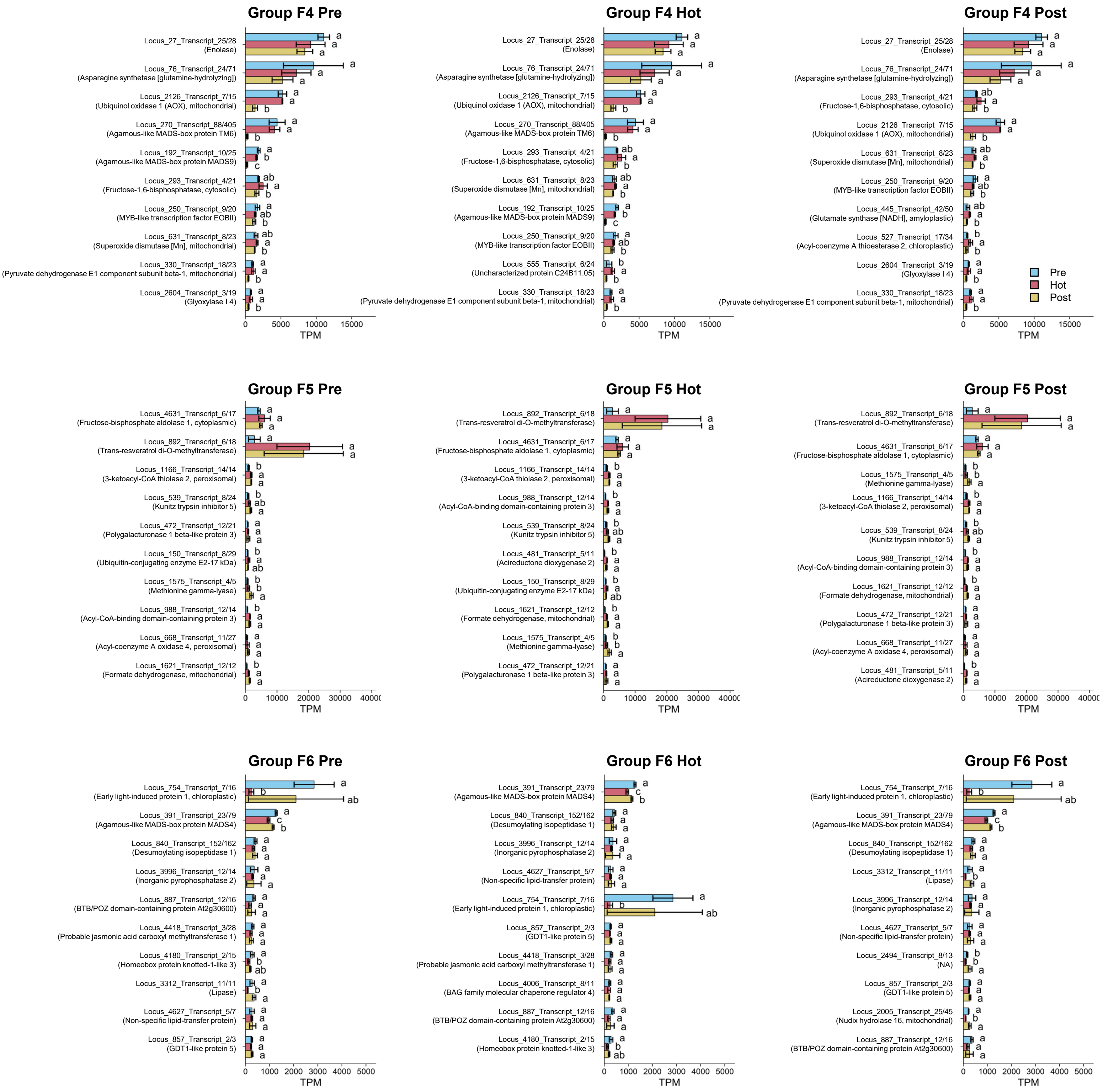

Supplemental Figure S4 (continued).

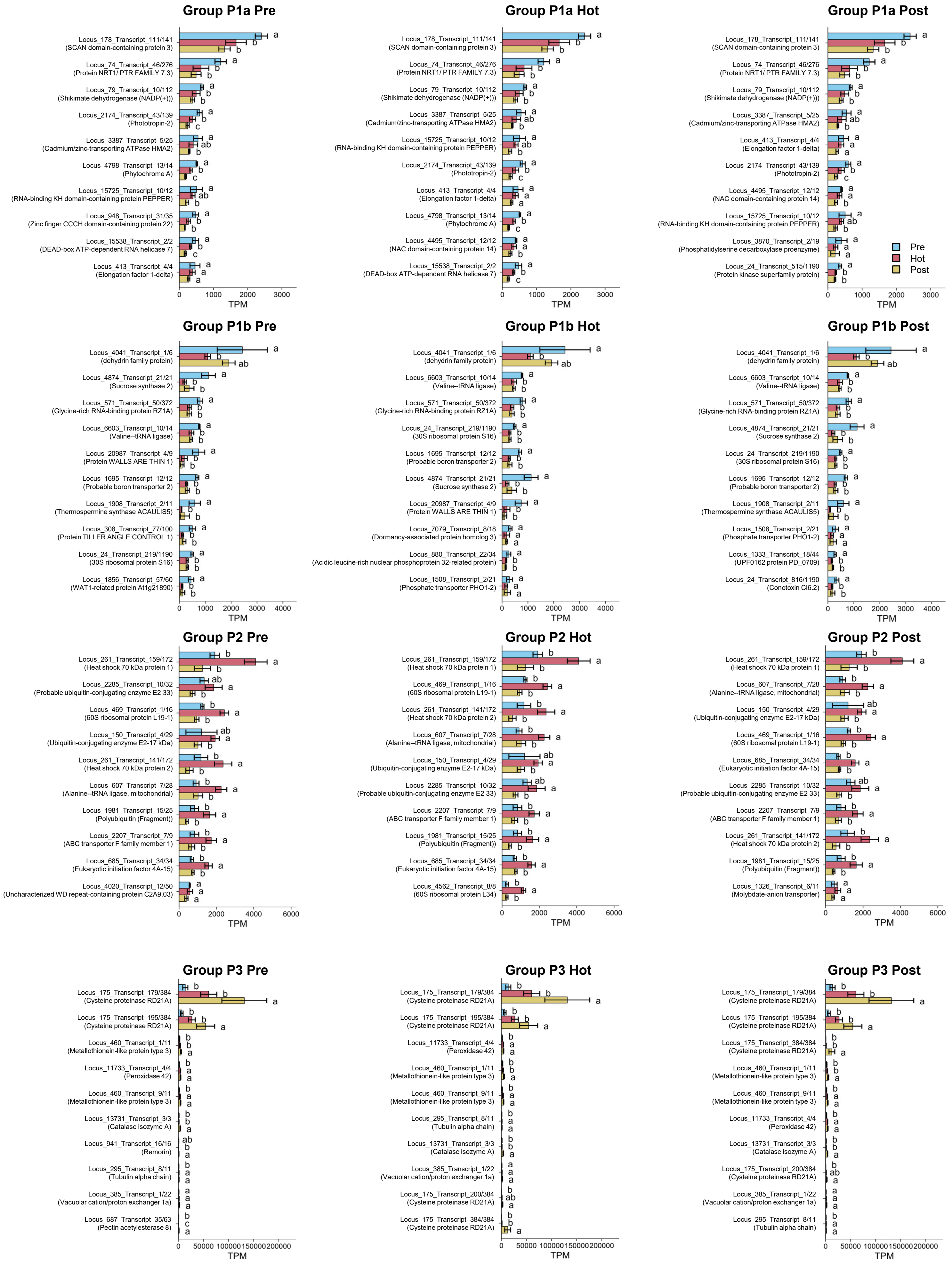

Supplemental Figure S4 (continued).

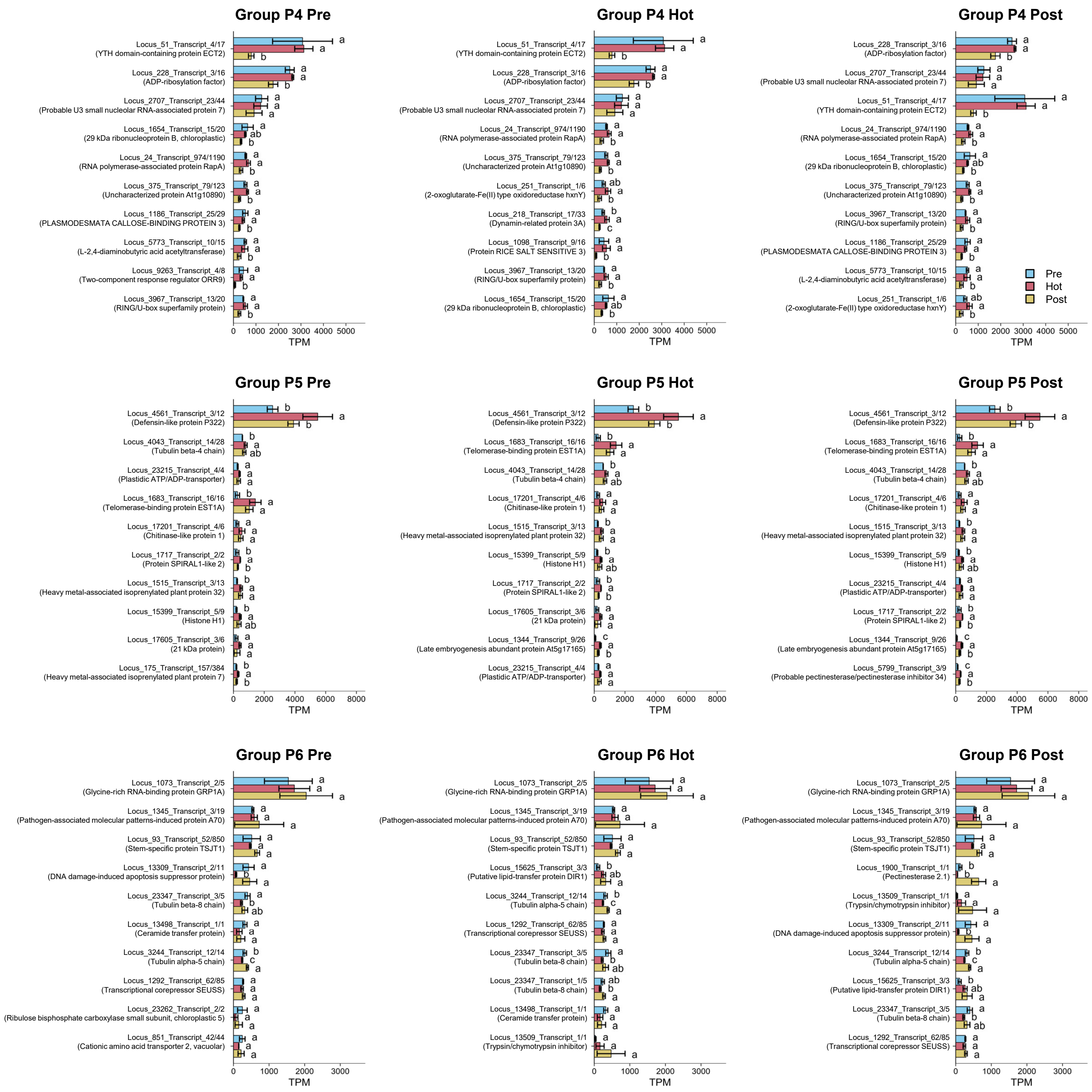

Supplemental Figure S4 (continued).

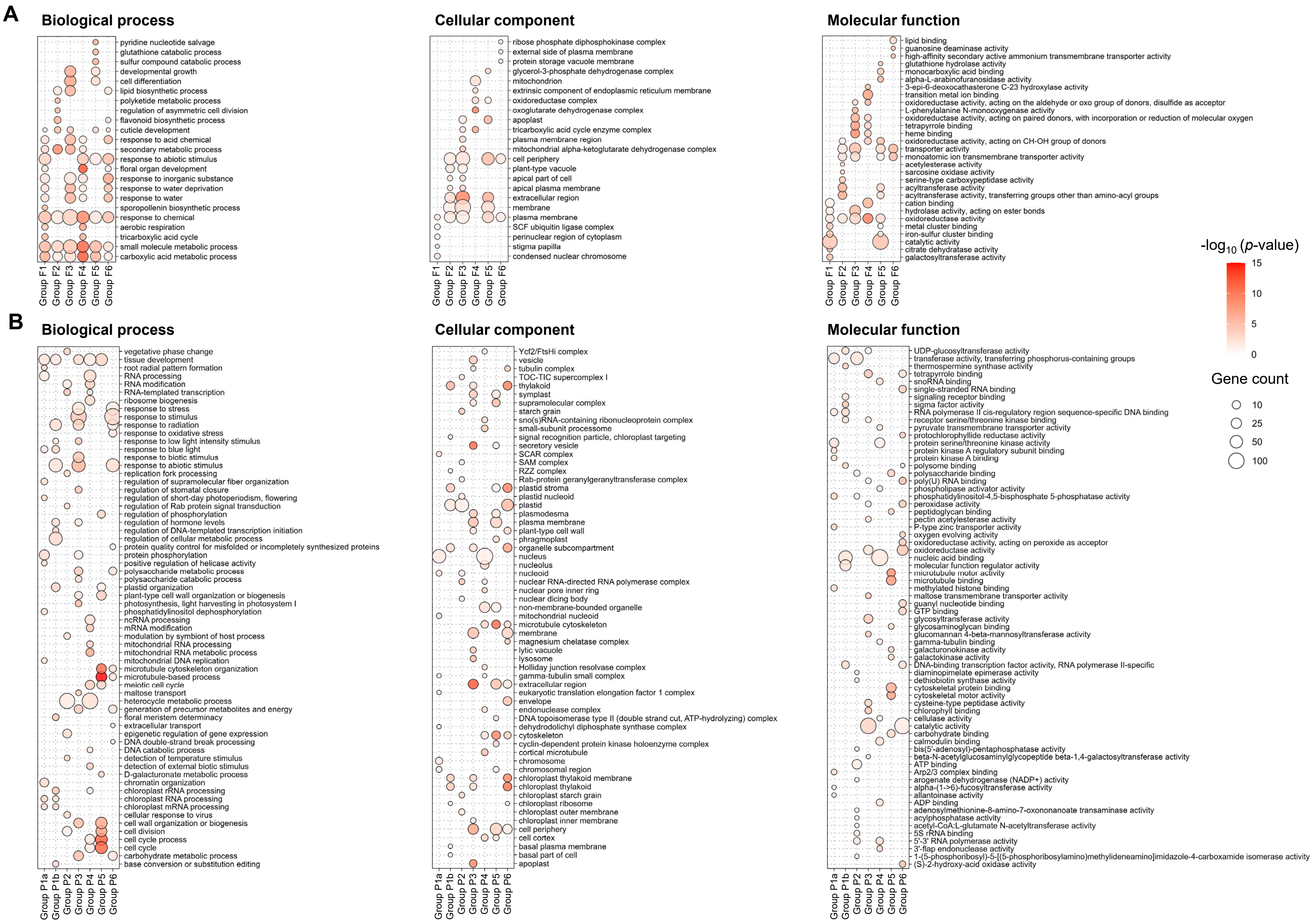

Supplemental Figure S5. **Enriched Gene Ontology (GO) terms identified by k-means clustering in the florets and pith of *S. renifolius.***

GO terms that are significantly enriched in the various groups categorized through k-means clustering of florets and pith tissues in Figs. 3B and 3C are presented for florets (A) and pith (B). Each panel corresponds to one of the three GO categories: Biological Process (left), Cellular Component (middle), and Molecular Function (right). The top five enriched GO terms for each group were identified, and overlapping terms were excluded from the combined list. Circle size corresponds to the number of genes associated with each term, and the intensity reflects the $-\log_{10}$ (p-value).

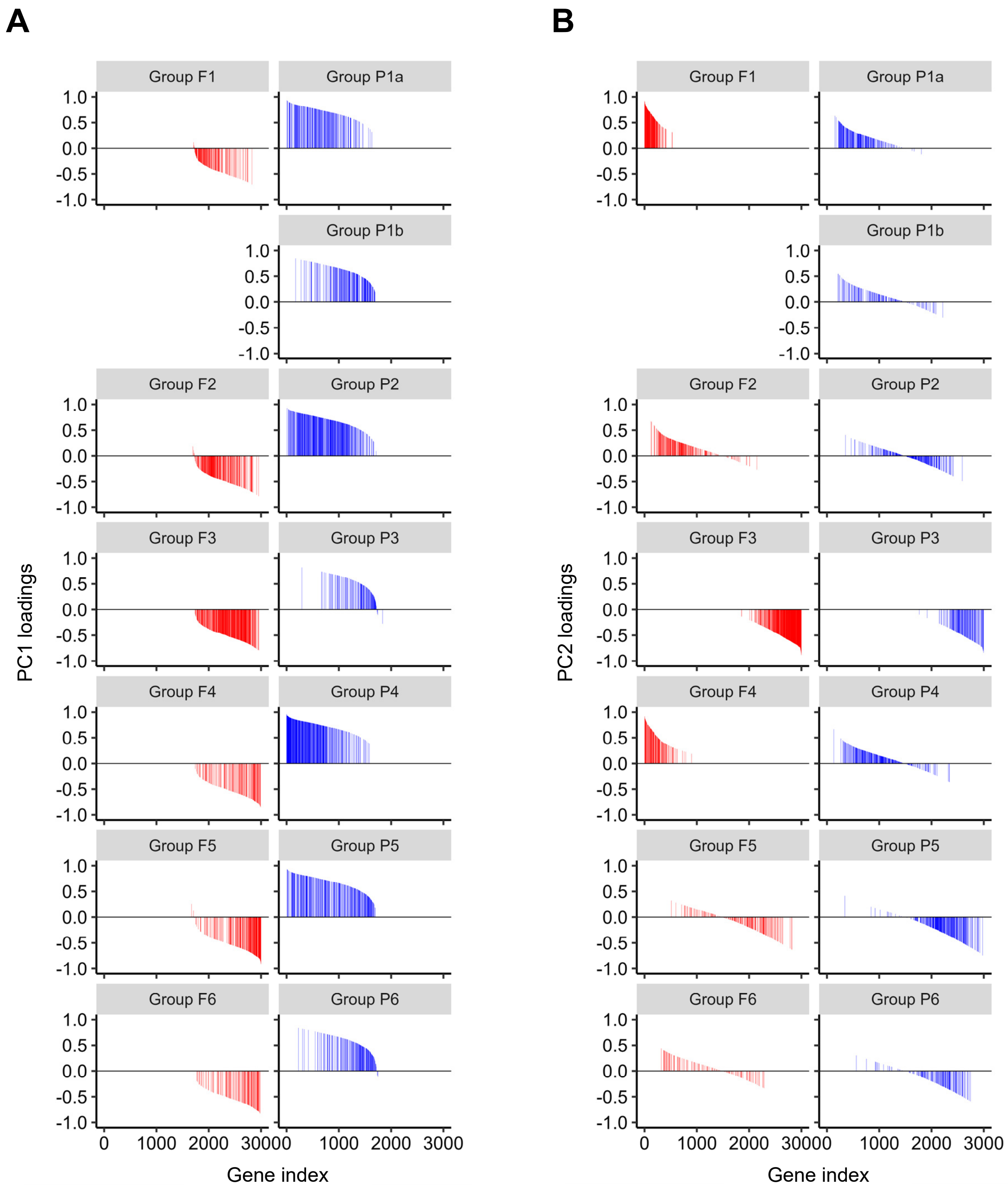

Supplemental Figure S6. Contribution of differentially expressed genes (DEGs) to tissue-specific and developmentally controlled thermogenesis in the spadix of *S. renifolius*.

DEGs are categorized based on their contribution to principal component analysis (PCA) loadings, with PC1 (panel A) separating tissue-specific expression, and PC2 (panel B) reflecting the progression through thermogenic stages in the spadices (see Supplemental Fig. S3B for additional details). Six groups of DEGs for the florets (Groups F1-F6, depicted in red) and seven groups for the pith (Groups P1a & P1b through P6, depicted in blue) are presented. The gene index on the x-axis corresponds to individual genes, with extensive information available in Supplemental Data S6. The y-axis represents the PCA loadings, with PC1 loadings in the panel A and PC2 loadings in the panel B, thereby indicating the impact of each gene on the variation captured by the corresponding principal component as shown in Supplemental Fig. S3A.

A

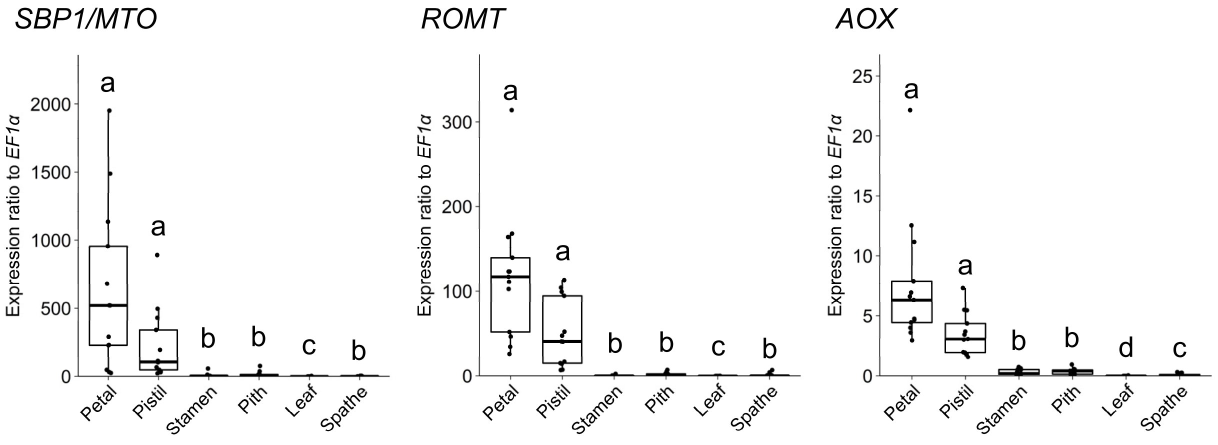

B

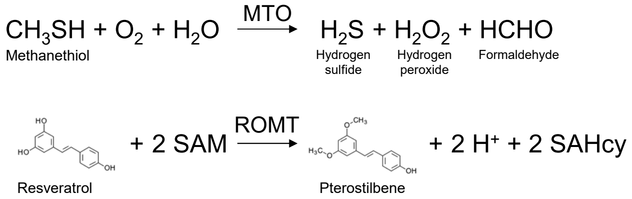

Supplemental Figure S7. Verification of gene expression in various tissues in the thermogenic stage of *S. renifolius*.
(A) Reverse transcription-quantitative PCR analysis of gene expression for *SBP1/MTO*, *ROMT*, and *AOX* in the petal, pistil, stamen, pith, leaf and spathe from individual plants. The sample sizes for each tissue type are as follows: petal, pistil, stamen, and pith (n = 14); leaf (n = 12); and spathe (n = 13). Statistically significant differences are indicated by different letters above the bars, as determined by a Steel-Dwass test ($p < 0.05$). Boxplots show the interquartile range (IQR), with the median shown as lines. The whiskers correspond the 1.5 times the IQR. Points indicate individual values. (B) Enzymatic reactions catalyzed by MTO and ROMT.

Abbreviations: AOX, alternative oxidase; ROMT, *trans*-resveratrol di-*O*-methyltransferase; SAHcy, *S*-adenosyl homocysteine; SAM, *S*-adenosyl methionine; SBP1/MTO, selenium-binding protein 1/methanethiol oxidase.

A

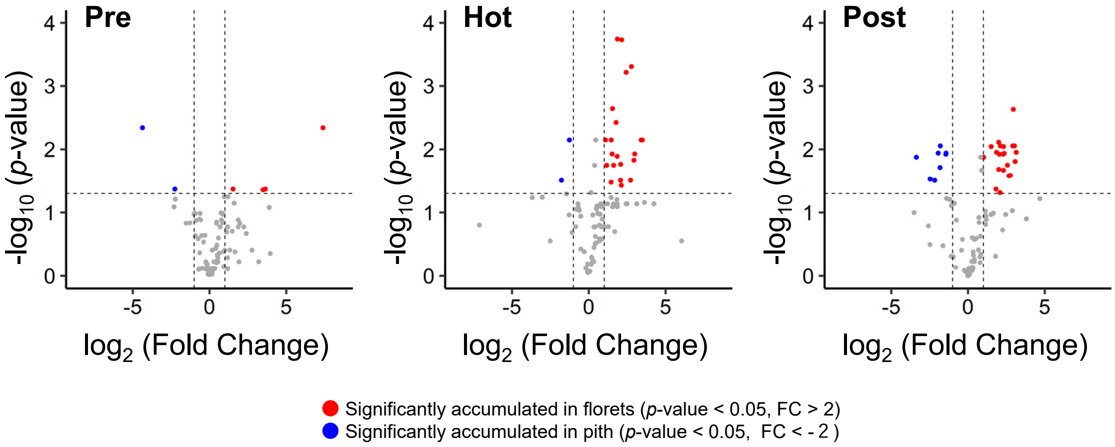

B

Pre Florets

Metabolite	FC	*p*-value
Ascorbate	168.63	0.00453
UMP	12.48	0.04279
AMP	11.26	0.04407
NAD	2.91	0.04279

Pre Pith

Metabolite	FC	*p*-value
Arginine	-20.59	0.00453
Tyramine	-4.75	0.04279

Hot Florets

Metabolite	FC	*p*-value
Ascorbate	11.18	0.00713
GDP	10.68	0.00713
Cytidine	7.98	0.01185
ADP-glucose	7.71	0.01484
IMP	6.85	0.00049
Cytosine	6.61	0.03111
AMP	5.4	0.00061
NAD	4.44	0.00019
4-Hydroxyproline	4.35	0.03727
GTP	4.23	0.01727
CDP	4.21	0.03111
GDP-glucose	3.66	0.00018
CMP	3.6	0.01295
Succinate	3.43	0.00376
Glucuronate	3.08	0.01793
UDP-glucose	2.92	0.00228
UMP	2.87	0.01185
Dihydroxyacetone phosphate	2.75	0.00713
ADP	2.74	0.03333
Cysteine	2.24	0.01793
2-Oxoglutaric acid	2.14	0.00713

Hot Pith

Metabolite	FC	*p*-value
N-acetyl spermidine	-3.43	0.03111
Citrate	-2.39	0.00713

Post Florets

Metabolite	FC	*p*-value
IMP	8.86	0.01119
Dihydroxyacetone phosphate	8.43	0.01562
AMP	8.19	0.00885
Adenosine	7.8	0.00235
NAD	7.55	0.00885
Glucose 6-phosphate	6.8	0.02597
Succinate	6.39	0.02637
Cytosine	5.95	0.01782
Uridine	5.16	0.01162
UMP	4.98	0.00905
GDP	4.96	0.02151
Uracil	4.9	0.01196
CMP	4.33	0.00885
Histidine	4.27	0.04862
Erythrose 4-phosphate	4.14	0.01196
S-Adenosylmethionine	4.03	0.00774
ADP-glucose	4.02	0.02087
UDP-glucose	3.66	0.01119
Cytidine	3.55	0.0428
ADP	2.85	0.00905
Serine	2.04	0.01338

Post Pith

Metabolite	FC	*p*-value
Acetyl-CoA	-10.28	0.01338
Citrate	-5.54	0.02967
Spermidine	-4.50	0.03101
Fumarate	-3.83	0.01147
UTP	-3.51	0.01947
Ribulose 1,5-bisphosphate	-3.49	0.00885
Aspartate	-2.70	0.01196
Malate	-2.69	0.01147

Supplemental Figure S8. Differential metabolite profiling across various thermogenic stages in *S. renifolius*.
(A) Volcano plots display differential metabolite accumulations between florets and pith during pre-thermogenic (Pre), thermogenic (Hot), and post-thermogenic (Post) stages. Red dots indicate metabolites with significant accumulation in the florets, while blue dots indicate those in the pith. The significance threshold for analysis (*p*-value < 0.05 and |fold change| > 2) is indicated by dashed lines. (B) A list of metabolites upregulated in florets or pith as depicted in panel (A). 'Pre Florets', 'Hot Florets', and 'Post Florets' refer to florets collected from spadices at pre-thermogenic, thermogenic, and post-thermogenic stages, respectively. Similarly, 'Pre Pith', 'Hot Pith', and 'Post Pith' denote pith tissues collected from spadices during the corresponding stages of thermogenesis. Metabolites with significant accumulation in florets are highlighted with a red background, while those in pith have a blue background. Metabolites are listed in descending order of their fold change magnitude between florets and pith.

A

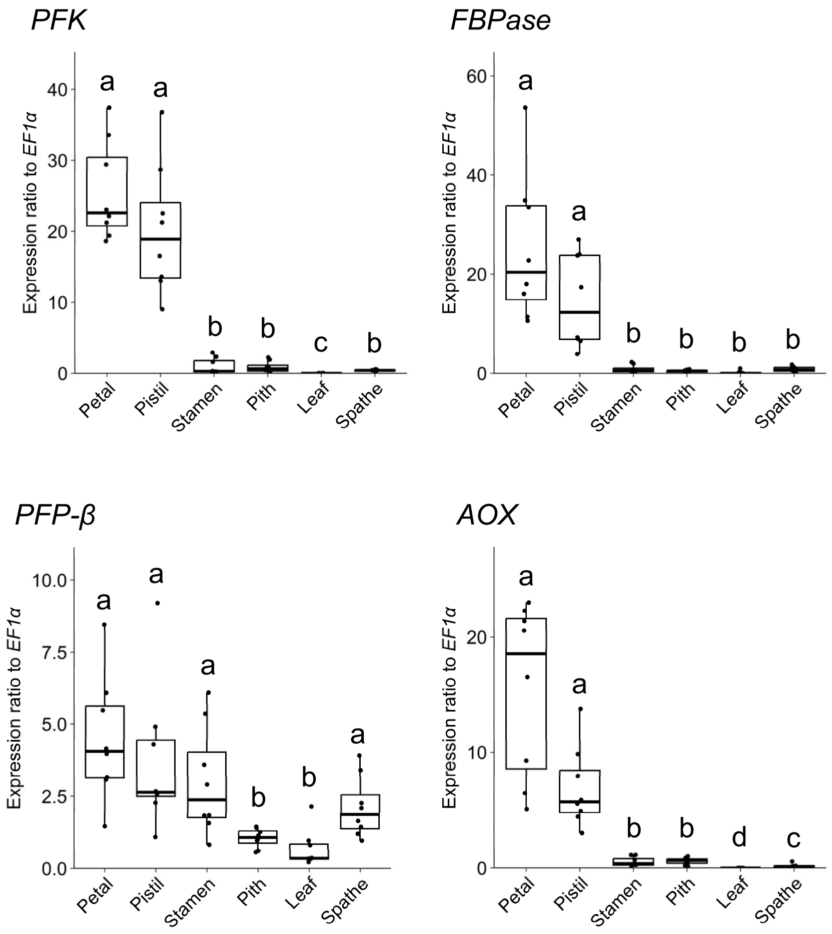

B

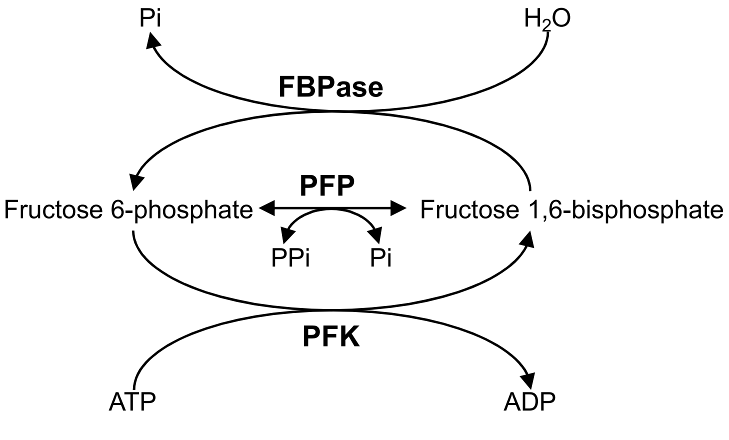

Supplemental Figure S9. Tissue-specific expression of genes involved in the ATP-consuming futile cycle at the thermogenic stage of *S. renifolius*.
(A) Verification of gene expression levels for *PFK*, *FBPase*, *PFP-β*, and *AOX* in the petal, pistil, stamen, pith, leaf, and spathe from individual plants by reverse transcription-quantitative PCR (n = 8). Statistically significant differences are indicated by different letters above the bars, as determined by a Steel-Dwass test ($p < 0.05$). Boxplots show the interquartile range (IQR), with the median shown as lines. The whiskers correspond the 1.5 times the IQR. Points indicate individual values. (B) Enzymatic reactions constituting the futile cycle.

Abbreviations: AOX, alternative oxidase; FBPase, fructose-1,6-bisphosphatase; PFK, ATP-dependent phosphofructokinase; PFP, fructose-6-phosphate 1-phosphotransferase.

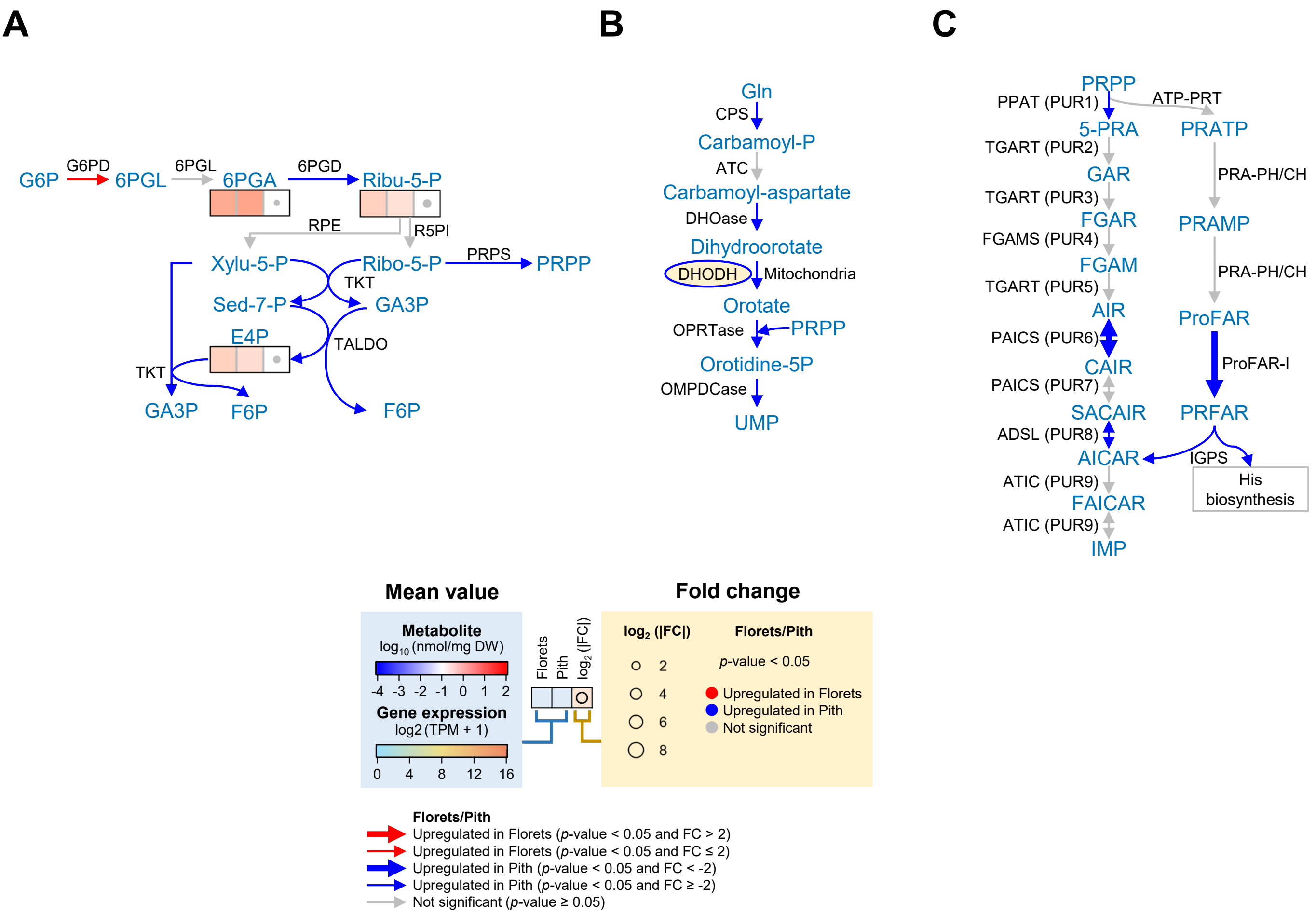

Supplemental Figure S10. Metabolic pathways involved in the *de novo* synthesis of purine and pyrimidine nucleotides in the spadices of *S. renifolius*.
Gene expression and metabolite accumulation levels for the (A) pentose phosphate, (B) pyrimidine nucleotide synthesis, and (C) purine nucleotide synthesis pathways. Color gradients in the lower panel show variations in metabolite accumulation ($\log_{10}$ (nmol/mg DW)) and gene expression levels ($\log_2$ (TPM) + 1). Circles of different sizes represent the $\log_2$ fold change (FC) values, ranging from 2 to 8. Filled red circles indicate upregulation in the florets, blue filled circles represent upregulation in the pith, and grey filled circles denote non-significant differences. Arrows denote upregulation trends in either the florets (red) or the pith (blue), with bold arrows indicating a *p*-value < 0.05 and FC > 2 or FC < -2. Narrow arrows show a *p*-value < 0.05 and FC ≤ 2 or FC ≥ -2, and gray arrows point to non-significant results (*p*-value ≥ 0.05). DW, dry weight; TPM, transcripts per million.

Supplemental Figure S10. Abbreviations:

Abbreviations of enzymes in Panel A: 6PGD, 6-phosphogluconate dehydrogenase; 6PGL, 6-phosphogluconolactonase; G6PD, glucose-6-phosphate 1-dehydrogenase; PRPS, ribose-phosphate pyrophosphokinase; R5PI, ribose-5-phosphate isomerase; RPE, ribulose-phosphate 3-epimerase; TALDO, transaldolase; TKT, transketolase.

Abbreviations of metabolites in Panel A: 6PGA, 6-phospho-D-gluconate; 6PGL, 6-phosphogluconolactone; E4P, erythrose 4-phosphate; F6P, fructose 6-phosphate; G6P, glucose-6-phosphate; GA3P, glyceraldehyde 3-phosphate; Ribo-5-P, ribose 5-phosphate; Ribu-5-P, ribulose 5-phosphate; Sed-7-P, sedoheptulose 7-phosphate; Xylu-5-P, xylulose 5-phosphate.

Abbreviations of enzymes in Panel B: ATC, aspartate carbamoyltransferase; CPS, carbamoyl-phosphate synthase; DHOase, dihydroorotase; DHODH, dihydroorotate dehydrogenase (quinone); UMPS, uridine 5'-monophosphate synthase. Abbreviations of metabolites in Panel B: Carbamoyl-P, carbamoyl phosphate; Gln, L-Glutamine; Orotidine-5P, orotidine 5-phosphate; PRPP, 5-phosphoribosyl 1-pyrophosphate; UMP, uridine 5'-monophosphate.

Abbreviations of metabolites in Panel B: Carbamoyl-P, carbamoyl phosphate; Gln, L-Glutamine; Orotidine-5P, orotidine 5-phosphate; PRPP, 5-phosphoribosyl 1-pyrophosphate; UMP, uridine 5'-monophosphate.

Abbreviations of enzymes in Panel C: ADSL (PUR8), adenylosuccinate lyase; ATIC (PUR9), bifunctional purine biosynthesis protein; ATP-PRT, ATP phosphoribosyltransferase; FGAMS (PUR4), phosphoribosylformylglycinamidine synthase; IGPS, imidazole glycerol phosphate synthase; PAICS (PUR6), phosphoribosylaminoimidazole carboxylase; PAICS (PUR7), phosphoribosylaminoimidazole-succinocarboxamide synthase; PPAT (PUR1), amidophosphoribosyltransferase; PRA-PH/CH, histidine biosynthesis bifunctional protein; ProFAR-I, 1-(5-phosphoribosyl)-5-[(5-phosphoribosylamino)methylideneamino]imidazole-4-carboxamide isomerase; TGART (PUR2), phosphoribosylamine-glycine ligase; TGART (PUR3), phosphoribosylglycinamide formyltransferase; TGART (PUR5), phosphoribosylformylglycinamidine cyclo-ligase.

Abbreviations of metabolites in Panel C: 5-PRA, 5-phosphoribosylamine; AICAR, 5-aminoimidazole-4-carboxamide ribotide; AIR, aminoimidazole ribotide; CAIR, 1-(5-phospho-D-ribosyl)-5-amino-4-imidazolecarboxylate; FAICAR, 5-formamido-1-(5-phosphoribosyl)imidazole-4-carboxamide; FGAM, 2-(formamido)-*N*1-(5'-phosphoribosyl)acetamidine; FGAR, 5'-phosphoribosyl-*N*-formylglycinamide; GAR, 5'-phosphoribosylglycinamide; IMP, inosine monophosphate (inosinic acid); PRAMP, *N*'-5'-phosphoribosyl-AMP; PRATP, *N*'-5'-phosphoribosyl-ATP; PRFAR, *N*'-[(5-phosphoribulosyl)formimino]-5-aminoimidazole-4-carboxamide; ProFAR, *N*'-[(5'-phosphoribosyl)formimino]-5-aminoimidazole-4-carboxamide; PRPP, 5-phosphoribosyl 1-pyrophosphate; SACAIR, 1-(5'-phosphoribosyl)-5-amino-4-(*N*-succinocarboxamide)-imidazole.

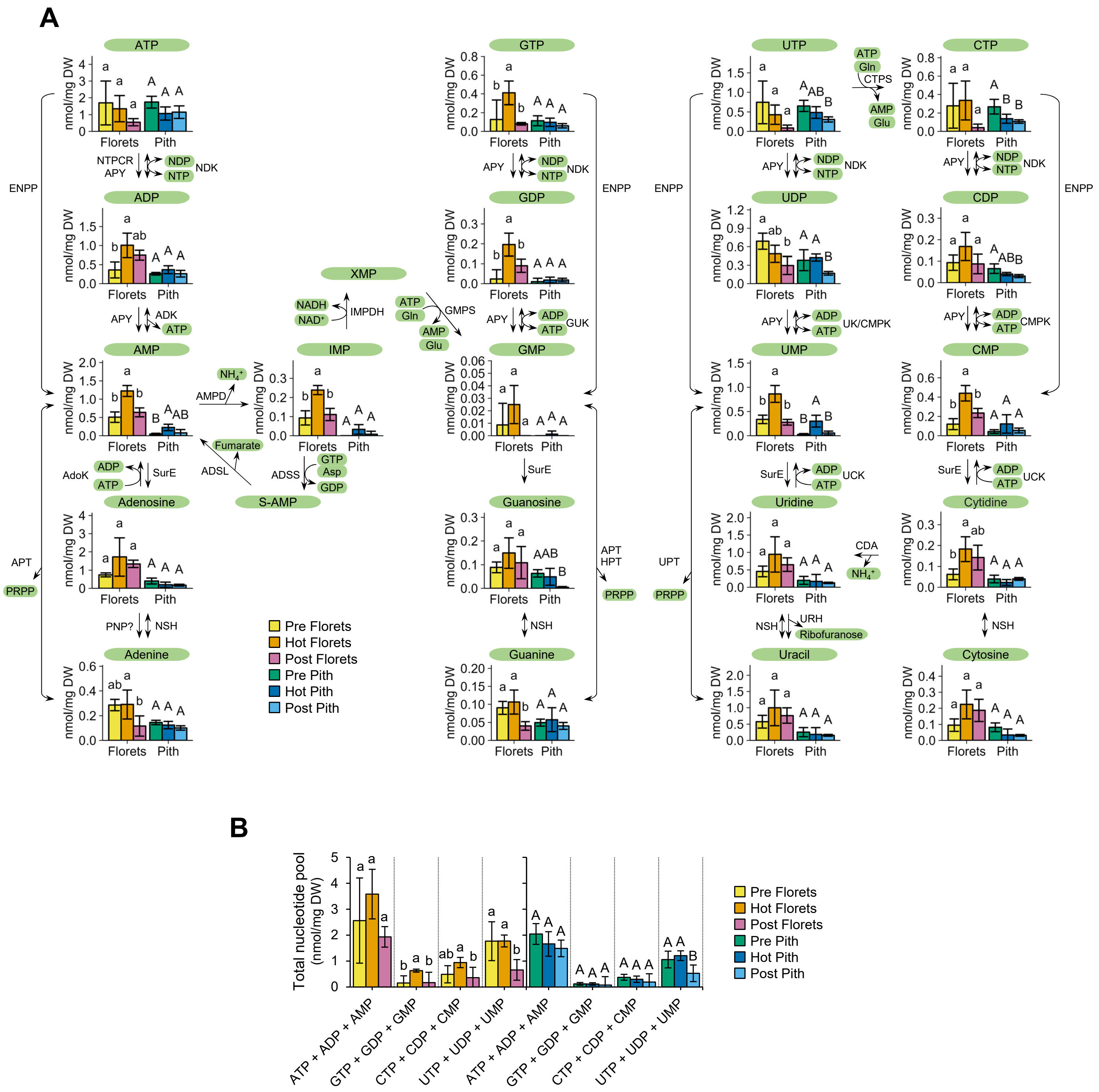

Supplemental Figure S11. **Nucleotide interconversions and accumulation levels of metabolites.**
(A) Overview of the purine and pyrimidine interconversion pathways, alongside the corresponding concentrations as determined by CE-MS (capillary electrophoresis-mass spectrometry). (B) Comparison of nucleotide pools. In both panels (A) and (B), the data analyzed were divided into six differently colored groups to represent different stages and parts of the spadix, i.e., florets from pre-thermogenic spadices (Pre Florets), florets from thermogenic spadices (Hot Florets), florets from post-thermogenic spadices (Post Florets), pith from pre-thermogenic spadices (Pre Pith), pith from thermogenic spadices (Hot Pith), and pith from post-thermogenic spadices (Post Pith). The mean values are represented by bar charts, and the standard deviations are indicated by error bars. The sample size was n = 4 for all groups except for Pre_Pith, where n = 3. Statistically significant differences among the groups are indicated by different letters above the bars (Tukey-Kramer test; *p*-*value* < 0.05). Specifically, lowercase letters denote comparisons within the 'florets' group, while uppercase letters are used for comparisons within the 'pith' group. DW, dry weight.

Abbreviations: ADSL, adenylosuccinate lyase; ADSS, adenylosuccinate synthetase; AMPD, AMP deaminase; APY, apyrase; APT, adenine phosphoribosyltransferase; adoK, adenosine kinase; CDA, cytidine deaminase; CMPK, UMP-CMP kinase; CTPS, CTP synthase; ENPP, venom phosphodiesterase; GMPS, GMP synthase; GUK, guanylate kinase; HPT, hypoxanthine-guanine phosphoribosyltransferase; IMPDH, inosine-5'-monophosphate dehydrogenase; NDK, nucleoside diphosphate kinase; NSH, ribonucleoside hydrolase; NTPCR, nucleoside-triphosphatase; PNP, purine nucleoside phosphorylase; SurE, 5'-nucleotidase SurE; UCK, uridine kinase; UK, uridylate kinase; UPT, uracil phosphoribosyltransferase; URH, uridine nucleosidase.

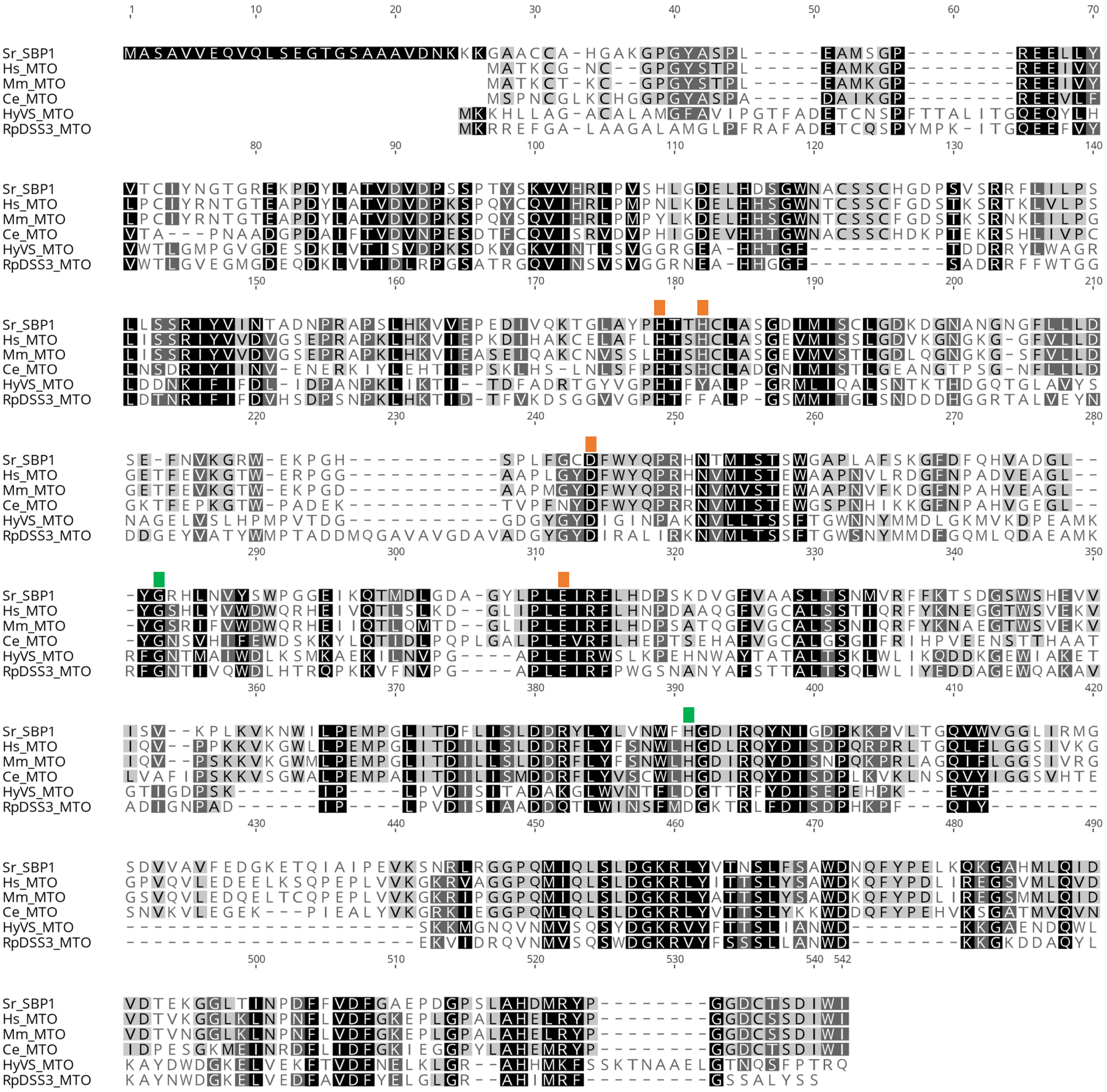

Supplemental Figure S12. Comparative alignment of deduced methanethiol oxidase (MTO) and selenium-binding protein 1 (SBP1) amino acid sequences.

The alignment shows putative Cu^{2+} binding sites with orange boxes (Philipp et al., 2023), while green boxes indicate the histidine and glycine residues; substitutions in these residues have been linked to loss of MTO activity in *Homo sapiens* and *Caenorhabditis elegans* (Pol et al., 2018; Philipp et al., 2022). Abbreviations and accession numbers are as follows: RpDSS3 (*Ruegeria pomeroyi* DSS-3; WP_011242048.1), HyVS (*Hyphomicrobium* sp. VS.; A0A291P0C1.1), Hs (*Homo sapiens*; NP_003935.2), Mm (*Mus musculus*; NP_033176.2), Ce (*Caenorhabditis elegans*; NP_001255777.1), and Sr (*Symplocarpus renifolius*; BDN86070.1).

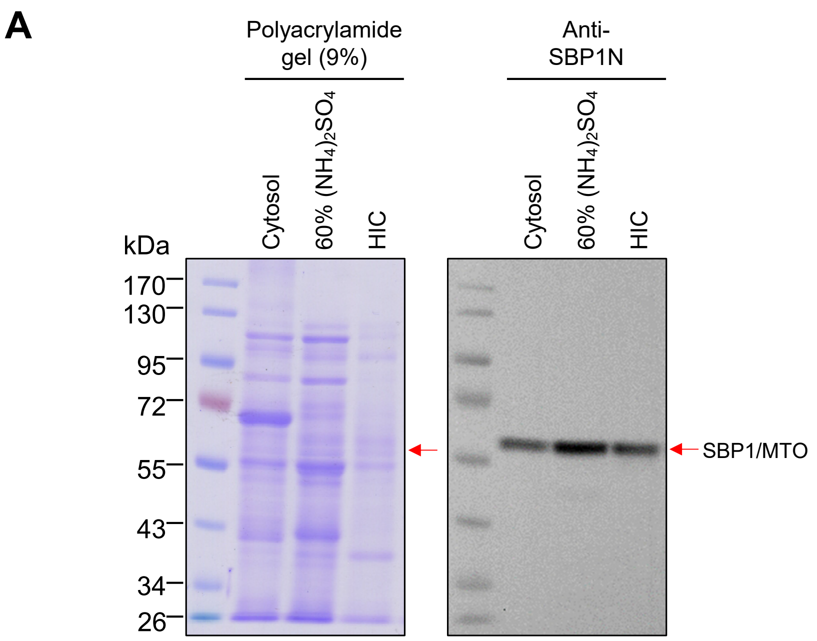

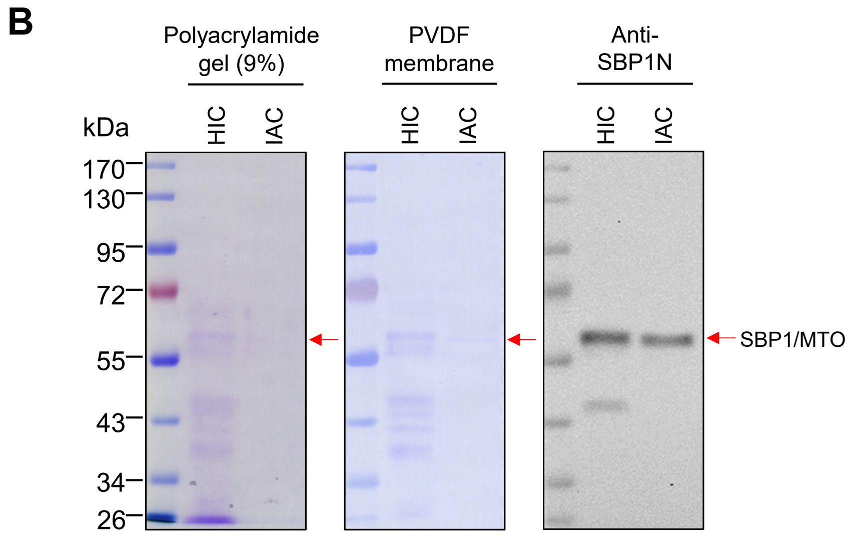

Supplemental Figure S13. Purification of selenium-binding protein 1/methanethiol oxidase (SBP1/MTO) protein.
(A) Western blot analysis of fractions derived from ammonium sulfate fractionation followed by hydrophobic interaction chromatography (HIC) using HiTrap Phenyl HP. (B) Immunoaffinity chromatography (IAC) purification of SBP1/MTO protein employing an anti-SBP1N antibody-immobilized column. A Coomassie Brilliant Blue (CBB)-stained image of proteins transferred to a polyvinylidene difluoride (PVDF) membrane is also displayed. Bands corresponding to the positions of SBP1/MTO protein are indicated with red arrows.

A

```
  1  MASAVVEQVQ LSEGTGSAAA VDNKKKGAAC CAHGAKGPGY ASPLEAMSGP
 51  REELLYVTCI YNGTGREKPD YLATVDVDPS SPTYSKVVHR LPVSHLGDEL
101  HDSGWNACSS CHGDPSVSRR FLILPSLLSS RIYVINTADN PRAPSLHKVV
151  EPEDIVQKTG LAYPHTHTHCL ASGDIMISCL GDKDGNANGN GFLLLDSEFN
201  VKGRWEKPGH SPLFGCDFWY QPRHNTMIST SWGAPLAFSK GFDFQHVADG
251  LYGRHLNVYS WPGGEIKQTM DLGDAGYLPL EIRFLHDPSK DVGFVAASLT
301  SNMVRFFKTS DGSWSHEVVI SVKPLKVKNW ILPEMPGLIT DFLISLDDRY
351  LYLVNWFHGD IRQYNIGDPK KPVLTGQVWV GGLIRMGSDV VAVFEDGKET
401  QIAIPEVKSN RLRGGPQMIQ LSLDGKRLYV TNSLFSAWDN QFYPELKQKG
451  AHMLQIDVDT EKGGLTINPD FFVDFGAEPD GPSLAHDMRY PGGDCTSDIW
501  I
```

B

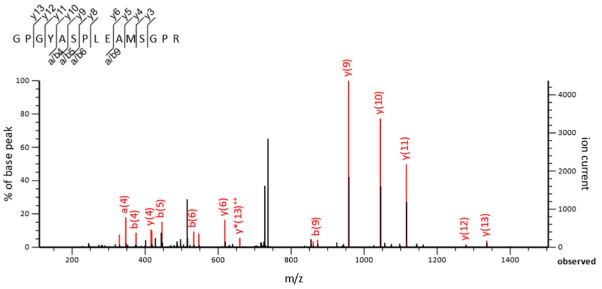

#	a	a++	b	b++	Seq.	y	y++	y*	y*++	#
1	30.03	15.52	58.03	29.52	G					15
2	127.1	64.05	155.1	78.04	P	1433	716.8	1416	708.3	14
3	184.1	92.56	212.1	106.6	G	1336	668.3	1319	659.8	13
4	347.2	174.1	375	188.1	Y	1279	639.8	1262	631.3	12
5	418.2	209.6	446	223.6	A	1116	558.3	1099	549.8	11
6	505.2	253.1	533	267.1	S	1045	522.8	1027	514.2	10
7	602.3	301.7	630.3	315.6	P	957	479.2	940.5	470.7	9
8	715.4	358.2	743.4	372.2	L	860	430.7	843.4	422.2	8
9	844.4	422.7	872	436.7	E	747.3	374.2	730.3	365.7	7
10	915.5	458.2	943.5	472.2	A	618	309.7	601.3	301.1	6
11	1046	523.8	1074	537.7	M	547	274.1	530.2	265.6	5
12	1134	567.3	1162	581.3	S	416	208.6	399.2	200.1	4
13	1191	595.8	1219	609.8	G	329	165.1	312.2	156.6	3
14	1288	644.3	1316	658.3	P	272.2	136.6	255.1	128.1	2
15					R	175.1	88.06	158.1	79.55	1

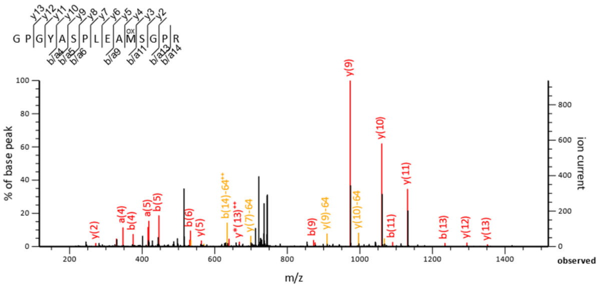

#	a	a++	b	b++	Seq.	y	y++	y*	y*++	#
1	30.03	15.52	58.03	29.52	G					15
2	127.1	64.05	155.1	78.04	P	1449	724.8	1432	716.3	14
3	184.1	92.56	212.1	106.6	G	1352	676.3	1335	667.8	13
4	347.2	174.1	375	188.1	Y	1295	647.8	1278	639.3	12
5	418.2	209.6	446	223.6	A	1132	566.3	1115	557.8	11
6	505.2	253.1	533	267.1	S	1061	530.8	1043	522.2	10
7	602.3	301.7	630.3	315.6	P	973	487.2	956.5	478.7	9
8	715.4	358.2	743.4	372.2	L	876	438.7	859.4	430.2	8
9	844.4	422.7	872	436.7	E	763.3	382.2	746.3	373.7	7
10	915.5	458.2	943.5	472.2	A	634	317.7	617.3	309.1	6
11	1062	531.7	1090	545.7	M	563	282.1	546.2	273.6	5
12	1150	575.3	1178	589.3	S	416	208.6	399.2	200.1	4
13	1207	603.8	1235	617.8	G	329	165.1	312.2	156.6	3
14	1304	652.3	1332	666.3	P	272	136.6	255.1	128.1	2
15					R	175.1	88.06	158.1	79.55	1

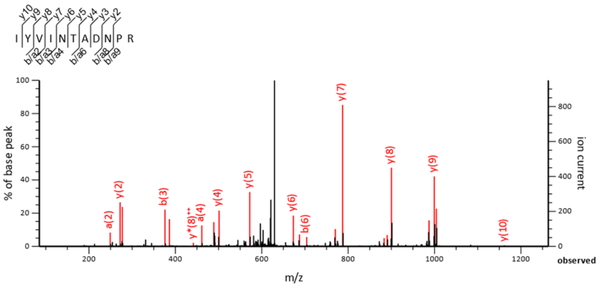

#	a	a++	a*	a*++	b	b++	b*	b*++	Seq.	y	y++	y*	y*++	#
1	86.1	43.55			114.1	57.55			I					11
2	249	125.1			277	139.1			Y	1163	581.8	1146	573.3	10
3	348.2	174.6			376	188.6			V	1000	500.3	982.5	491.8	9
4	461	231.2			489	245.2			I	900	450.7	883.4	442.2	8
5	575.4	288.2	558.3	279.7	603.4	302.2	586.3	293.7	N	787	394.2	770.3	385.7	7
6	676.4	338.7	659.4	330.2	704	352.7	687.4	344.2	T	673	337.2	656.3	328.7	6
7	747.4	374.2	730.4	365.7	775.4	388.2	758.4	379.7	A	572	286.6	555.3	278.1	5
8	862.5	431.7	845.4	423.2	890	445.7	873.4	437.2	D	501	251.1	484.2	242.6	4
9	976.5	488.8	959.5	480.2	1005	502.8	987.5	494.2	N	386	193.6	369.2	185.1	3
10	1074	537.3	1057	528.8	1102	551.3	1085	542.8	P	272	136.6	255.1	128.1	2
11									R	175.1	88.06	158.1	79.55	1

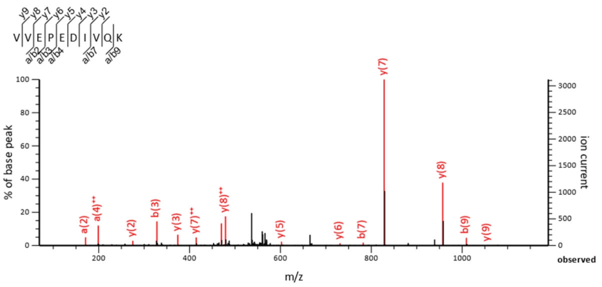

#	a	a++	a*	a*++	b	b++	b*	b*++	Seq.	y	y++	y*	y*++	#
1	72.08	36.54			100.1	50.54			V					10
2	171.1	86.08			199	100.1			V	1057	528.8	1040	520.3	9
3	300.2	150.6			328	164.6			E	957	479	940.5	470.7	8
4	397.2	199.1			425.2	213.1			P	828	415	811.4	406.2	7
5	526.3	263.6			554.3	277.6			E	731	366.2	714.4	357.7	6
6	641.3	321.2			669.3	335.2			D	602	301.7	585.3	293.2	5
7	754.4	377.7			782	391.7			I	487.3	244.2	470.3	235.7	4
8	853.5	427.2			881.5	441.2			V	374	187.6	357.2	179.1	3
9	981.5	491.3	964.5	482.8	1010	505.3	992.5	496.8	Q	275	138.1	258.1	129.6	2
10									K	147.1	74.06	130.1	65.55	1

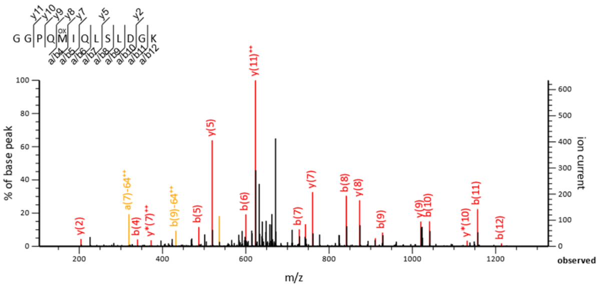

#	a	a++	a*	a*++	b	b++	b*	b*++	Seq.	y	y++	y*	y*++	#
1	30.03	15.52			58.03	29.52			G					13
2	87.06	44.03			115.1	58.03			G	1303	651.8	1286	643.3	12
3	184.1	92.56			212.1	106.6			P	1246	623.3	1229	614.8	11
4	312.2	156.6	295.1	148.1	340	170.6	323.1	162.1	Q	1149	574.8	1132	566.3	10
5	459.2	230.1	442.2	221.6	487	244.1	470.2	235.6	M	1021	510.8	1004	502.3	9
6	572.3	286.6	555.3	278.1	600	300.6	583.3	292.1	I	874	437.3	856.5	428.7	8
7	700.3	350.7	683.3	342.2	728	364.7	711.3	356.2	Q	760	380.7	743.4	372.2	7
8	813.4	407.2	796.4	398.7	841	421.2	824.4	412.7	L	632.4	316.7	615.3	308.2	6
9	900.5	450.7	883.4	442.2	928	464.7	911.4	456.2	S	519	260.1	502.3	251.6	5
10	1014	507.3	996.5	498.8	1042	521.3	1025	512.8	L	432	216.6	415.2	208.1	4
11	1129	564.8	1112	556.3	1157	578.8	1140	570.3	D	319	160.1	302.1	151.6	3
12	1186	593.3	1169	584.8	1214	607.3	1197	598.8	G	204	102.6	187.1	94.06	2
13									K	147.1	74.06	130.1	65.55	1

Supplemental Figure S14. Identification of *S. renifolius* selenium-binding protein 1/methanethiol oxidase (SBP1/MTO) by nanoscale liquid chromatography coupled to tandem mass spectrometry (nano LC-MS/MS).

(A) Peptides derived from Trypsin/Lys-C digest of SBP1/MTO. Peptides significantly matched to SBP1/MTO are shown in bold red letters. Sequence coverage of the SBP1/MTO protein was 9%. (B) MS/MS spectrum of SBP1/MTO peptide fragments (left) and the matched fragment ions (right). Bold italic red means the series contributed to the score, bold red means that the number of matches in the ion series is greater than would be expected by chance and non-bold red means that the number of matches in the ion series is no greater than would be expected by chance.

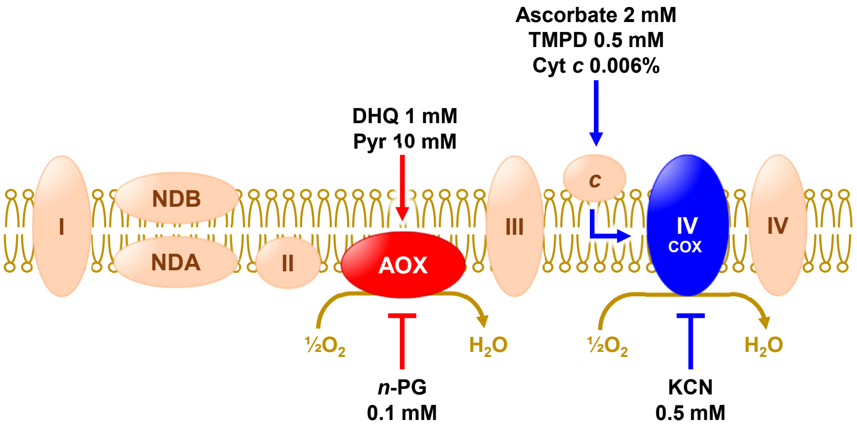

Supplemental Figure S15. Experimental procedures for measuring respiration capacities using purified mitochondria from thermogenic florets of *S. renifolius.*

AOX activity was measured using 1 mM DHQ as a substrate in the presence of 10 mM pyruvate and KCN. COX activity was measured under conditions of 2 mM ascorbic acid and 0.5 mM TMPD, with 0.006% (w/v) cytochrome *c* derived from horse heart muscle. *n*-PG was used to inhibit AOX activities.

Abbreviations: DHQ, durohydroquinone; KCN, potassium cyanide; *n*-PG, *n*-propyl gallate; Pyr, pyruvate; TMPD, *N*,*N*,*N'*,*N'*-tetramethyl-*p*-phenylenediamine.

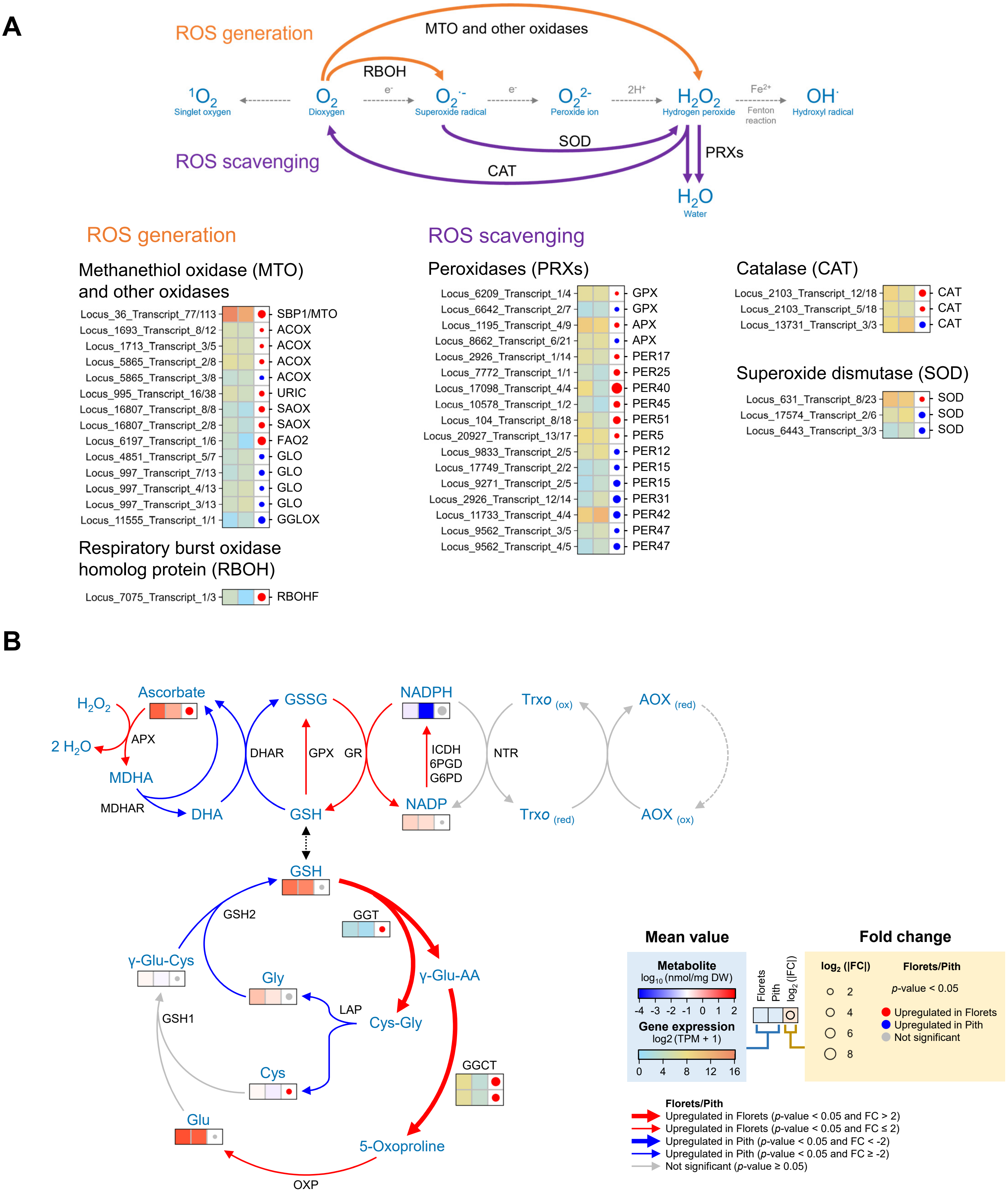

Supplemental Figure S16. Reactive oxygen species (ROS) dynamics, glutathione metabolism, and gene expression levels in the thermogenic florets of *S. renifolius*.

(A) Schematic of the ROS production and scavenging system. Enzymes involved in ROS production are indicated by orange arrows, while those associated with ROS quenching are shown with purple arrows. A list of genes associated with this system and their expression levels are given. Displayed genes are those upregulated in either the florets or the pith. (B) Map illustrating the glutathione metabolic pathway. The glutathione-mediated redox reaction and the γ-glutamyl cycle are depicted. Relative gene expression levels and metabolite accumulations within this pathway are also shown. Color gradients in the lower panel represent variations in metabolite accumulation ($\log_{10}$ (nmol/mg DW)) and gene expression levels ($\log_2$ (TPM) + 1). Circles of different sizes represent the $\log_2$ fold change (FC) values, ranging from 2 to 8. Filled red circles indicate upregulation in the florets, blue filled circles represent upregulation in the pith, and grey filled circles denote non-significant differences. Arrows denote upregulation trends in either the florets (red) or the pith (blue), with bold arrows indicating a *p*-value < 0.05 and FC > 2 or FC < -2. Narrow arrows show a *p*-value < 0.05 and FC ≤ 2 or FC ≥ -2, and gray arrows point to non-significant results (*p*-value ≥ 0.05). DW, dry weight; TPM, transcripts per million.

Supplemental Figure S16. Abbreviations:

Abbreviations of enzymes in Panel A: ACOX, acyl-coenzyme A oxidase; APX, L-ascorbate peroxidase; CAT, catalase; FAO2, long-chain-alcohol oxidase FAO2; GLO, (*S*)-2-hydroxy-acid oxidase; GGLOX, L-gluconolactone oxidase; GPX, glutathione peroxidase; PER, other peroxidases; RBOHF, respiratory burst oxidase homolog protein F; SBP1/MTO, selenium-binding protein 1/methanethiol oxidase; SAOX, sarcosine oxidase; SOD, superoxide dismutase; URIC, uricase.

Abbreviations of enzymes in Panel B: 6PGD, 6-phosphogluconate dehydrogenase; AOX, alternative oxidase; APX, L-ascorbate peroxidase; DHAR, glutathione *S*-transferase DHAR1; G6PD, glucose-6-phosphate 1-dehydrogenase; GGCT, gamma-glutamylcyclotransferase; GGT, glutathione hydrolase; GPX, glutathione peroxidase; GR, glutathione reductase; GSH1, glutamate-cysteine ligase; GSH2, glutathione synthetase; ICDH, isocitrate dehydrogenase; LAP, leucine aminopeptidase; MDHAR, monodehydroascorbate reductase; NTR, NADPH-dependent thioredoxin reductase; OXP, 5-oxoprolinase; Trx*o*, thioredoxin *o*.

Abbreviations of metabolites in Panel B: Cys, L-cysteine; Cys-Gly, cysteinylglycine; DHA, monodehydroascorbate; Glu, L-glutamate; Gly, glycine; GSH, glutathione; GSSG, glutathione disulfide; γ-Glu-AA, γ-glutamyl amino acid; γ-Glu-Cys, γ-glutamylcysteine; MDHA, dehydroascorbate; NADPH, nicotinamide adenine dinucleotide phosphate.

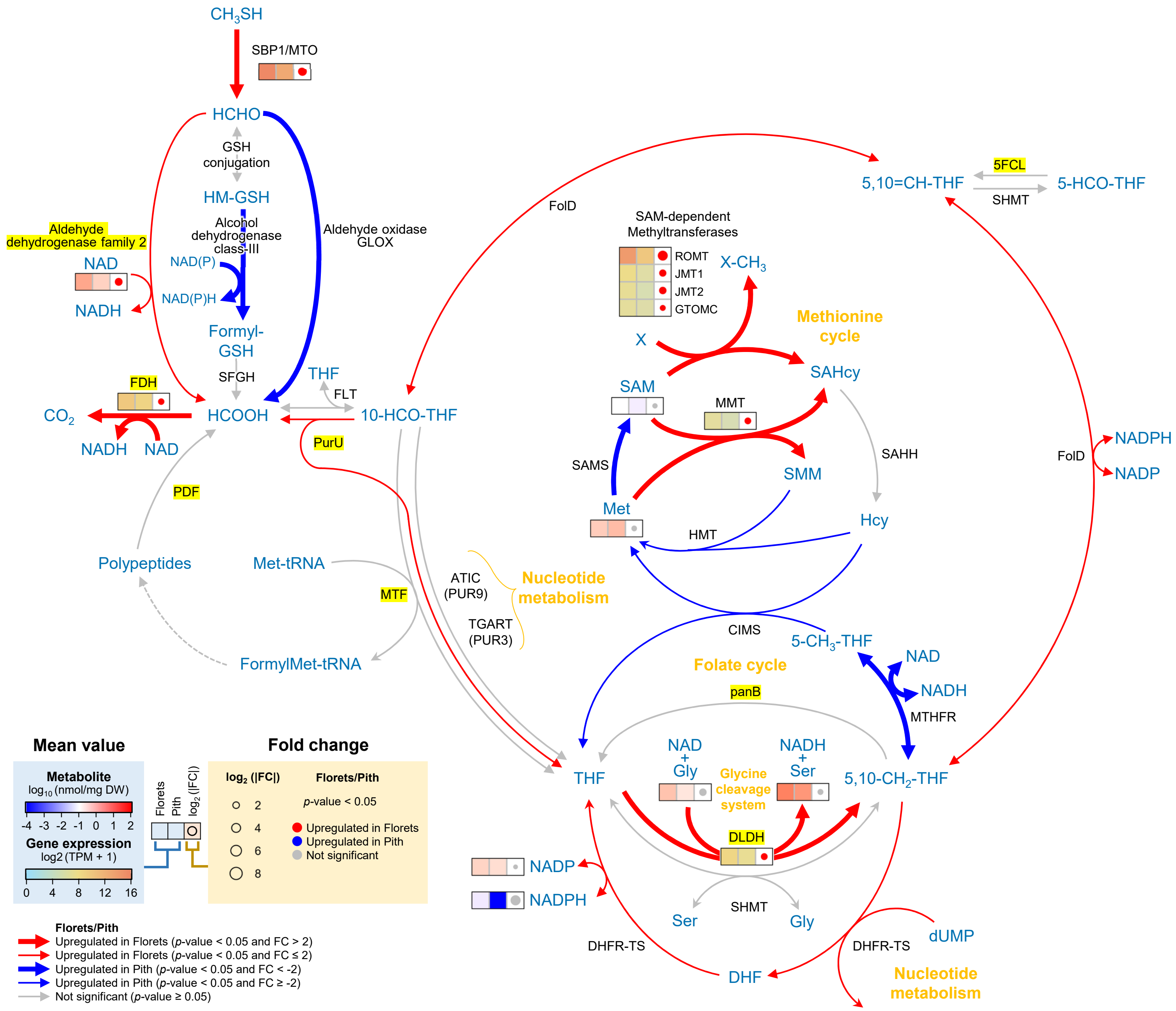

Supplemental Figure S17. Metabolic pathway of one-carbon compounds in the thermogenic florets of *S. renifolius*.
Color gradients in the lower panel show variations in metabolite accumulation ($\log_{10}$ (nmol/mg DW)) and gene expression levels ($\log_2$ (TPM) + 1). Circles of different sizes represent the $\log_2$ fold change (FC) values, ranging from 2 to 8. Filled red circles indicate upregulation in the florets, blue filled circles represent upregulation in the pith, and grey filled circles denote non-significant differences. Arrows denote upregulation trends in either the florets (red) or the pith (blue), with bold arrows indicating a *p*-value < 0.05 and FC > 2 or FC < -2. Narrow arrows show a *p*-value < 0.05 and FC ≤ 2 or FC ≥ -2, and gray arrows point to non-significant results (*p*-value ≥ 0.05). The mean values and fold changes depicted are derived from four independent samples, either from florets or pith, of thermogenic spadices. Enzymes highlighted in yellow are expected to localize to mitochondria. DW, dry weight; TPM, transcripts per million.

Supplemental Figure S17. Abbreviations:

Abbreviations of enzymes: 5FCL, 5-formyltetrahydrofolate cyclo-ligase; ATIC (PUR9), bifunctional purine biosynthesis protein PurH; CIMS, 5-methyltetrahydropteroyltriglutamate-homocysteine methyltransferase; DLDH, dihydrolipoyl dehydrogenase; DHFR-TS, bifunctional dihydrofolate reductase-thymidylate synthase; FDH, formate dehydrogenase; FLT, formate-tetrahydrofolate ligase; FolD, bifunctional protein FolD; GCSP, glycine dehydrogenase (decarboxylating); GCSH, glycine cleavage system H protein; GCST, aminomethyltransferase; GTOMC, tocopherol *O*-methyltransferase; HMT, homocysteine *S*-methyltransferase; JMT, jasmonic acid carboxyl methyltransferase; MTHFR, methylenetetrahydrofolate reductase; MMT, methionine *S*-methyltransferase; MTF, methionyl-tRNA formyltransferase; PDF, peptide deformylase; panB, 3-methyl-2-oxobutanoate hydroxymethyltransferase; PurU, formyltetrahydrofolate deformylase; ROMT, *trans*-resveratrol di-*O*-methyltransferase; SAHH, adenosylhomocysteinase; SAMS, *S*-adenosylmethionine synthase; SFGH, *S*-formylglutathione hydrolase; SHMT, serine hydroxymethyltransferase; TGART (PUR3), phosphoribosylglycinamide formyltransferase.

Abbreviations of metabolites: 10-HCO-THF, 10-formyl-tetrahydrofolate; 5,10-CH2-THF, 5,10-methylenetetrahydrofolate; 5,10=CH-THF, 5,10-methenyltetrahydrofolate; 5-CH_3-THF, 5-Methyltetrahydrofolate; 5-HCO-THF, 5-formyltetrahydrofolate; CH_3SH, methanethiol; DHF, dihydrofolate; Formyl-GSH, *S*-formylglutathione; FormylMet-tRNA, *N*-formylmethionyl-tRNA; Gly, glycine; HCHO, formaldehyde; HCOOH, formic acid; Hcy, homocysteine; HM-GSH, *S*-(hydroxymethyl)glutathione; Met, L-methionine; Met-tRNA, methionyl-tRNA; SAM, *S*-adenosylmethionine; SAHcy, *S*-adenosyl-L-homocysteine; Ser, L-serine; SMM, *S*-methylmethionine; THF, tetrahydrofolate; dTMP, thymidine monophosphate; dUMP, deoxyuridine monophosphate.

Supplemental Table S1. Thermogenic inflorescences of *S. renifolius*.

Plant ID	Habitats	*Ta* (ºC)	*Ts* (ºC)	*Ts* - *Ta* (ºC)	Thermal stage	Organ	Symbol
Pre_1	Fujine	7.8	10.2	2.4	Pre	Florets	Pre_F1
						Pith	Pre_P1
Pre_2	Fujine	6.7	11.6	4.9		Florets	Pre_F2
						Pith	Pre_P2
Pre_3	Kanegasaki	7.5	7.3	-0.2		Florets	Pre_F3
						Pith	Pre_P3
Pre_4	Kanegasaki	6.2	5.0	-1.2		Florets	Pre_F4
						Pith	Pre_P4
Hot_1	Fujine	7.4	21.8	14.4	Hot	Florets	Hot_F1
						Pith	Hot_P1
Hot_2	Fujine	8.5	22.2	13.7		Florets	Hot_F2
						Pith	Hot_P2
Hot_3	Fujine	8.6	21.5	12.9		Florets	Hot_F3
						Pith	Hot_P3
Hot_4	Fujine	8.6	20.9	12.3		Florets	Hot_F4
						Pith	Hot_P4
Post_1	Fujine	9.0	11.7	2.7	Post	Florets	Post_F1
						Pith	Post_P1
Post_2	Fujine	6.1	12.2	6.1		Florets	Post_F2
						Pith	Post_P2
Post_3	Fujine	13.1	13.5	0.4		Florets	Post_F3
						Pith	Post_P3
Post_4	Kanegasaki	3.9	8.6	4.7		Florets	Post_F4
						Pith	Post_P4

Supplemental Table S2. Concentrations of metabolites in the florets and pith across different thermogenic stages in *S. renifolius.*

Metabolite	Florets (nmol/mgDW)			Pith (nmol/mgDW)		
	Pre	Hot	Post	Pre	Hot	Post
**Sugars and sugar phosphates**						
Sucrose	51.327 ± 4.688 (c)	81.203 ± 3.416 (a)	65.306 ± 1.407 (b)	52.581 ± 2.532 (c)	73.352 ± 3.063 (a)	65.705 ± 1.950 (b)
Glucose	3.325 ± 1.878 (b)	4.931 ± 2.797 (b)	14.106 ± 3.910 (a)	16.516 ± 6.312 (a)	13.485 ± 4.270 (a)	27.275 ± 27.307 (a)
Fructose	2.980 ± 2.517 (a)	4.625 ± 4.750 (a)	14.288 ± 8.770 (a)	13.971 ± 3.905 (a)	11.189 ± 3.408 (a)	30.497 ± 30.822 (a)
Sucrose 6-phosphate	0.277 ± 0.131 (a)	0.300 ± 0.072 (a)	0.282 ± 0.099 (a)	0.070 ± 0.027 (b)	0.317 ± 0.152 (a)	0.195 ± 0.044 (ab)
UDP-glucose	1.086 ± 0.772 (b)	2.425 ± 0.246 (a)	1.514 ± 0.344 (ab)	0.432 ± 0.196 (a)	0.832 ± 0.268 (a)	0.414 ± 0.157 (a)
ADP-glucose	0.018 ± 0.024 (a)	0.024 ± 0.008 (a)	0.011 ± 0.004 (a)	0.001 ± 0.001 (a)	0.003 ± 0.002 (a)	0.003 ± 0.001 (a)
GDP-glucose	0.021 ± 0.013 (b)	0.076 ± 0.005 (a)	0.055 ± 0.029 (ab)	0.015 ± 0.002 (a)	0.021 ± 0.003 (a)	0.018 ± 0.006 (a)
Glucose 6-phosphate	1.622 ± 1.418 (b)	6.867 ± 3.143 (ab)	10.464 ± 4.438 (a)	0.716 ± 0.227 (a)	1.931 ± 0.609 (a)	1.540 ± 0.693 (a)
Fructose 1,6-bisphosphate	0.161 ± 0.190 (b)	0.844 ± 0.223 (a)	0.543 ± 0.208 (ab)	0.229 ± 0.114 (a)	0.412 ± 0.227 (a)	0.404 ± 0.241 (a)
Dihydroxyacetone phosphate	1.109 ± 0.718 (b)	2.360 ± 0.461 (a)	1.639 ± 0.582 (a)	0.211 ± 0.066 (b)	0.858 ± 0.147 (b)	0.192 ± 0.208 (a)
3-Phosphoglycerate	0.474 ± 0.414 (a)	0.746 ± 0.195 (a)	0.767 ± 0.073 (a)	0.149 ± 0.028 (b)	0.431 ± 0.114 (a)	0.430 ± 0.117 (a)
Phospho*enol* pyruvate	0.520 ± 0.217 (a)	0.428 ± 0.083 (a)	0.643 ± 0.172 (a)	0.141 ± 0.018 (b)	0.413 ± 0.094 (a)	0.388 ± 0.167 (ab)
6-Phosphogluconate	0.482 ± 0.235 (b)	2.028 ± 0.638 (ab)	11.409 ± 9.200 (a)	0.885 ± 0.038 (b)	2.482 ± 0.665 (b)	6.351 ± 1.262 (a)
Ribulose 5-phosphate	0.397 ± 0.156 (a)	0.537 ± 0.247 (a)	0.917 ± 0.614 (a)	0.442 ± 0.111 (a)	0.297 ± 0.090 (ab)	0.167 ± 0.023 (b)
Ribulose 1,5-bisphosphate	0.019 ± 0.006 (b)	0.108 ± 0.015 (a)	0.107 ± 0.058 (ab)	0.035 ± 0.007 (b)	0.081 ± 0.042 (b)	0.375 ± 0.057 (b)
Erythrose 4-phosphate	0.658 ± 0.335 (ab)	0.526 ± 0.136 (b)	1.152 ± 0.317 (a)	0.179 ± 0.024 (a)	0.350 ± 0.066 (a)	0.278 ± 0.117 (a)
Laminaribiose	0.723 ± 0.145 (b)	1.131 ± 0.285 (b)	0.817 ± 0.166 (a)	0.444 ± 0.166 (a)	0.725 ± 0.113 (b)	0.675 ± 0.049 (b)
myo-Inositol	6.374 ± 2.466 (a)	3.248 ± 0.979 (a)	5.962 ± 2.966 (a)	10.639 ± 3.645 (a)	3.375 ± 1.434 (b)	2.687 ± 1.145 (b)
Raffinose	17.283 ± 3.837 (a)	7.837 ± 3.053 (b)	3.000 ± 0.743 (b)	19.007 ± 1.547 (a)	5.691 ± 1.484 (b)	5.058 ± 1.711 (b)
Sorbitol	0.557 ± 0.149 (a)	0.485 ± 0.152 (a)	0.475 ± 0.046 (a)	0.834 ± 0.065 (a)	0.769 ± 0.167 (a)	0.635 ± 0.258 (a)
**Organic acids**						
Pyruvate	1.988 ± 0.722 (b)	4.101 ± 1.301 (b)	8.042 ± 2.846 (a)	4.818 ± 1.981 (a)	3.533 ± 0.710 (a)	6.666 ± 7.382 (a)
Acetyl-CoA	0.156 ± 0.123 (a)	0.000 ± 0.000 (b)	0.008 ± 0.016 (b)	0.195 ± 0.055 (a)	0.014 ± 0.020 (c)	0.104 ± 0.035 (b)
Citrate	13.081 ± 2.358 (a)	6.140 ± 1.614 (b)	2.103 ± 1.485 (c)	15.955 ± 0.403 (a)	14.650 ± 2.325 (a)	11.648 ± 4.807 (a)
cis-Aconitate	0.350 ± 0.064 (a)	0.390 ± 0.052 (a)	0.123 ± 0.058 (b)	0.207 ± 0.016 (b)	0.325 ± 0.026 (a)	0.242 ± 0.060 (ab)
2-Oxoglutaric acid	0.463 ± 0.257 (b)	1.700 ± 0.121 (a)	0.470 ± 0.323 (b)	0.316 ± 0.118 (b)	0.795 ± 0.279 (a)	0.390 ± 0.083 (b)
Succinate	9.693 ± 5.334 (a)	10.149 ± 1.108 (a)	4.507 ± 1.912 (a)	2.057 ± 1.211 (ab)	2.955 ± 1.489 (a)	0.705 ± 0.306 (b)
Fumarate	6.310 ± 0.917 (a)	9.882 ± 1.044 (a)	13.237 ± 7.476 (a)	9.625 ± 1.643 (b)	17.366 ± 6.024 (b)	50.643 ± 11.324 (a)
Malate	15.304 ± 3.355 (a)	26.415 ± 2.113 (a)	29.979 ± 14.823 (a)	23.843 ± 4.027 (b)	40.416 ± 11.437 (a)	80.591 ± 10.514 (b)
**Nucleotides**						
ATP	1.694 ± 1.306 (a)	1.352 ± 0.777 (a)	0.547 ± 0.214 (a)	1.741 ± 0.347 (a)	1.068 ± 0.395 (a)	1.150 ± 0.375 (a)
ADP	0.361 ± 0.211 (b)	1.007 ± 0.320 (a)	0.748 ± 0.129 (ab)	0.261 ± 0.035 (a)	0.368 ± 0.105 (a)	0.262 ± 0.087 (a)
AMP	0.508 ± 0.143 (b)	1.224 ± 0.150 (a)	0.639 ± 0.122 (b)	0.045 ± 0.021 (b)	0.227 ± 0.088 (a)	0.078 ± 0.092 (ab)
Adenosine	0.741 ± 0.109 (a)	1.720 ± 1.051 (a)	1.343 ± 0.196 (a)	0.403 ± 0.163 (a)	0.197 ± 0.146 (a)	0.172 ± 0.053 (a)
Adenine	0.285 ± 0.046 (ab)	0.290 ± 0.116 (a)	0.117 ± 0.082 (b)	0.145 ± 0.018 (a)	0.124 ± 0.030 (a)	0.101 ± 0.018 (a)
Cyclic AMP	0.005 ± 0.006 (a)	0.055 ± 0.081 (a)	0.002 ± 0.005 (a)	0.000 ± 0.000 (a)	0.000 ± 0.000 (a)	0.000 ± 0.000 (a)
IMP	0.093 ± 0.037 (b)	0.239 ± 0.024 (a)	0.111 ± 0.032 (b)	0.000 ± 0.000 (a)	0.033 ± 0.025 (a)	0.008 ± 0.016 (a)
GTP	0.127 ± 0.207 (b)	0.411 ± 0.126 (a)	0.081 ± 0.014 (b)	0.114 ± 0.054 (a)	0.097 ± 0.044 (a)	0.060 ± 0.023 (a)
GDP	0.023 ± 0.047 (b)	0.196 ± 0.058 (a)	0.090 ± 0.033 (b)	0.010 ± 0.017 (a)	0.018 ± 0.015 (a)	0.018 ± 0.009 (a)
GMP	0.009 ± 0.017 (a)	0.025 ± 0.015 (a)	0.000 ± 0.000 (a)	0.000 ± 0.000 (a)	0.001 ± 0.003 (a)	0.000 ± 0.000 (a)
Guanosine	0.089 ± 0.023 (a)	0.149 ± 0.064 (a)	0.109 ± 0.068 (a)	0.063 ± 0.016 (a)	0.049 ± 0.036 (ab)	0.004 ± 0.003 (b)
Guanine	0.090 ± 0.018 (a)	0.106 ± 0.033 (a)	0.040 ± 0.012 (b)	0.049 ± 0.010 (a)	0.057 ± 0.033 (a)	0.040 ± 0.010 (a)
UTP	0.743 ± 0.547 (a)	0.429 ± 0.248 (a)	0.087 ± 0.075 (a)	0.653 ± 0.141 (a)	0.485 ± 0.150 (ab)	0.306 ± 0.066 (b)
UDP	0.689 ± 0.131 (a)	0.485 ± 0.138 (ab)	0.295 ± 0.146 (b)	0.381 ± 0.169 (a)	0.424 ± 0.057 (a)	0.168 ± 0.030 (b)
UMP	0.338 ± 0.087 (b)	0.863 ± 0.174 (a)	0.278 ± 0.059 (b)	0.027 ± 0.017 (b)	0.301 ± 0.122 (a)	0.056 ± 0.040 (b)
Uridine	0.456 ± 0.154 (a)	0.944 ± 0.507 (a)	0.650 ± 0.193 (a)	0.199 ± 0.114 (a)	0.168 ± 0.202 (a)	0.126 ± 0.019 (a)
Uracil	0.576 ± 0.197 (a)	0.998 ± 0.546 (a)	0.763 ± 0.233 (a)	0.255 ± 0.140 (a)	0.184 ± 0.210 (a)	0.156 ± 0.028 (a)
CTP	0.278 ± 0.243 (a)	0.335 ± 0.211 (a)	0.041 ± 0.038 (a)	0.268 ± 0.079 (a)	0.138 ± 0.049 (b)	0.107 ± 0.019 (b)
CDP	0.093 ± 0.036 (a)	0.168 ± 0.065 (a)	0.087 ± 0.046 (a)	0.065 ± 0.022 (a)	0.040 ± 0.008 (ab)	0.031 ± 0.007 (b)
CMP	0.120 ± 0.057 (b)	0.440 ± 0.080 (a)	0.235 ± 0.047 (b)	0.043 ± 0.020 (a)	0.122 ± 0.095 (a)	0.054 ± 0.025 (a)
Cytidine	0.062 ± 0.026 (b)	0.183 ± 0.059 (a)	0.142 ± 0.059 (ab)	0.040 ± 0.018 (a)	0.023 ± 0.014 (a)	0.040 ± 0.007 (a)
Cytosine	0.095 ± 0.039 (a)	0.224 ± 0.089 (a)	0.187 ± 0.069 (a)	0.082 ± 0.027 (a)	0.034 ± 0.037 (a)	0.031 ± 0.005 (a)
NAD	1.910 ± 0.315 (ab)	2.295 ± 0.210 (a)	1.670 ± 0.384 (b)	0.657 ± 0.224 (a)	0.517 ± 0.078 (ab)	0.221 ± 0.212 (b)
NADP	0.322 ± 0.056 (ab)	0.475 ± 0.105 (a)	0.182 ± 0.085 (b)	0.139 ± 0.038 (b)	0.336 ± 0.042 (a)	0.080 ± 0.035 (b)
NADPH	0.008 ± 0.017 (a)	0.052 ± 0.035 (a)	0.039 ± 0.032 (a)	0.000 ± 0.000 (a)	0.000 ± 0.000 (a)	0.000 ± 0.000 (a)
**Amino acids**						
Glycine	0.539 ± 0.224 (ab)	0.882 ± 0.436 (a)	0.273 ± 0.050 (b)	0.250 ± 0.018 (a)	0.244 ± 0.086 (a)	0.147 ± 0.033 (a)
Alanine	4.229 ± 1.423 (ab)	10.572 ± 5.182 (a)	3.177 ± 2.262 (b)	3.472 ± 0.600 (a)	3.590 ± 1.628 (a)	3.496 ± 0.427 (a)
Valine	8.277 ± 2.362 (a)	10.120 ± 3.385 (a)	16.657 ± 4.190 (a)	7.221 ± 3.258 (b)	9.132 ± 1.717 (a)	16.351 ± 4.656 (ab)
Leucine	1.995 ± 0.515 (a)	3.134 ± 1.407 (a)	6.066 ± 1.411 (a)	2.191 ± 0.786 (a)	2.658 ± 0.538 (b)	5.096 ± 1.979 (ab)
Isoleucine	0.588 ± 0.325 (b)	1.495 ± 0.933 (b)	4.808 ± 1.923 (a)	0.703 ± 0.445 (b)	0.810 ± 0.240 (ab)	4.160 ± 2.021 (a)
Serine	6.409 ± 1.058 (b)	7.992 ± 2.320 (ab)	7.005 ± 1.359 (a)	4.660 ± 0.524 (a)	3.972 ± 1.756 (a)	3.435 ± 0.543 (a)
Threonine	2.998 ± 0.406 (a)	6.683 ± 0.389 (a)	10.055 ± 3.795 (a)	2.178 ± 0.350 (a)	5.151 ± 0.539 (a)	4.496 ± 2.730 (a)
Cysteine	0.089 ± 0.056 (b)	0.132 ± 0.028 (a)	0.149 ± 0.057 (b)	0.072 ± 0.040 (b)	0.059 ± 0.017 (a)	0.151 ± 0.036 (a)
Methionine	0.229 ± 0.047 (a)	0.624 ± 0.486 (a)	0.337 ± 0.061 (a)	0.299 ± 0.059 (ab)	1.121 ± 0.246 (b)	0.434 ± 0.106 (a)
Proline	15.337 ± 1.529 (a)	2.116 ± 1.137 (a)	0.913 ± 0.734 (a)	13.615 ± 1.717 (a)	1.030 ± 0.439 (a)	0.726 ± 0.122 (a)
Phenylalanine	2.326 ± 1.485 (a)	1.864 ± 0.407 (b)	3.719 ± 0.866 (b)	1.827 ± 0.534 (a)	1.388 ± 0.317 (b)	2.938 ± 1.019 (b)
Tyrosine	0.787 ± 0.372 (b)	0.750 ± 0.385 (ab)	2.391 ± 1.107 (a)	0.888 ± 0.181 (b)	0.465 ± 0.107 (b)	1.486 ± 0.940 (a)
Tryptophan	0.079 ± 0.097 (b)	0.082 ± 0.031 (b)	0.224 ± 0.187 (a)	0.042 ± 0.027 (a)	0.030 ± 0.011 (a)	0.047 ± 0.015 (a)
Asparagine	10.524 ± 1.699 (b)	17.864 ± 1.177 (a)	17.116 ± 3.317 (b)	7.885 ± 2.900 (c)	13.063 ± 0.979 (b)	16.216 ± 1.616 (a)
Glutamine	25.787 ± 3.254 (b)	13.949 ± 3.286 (b)	21.163 ± 6.540 (a)	22.539 ± 2.355 (a)	19.964 ± 4.410 (a)	19.841 ± 2.159 (a)
Aspartate	5.077 ± 1.371 (a)	14.499 ± 1.248 (a)	7.380 ± 4.531 (a)	7.231 ± 1.445 (b)	15.188 ± 2.153 (b)	19.957 ± 1.682 (a)
Glutamate	8.663 ± 2.816 (a)	33.085 ± 7.358 (b)	11.755 ± 7.838 (ab)	10.130 ± 1.553 (a)	22.885 ± 2.508 (a)	26.873 ± 6.190 (a)
Histidine	1.072 ± 0.344 (b)	1.094 ± 0.391 (b)	4.868 ± 2.234 (a)	1.173 ± 0.402 (b)	0.675 ± 0.196 (b)	1.141 ± 0.329 (a)
Arginine	1.590 ± 1.358 (b)	0.712 ± 0.196 (a)	13.046 ± 8.474 (a)	32.743 ± 4.726 (b)	0.531 ± 0.141 (a)	1.750 ± 1.274 (a)
Lysine	2.559 ± 0.501 (a)	2.200 ± 0.861 (a)	4.255 ± 2.086 (a)	5.130 ± 1.499 (b)	2.091 ± 0.224 (b)	3.204 ± 1.726 (a)
4-Hydroxyproline	0.026 ± 0.017 (a)	0.087 ± 0.036 (a)	0.058 ± 0.045 (a)	0.024 ± 0.004 (a)	0.020 ± 0.005 (a)	0.034 ± 0.025 (a)
Phosphoserine	0.006 ± 0.006 (b)	0.050 ± 0.006 (a)	0.036 ± 0.026 (ab)	0.006 ± 0.002 (a)	0.106 ± 0.069 (a)	0.215 ± 0.176 (a)
**Polyamines and others**						
Argininosuccinate (ASA)	0.027 ± 0.015 (b)	0.120 ± 0.068 (a)	0.053 ± 0.021 (ab)	0.014 ± 0.005 (b)	0.033 ± 0.003 (a)	0.024 ± 0.008 (ab)
Ornithine	0.157 ± 0.048 (b)	0.108 ± 0.054 (b)	0.749 ± 0.512 (a)	0.305 ± 0.068 (a)	0.155 ± 0.068 (a)	0.759 ± 0.894 (a)
N-Acetylglutamate	0.070 ± 0.041 (a)	0.103 ± 0.027 (a)	0.284 ± 0.235 (a)	0.035 ± 0.012 (b)	0.168 ± 0.038 (ab)	0.264 ± 0.151 (a)
Agmatine	0.027 ± 0.013 (a)	0.008 ± 0.004 (b)	0.016 ± 0.006 (ab)	0.018 ± 0.002 (a)	0.005 ± 0.002 (c)	0.012 ± 0.003 (b)
Citrulline	0.049 ± 0.033 (a)	0.058 ± 0.017 (a)	0.459 ± 0.496 (a)	0.075 ± 0.013 (ab)	0.045 ± 0.013 (b)	0.266 ± 0.168 (a)
S-Adenosylmethionine	0.115 ± 0.024 (a)	0.100 ± 0.024 (a)	0.087 ± 0.008 (a)	0.077 ± 0.029 (a)	0.060 ± 0.026 (ab)	0.022 ± 0.014 (b)
Spermidine	0.713 ± 0.464 (a)	0.058 ± 0.016 (b)	0.194 ± 0.088 (ab)	1.345 ± 0.330 (a)	0.738 ± 0.429 (a)	0.873 ± 0.354 (a)
Spermine	0.010 ± 0.008 (a)	0.000 ± 0.000 (b)	0.000 ± 0.000 (b)	0.028 ± 0.010 (a)	0.036 ± 0.037 (a)	0.025 ± 0.027 (a)
N-acetyl spermidine	0.137 ± 0.264 (a)	0.004 ± 0.001 (a)	0.006 ± 0.002 (a)	0.222 ± 0.375 (a)	0.013 ± 0.005 (a)	0.018 ± 0.018 (a)
Glutathione	10.805 ± 1.335 (a)	12.082 ± 3.930 (a)	9.377 ± 2.133 (a)	6.275 ± 2.754 (a)	6.775 ± 1.312 (a)	5.709 ± 2.319 (a)
Glutamylcysteine	0.058 ± 0.021 (a)	0.138 ± 0.060 (a)	0.205 ± 0.176 (a)	0.046 ± 0.005 (a)	0.077 ± 0.031 (a)	0.358 ± 0.519 (a)
Glycyl-glycine	10.524 ± 1.699 (b)	17.864 ± 1.177 (a)	17.116 ± 3.317 (a)	7.885 ± 2.900 (b)	13.063 ± 0.979 (b)	16.216 ± 1.616 (a)
Gamma-Aminobutyric acid	1.070 ± 0.346 (a)	2.262 ± 1.723 (a)	1.629 ± 0.571 (a)	0.827 ± 0.390 (a)	0.698 ± 0.788 (a)	1.911 ± 1.596 (a)
Ascorbate	12.061 ± 1.804 (b)	20.077 ± 4.367 (a)	2.651 ± 4.300 (c)	0.000 ± 0.000 (a)	1.743 ± 3.485 (a)	14.740 ± 16.544 (a)
Cinnamate	0.241 ± 0.078 (a)	0.407 ± 0.160 (a)	0.201 ± 0.109 (a)	0.284 ± 0.049 (a)	0.424 ± 0.093 (a)	0.371 ± 0.044 (a)
Glucuronate	1.073 ± 0.233 (a)	0.619 ± 0.154 (b)	0.283 ± 0.131 (b)	0.351 ± 0.198 (a)	0.201 ± 0.103 (a)	0.280 ± 0.113 (a)
Glycolate	1.332 ± 0.424 (a)	1.477 ± 0.244 (a)	1.243 ± 0.319 (a)	1.475 ± 0.275 (a)	1.169 ± 0.241 (a)	1.154 ± 0.793 (a)
Tyramine	0.019 ± 0.012 (b)	0.060 ± 0.025 (b)	0.361 ± 0.085 (a)	0.092 ± 0.021 (b)	0.487 ± 0.264 (ab)	4.117 ± 2.950 (a)

All data are expressed as mean ± standard deviation (n = 4 except for Pre_pith n = 3). Statistically significant differences are indicated by different letters, as determined by a Tukey-Kramer test (p < 0.05).

Supplemental Table S3. Tissue- and thermogenic stage-specific statistical analysis of metabolite accumulation in *S. renifolius*.

Metabolite	Fold change (Florets/Pith)			*p*-value from *t*-test (Florets/Pith)		
	Pre	Hot	Post	Pre	Hot	Post
**Sugars and sugar phosphates**						
Sucrose	-1.02	-1.11	-1.01	0.77309	0.04856	0.82260
Glucose	-4.97	-2.73	-1.93	0.08186	0.05112	0.50204
Fructose	-4.69	-2.42	-2.13	0.06184	0.10934	0.48872
Sucrose 6-phosphate	3.94	-1.06	-1.45	0.15002	0.86545	0.25846
UDP-glucose	2.51	2.92	3.66	0.39355	0.00228	0.01119
ADP-glucose	15.69	7.71	4.02	0.44470	0.01484	0.02087
GDP-glucose	-1.44	3.66	3.14	0.59900	0.00018	0.10284
Glucose 6-phosphate	2.27	3.56	6.80	0.51832	0.06266	0.02597
Fructose 1,6-bisphosphate	-1.43	2.05	-1.34	0.76192	0.07366	0.54848
Dihydroxyacetone phosphate	5.27	2.75	8.43	0.21655	0.00713	0.01562
3-Phosphoglycerate	3.18	-1.73	-1.78	0.41845	0.07305	0.01338
Phospho*enol* pyruvate	3.68	-1.04	-1.66	0.13220	0.84855	0.14537
6-Phosphogluconate	-1.84	-1.22	-1.80	0.13515	0.41632	0.45690
Ribulose 5-phosphate	-1.11	-1.81	5.50	0.77309	0.16926	0.10780
Ribulose 1,5-bisphosphate	-1.92	-1.34	-3.49	0.10354	0.31257	0.00885
Erythrose 4-phosphate	3.68	-1.50	4.14	0.17039	0.10306	0.01196
Laminaribiose	-1.63	-1.56	-1.21	0.17039	0.07893	0.25241
myo-Inositol	-1.67	-1.04	2.22	0.26196	0.88827	0.15648
Raffinose	-1.10	-1.38	-1.69	0.65863	0.30573	0.13316
Sorbitol	-1.50	-1.59	-1.34	0.13220	0.08451	0.39580
**Organic acids**						
Pyruvate	-2.42	-1.16	-1.21	0.14729	0.52871	0.82260
Acetyl-CoA	-1.25	-5.67	-10.28	0.76192	0.28240	0.01338
Citrate	-1.22	-2.39	-5.54	0.23082	0.00713	0.02967
cis-Aconitate	-1.69	-1.20	-1.98	0.10182	0.10938	0.07201
2-Oxoglutaric acid	-1.46	2.14	-1.23	0.59900	0.00713	0.72940
Succinate	4.71	3.43	6.39	0.17039	0.00376	0.02637
Fumarate	-1.53	-1.76	-3.83	0.10354	0.08928	0.01147
Malate	-1.56	-1.53	-2.69	0.12879	0.09253	0.01147
**Nucleotides**						
ATP	-1.03	-1.27	-2.10	0.95459	0.59696	0.07842
ADP	-1.38	2.74	2.85	0.61469	0.03333	0.00905
AMP	11.26	5.40	8.19	0.04407	0.00061	0.00885
Adenosine	-1.84	8.74	7.80	0.10673	0.07305	0.00235
Adenine	-1.96	2.33	-1.16	0.05656	0.07305	0.81980
Cyclic AMP	6.83	65.90	3.49	0.39355	0.28240	0.48872
IMP	14.88	6.85	8.86	0.08350	0.00049	0.01119
GTP	-1.12	4.23	-1.35	0.93833	0.01727	0.26697
GDP	2.28	10.68	4.96	0.77088	0.00713	0.02151
GMP	9.30	12.33	1.00	0.59900	0.06960	1.00000
Guanosine	-1.40	3.05	25.72	0.32808	0.07366	0.06070
Guanine	-1.82	-1.85	-1.00	0.10354	0.12824	0.99724
UTP	-1.14	-1.13	-3.51	0.84164	0.75034	0.01947
UDP	-1.81	-1.14	-1.75	0.14679	0.50631	0.23894
UMP	12.48	2.87	4.98	0.04279	0.01185	0.00905
Uridine	2.30	5.63	5.16	0.17039	0.07305	0.01162
Uracil	2.26	5.42	4.90	0.17039	0.07305	0.01196
CTP	-1.04	2.44	-2.64	0.95459	0.16926	0.06009
CDP	-1.43	4.21	2.84	0.47099	0.03111	0.10780
CMP	2.81	3.60	4.33	0.19980	0.01295	0.00885
Cytidine	-1.56	7.98	3.55	0.43641	0.01185	0.04280
Cytosine	-1.17	6.61	5.95	0.76192	0.03111	0.01782
NAD	2.91	4.44	7.55	0.04279	0.00019	0.00885
NADP	2.31	-1.41	2.28	0.05656	0.08749	0.13096
NADPH	3.80	18.92	14.02	0.59900	0.07305	0.12608
**Amino acids**						
Glycine	2.15	3.62	-1.86	0.20530	0.07305	0.02151
Alanine	-1.22	2.95	-1.10	0.59900	0.08128	0.85566
Valine	-1.15	-1.11	-1.02	0.76192	0.66410	0.96734
Leucine	-1.10	-1.18	-1.19	0.77309	0.60187	0.58952
Isoleucine	-1.20	-1.84	-1.16	0.77309	0.26130	0.76744
Serine	-1.38	2.01	2.04	0.15109	0.07305	0.01338
Threonine	-1.38	-1.30	2.24	0.14278	0.01789	0.11478
Cysteine	-1.23	2.24	-1.01	0.77309	0.01793	0.99588
Methionine	-1.31	-1.80	-1.29	0.29323	0.16926	0.26697
Proline	-1.13	2.05	-1.26	0.39355	0.17130	0.75837
Phenylalanine	-1.27	-1.34	-1.27	0.76192	0.16926	0.42011
Tyrosine	-1.13	-1.61	-1.61	0.77309	0.26130	0.39030
Tryptophan	-1.90	2.75	4.79	0.71152	0.05829	0.19086
Asparagine	-1.33	-1.37	-1.06	0.36875	0.00713	0.75837
Glutamine	-1.14	-1.43	-1.07	0.39355	0.11438	0.81980
Aspartate	-1.42	-1.05	-2.70	0.23320	0.64881	0.01196
Glutamate	-1.17	-1.45	-2.29	0.61469	0.07956	0.06270
Histidine	-1.09	-1.62	4.27	0.78731	0.15982	0.04862
Arginine	-20.59	-1.34	7.46	0.00453	0.24031	0.09375
Lysine	-2.00	-1.05	-1.33	0.10673	0.84855	0.59692
4-Hydroxyproline	-1.09	4.35	-1.73	0.88493	0.03727	0.50204
Phosphoserine	-1.07	-2.12	-5.98	0.93833	0.21021	0.16500
**Polyamines and others**						
Argininosuccinate (ASA)	-1.90	3.62	2.20	0.41343	0.08128	0.09578
Ornithine	-1.95	-1.43	-1.01	0.10354	0.37638	0.99588
N-Acetylglutamate	2.00	-1.63	-1.07	0.39355	0.07305	0.94375
Agmatine	-1.45	-1.62	-1.28	0.51832	0.28261	0.47194
Citrulline	-1.55	-1.27	-1.73	0.43219	0.35747	0.61462
S-Adenosylmethionine	-1.50	-1.67	4.03	0.25461	0.10632	0.00774
Spermidine	-1.89	-12.75	-4.50	0.23376	0.05845	0.03101
Spermine	-2.88	-139.04	-1874.12	0.14852	0.15982	0.20059
N-acetyl spermidine	-1.62	-3.43	-3.09	0.78731	0.03111	0.33432
Glutathione	-1.72	-1.78	-1.64	0.13220	0.08128	0.12023
Glutamylcysteine	-1.26	-1.78	-1.74	0.59900	0.17114	0.73464
Glycyl-glycine	-1.33	-1.37	-1.06	0.36875	0.00713	0.75837
Gamma-Aminobutyric acid	-1.29	3.24	-1.17	0.59900	0.20196	0.82260
Ascorbate	168.63	11.18	-5.56	0.00453	0.00713	0.32274
Cinnamate	-1.18	-1.04	-1.85	0.59900	0.86931	0.07201
Glucuronate	3.05	3.08	-1.01	0.07150	0.01793	0.99588
Glycolate	-1.11	-1.26	-1.08	0.76192	0.17114	0.90023
Tyramine	-4.75	-8.13	-11.39	0.04279	0.05777	0.10062

Fold change was calculated by dividing the concentration in florets by pith (n = 4 except for Pre_pith n = 3). The *p*-values from *t*-tests were corrected by false discovery rate. All statistical hypothesis testing was performed in MetaboAnalyst 5.0.

Supplemental Table S4. Selenium contents in various organs of *S. renifolius*.

Tissue	Se content (mg/kg fresh weight)
Spadix	<0.1
Spathe	<0.1
Leaf	<0.1
Rhizome	<0.1

Supplemental Table S5. Sequences of the primers used for RT-qPCR.

Name	Gene	Sequence (5'- 3')
rt_L36_FW_001	*SBP1*	TTGCCTTGGAGACAAAGATG
rt_L36_RV_002	*SBP1*	GGTTGGTACCAGAAATCAC
rtEF1aF1	*EF1α*	AGCATTGTGGTCATTGG
rtEF1aR1	*EF1α*	CTCTTGTTCATCTCAGCAG
RT_SrAOX_F1	*AOX*	AAGGAGATCGACAACGGGACCATC
RT_SrAOX_R1	*AOX*	CTGGTAATGGATGTCCGAGGCAAAG
L360_F3	*PFK*	GCCAGGAACGGTGATCATCT
L360_R1	*PFK*	TGGGTACGGAGATGGGGAAT
L293_F5	*FBPase*	CTGCGTGGAAGGTACTGTGT
L293_R5	*FBPase*	CACAAGCGTGCAAGAACTCC
L2790_F1	*PFPβ*	CGTCATCTGCGGCATCTTTG
L2790_R1	*PFPβ*	ATCTTGTCCCTTCCACTGCG
