# Supplementary material for: Gene expression and metabolite levels converge in the thermogenic spadix of skunk cabbage
Source: Plant Physiol. 2024 Feb 6;195(2):1561–85. doi: 10.1093/plphys/kiae059 (PMC11142342; doi:10.1093/plphys/kiae059)
Supplement: kiae059_Supplementary_Data [file pp.01320.2023-s10.pdf › pp.01320.2023-s10.pdf]

Plant Physiology®

A Convergence of Gene and Metabolite Expression in the Thermogenic Spadix of Skunk Cabbage, *Symplocarpus renifolius*

Haruka Tanimoto, Yui Umekawa, Hideyuki Takahashi, Kota Goto, and Kikukatsu Ito

Review Timeline:

Submission Date:	29-Aug-2023
Editorial Decision:	08-Oct-2023
Revision Received:	30-Nov-2023
Editorial Decision:	18-Dec-2023
Revision Received:	11-Jan-2024
Accepted:	11-Jan-2024

08-Oct-2023

Prof. Kikukatsu Ito
Faculty of Agriculture, Iwate University
Morioka, Iwate 020-8550
Japan

RE: A Convergence of Gene and Metabolite Expression in the Thermogenic Spadix of Skunk Cabbage, *Symplocarpus renifolius*

Dear Dr. Ito:

We have received reviews from three experts in the field of plant thermogenesis and respiratory metabolism, which you may view at the end of this letter. On the basis of the advice received and my own evaluation, I believe that, pending revision, this article may ultimately be acceptable for publication in Plant Physiology.

You will see the excitement by all three reviewers about this comprehensive study, and I agree with them. Based on the fact that your work provides important new clues into the molecular regulation of thermogenesis and the heat production process, I believe it is firmly within the scope of Plant Physiology, even though not all clues (e.g. H2S-mediated regulation) are yet substantiated.

The three reviewers provide instructive and detailed advise to guide the revision. Those points deserve your specific attention and should be addressed where possible. I would like to add that I think that no further experimentation will be needed to address the raised points. Yet, specific datasets will require to be analysed further and sections of the text will need to be re-developed. Specifically, the introduction and discussion sections will benefit from being linked more tightly to previous work, that deserves to be discussed. Examples that were raised during the discussion with the reviewers:
Umekawa et al. (2016) proposed a mechanism for temperature regulation in skunk cabbage based on Le Chatelier's Principle, which does not depend on gene expression. Reference to Seymour et al. (2010) on respiration rates of thermogenic flowers clamped at desired experimental temperatures to clarify the difference between thermoregulation and thermogenesis.

When you are ready to submit your revision, click the below link and find the red arrow under your Author Tasks heading for this revision submission.

If you want to visit this version of the manuscript, you may click the link below and then click on the folder that reads "Post Decision Manuscripts".

Link Not Available

When uploading the revised version of this article, please be sure to include as complete an answer as possible to the referees' criticisms during the first round of reviews in the "response to reviewers" section.

Plant Physiology considers a revision for up to 60 days after a manuscript has been provisionally accepted; limited extension of this timeframe is at the discretion of the Monitoring Editor. A reminder notice will be sent to you 2 weeks before the deadline, at which time you may choose to contact the editorial office to request an extension; if so, please provide a justification for your request. After the expiration of this deadline, the article would need to be submitted as a resubmission and would be handled as such.

Thank you very much for giving us an opportunity to review this work. I look forward to receiving the next version.

Sincerely,
Plant Physiology Board of Editors

Want to add this revision deadline to your calendar? Click below!

-- Reviewer comments:
Reviewer #1 (Comments for the Author):

In the present manuscript - A Convergence of Gene and Metabolite Expression in the Thermogenic Spadix of Skunk Cabbage, Symplocarpus renifolius - the authors combined transcriptome and metabolome to get insights on the regulation of thermogenesis. This manuscript holds promise for advancing our understanding of plant thermogenesis. A particularly intriguing discovery is the inhibition of COX respiration by H2S, while AOX remains unaffected. The experiments were well-executed, and the data clearly demonstrate that the samplings and stage separations clustered together. However, I urge the authors to further explore their data and construct hypotheses and models that elucidate the regulation of thermogenesis. While the results are described effectively, there's room to delve deeper by hypothesizing how each finding contributes to thermogenesis regulation. Additionally, it would be valuable for the authors to identify and discuss the primary changes occurring during thermogenesis, moving beyond enriched pathways, such as metabolism. To align with the standards of Plant Physiology, it's advisable for the authors to conduct a more thorough exploration of their data, providing not only descriptive insights into different stages but also hypotheses connecting the key players in thermogenesis. I have outlined several major points I would appreciate if the authors addressed.

Regarding the text:

157-160: The authors mention that the temperature is increased in florets compared to the pith. However, it's unclear if the pith is also thermogenic. Please clarify this in the text.

Figure 1B: The text should clarify whether the spadix picture shows a cross-section to demonstrate thermogenesis in both pith and florets or just in one of them. Make it clear whether both pith and florets are thermogenic.

205-207: Confirm whether the pith is thermogenic or not, as this seems to be unclear in the text. Explain the rationale for analyzing both pith and florets.

Figure 1C: Specify whether "Ta" represents air temperature or spadix temperature. Explain why it varies in different stages and clarify the duration over which sampling was conducted.

Figure 1D: Consider removing Figure 1D if it doesn't provide informative content.

Figure 2A Analysis and Comparisons: The analysis and comparisons depicted in Figure 2A require more detailed explanation to ensure clarity:

Specify the nature of the comparisons: It's important to outline how the analysis was conducted and what specific comparisons were made. Clarify whether each stage of the florets was compared to a non-thermogenic stage of florets or to a non-thermogenic stage of pith.

Rationale for chosen comparisons: Elaborate on the rationale behind choosing these particular comparisons. If the authors compared florets to non-thermogenic pith, explain the scientific reasoning behind this choice.

Inclusion of pith in the analysis: Address the apparent contradiction in the text where pith is described as non-thermogenic, yet differentially expressed genes (DEGs) for pith are included in all three stages of analysis. Provide a clear explanation for the inclusion of pith in the analysis.

Temperature data for pith and florets: Specify the temperatures at which pith and florets were harvested. Confirm whether these temperatures were indeed the same and, if not, explain any variations observed.

Focus on up-regulated genes: Explain why the authors chose to primarily showcase up-regulated genes and did not include information about down-regulated genes. Provide the rationale for this decision.

Interpretation of the volcano plot: Clarify the interpretation of the volcano plot. Explain what is represented on the left side (DEGs for pith) and on the right side (DEGs for florets) and how the comparison between these sets of genes was conducted.

Address apparent bias in data plotting: Address the concern that the way the data are plotted may give the impression that all DEGs in pith are down-regulated and in florets are up-regulated. Provide a detailed explanation of the distribution of up and down-regulated genes in both tissues.

Details on data input and statistical analysis: Explain in intricate detail the data that were used as input to create the volcano plots and the statistical methods employed in the analysis. Provide transparency about how these plots were generated and how statistical significance was determined.

By addressing these points, the authors can significantly enhance the clarity and transparency of the analysis presented in Figure 2A.

Figure 2B: Specify what comparisons are being made in Figure 2B.

Figure 2C: Explain whether piths are thermogenic and describe their contribution to thermogenesis. Ensure consistency in temperature data between pith and florets.

Other Points:

237-238: If "hot pith" is thermogenic, provide a clear explanation of why the analysis includes both florets and pith.

Figure 3: Describe Figure 3 in more detail, and consider moving relevant content from Figure S1 to the main figure. Discuss both up-regulated and down-regulated genes.

Figure 4: Mention the genes that are strictly up and down-regulated during the thermogenic phase for clarity.

Line 329 and Analysis of Gene Expression in Florets:

In Line 329, where the authors showcase the top 10 genes with the highest expression in thermogenic florets, there are some potential improvements to enhance clarity and provide a more comprehensive analysis:

Clarify Terminology: To avoid potential confusion, consider changing the term "thermogenic florets" to simply "florets." While it's true that thermogenic florets are the ones producing heat, using the term "florets" is more inclusive and accurate.

Additional Analysis: Highlight the importance of certain genes showing differences in expression during thermogenesis and note that some genes exhibit reduced expression during this process. Provide a more in-depth analysis of these genes and their potential significance in the context of thermogenesis.

Comparison with Pre and Post Hot Stages: Suggest conducting additional analyses to better understand the gene expression changes during thermogenesis. Plotting both up-regulated and down-regulated genes during thermogenesis compared to stages before and after can provide valuable insights. This comparison would shed light on the major changes that occur during thermogenesis in contrast to the stages before and after.

Enhance Panel B: Panel B currently appears to depict similar information as Panel A. To maximize the information conveyed, consider including more differentially expressed genes (DEGs) in Panel B, specifically in a comparison with the stages before and after thermogenesis. This approach would offer a deeper understanding of the key alterations occurring during thermogenesis.

By implementing these suggestions, the authors can improve the clarity of their analysis and provide a more detailed exploration of gene expression dynamics during thermogenesis in florets.

Supplemental Figure S3 is being shown before Supplemental Figure S2

Figure 6: Explain the choice of comparisons in Figure 6A and why a p-value of 0.1 was used. Suggest to show Figure 6B first. It's important to provide an explanation for why florets were compared to pith rather than to other florets. Clarify the scientific rationale behind this choice and why it was considered more meaningful for the study.

Reorganization of Figures:
Suggest reorganizing the figures as follows for better flow and clarity:
Figure 6B to be placed before Figure 6A.
Figure 6A to be followed by Figure 6C.
Figure 7 could be incorporated as Figure 6B.
Continue with Figure 6C, followed by Figure 6D.
Figure 6D to be succeeded by Figure 6E.
Finish with Figure 6E leading into Figure 6F.

Combination of Figures:

Suggest merging Figures 6, 7, and 8 into one single figure to consolidate related information and make it more comprehensible. This combined figure can provide a comprehensive overview of the main changes observed between stages, streamlining the

presentation of results.
By implementing these suggestions, the authors can enhance the clarity and coherence of their figures and better communicate the main findings and changes between different stages of the study.

Figure 9: Define "energy charge" and explain its calculation. Discuss why AMP changed but not ATP in Figure 9B.

Figure 10: Clarify how data were plotted in Figure 10, including whether it represents averages or data from the hot stage only.

Figures 11 and H2S Measurement: Explain the rationale for comparing pith with florets and provide information on H2S levels in non-thermogenic and thermogenic florets.

Figure 11C: Describe the thermogenic stage at which mitochondria were isolated and provide details on the temperature changes during mitochondrial respiration. Include information on H2S concentrations and explain the rationale for using them.

Figure 12: Clarify which figures correspond to what in the results section and decide whether Figure 12B is necessary.

Material and Methods Section:

In the Materials and Methods section, provide detailed explanations of how each analysis was performed, the specific comparisons made, the statistical methods used, and the DEG identification process.
It's crucial to address these issues in the manuscript to ensure clarity and transparency in the research findings. You may need to revise the text and figures accordingly. Number of replicates should also be added to the legend of each figure.

Introduction and discussion: Suggest inclusion of more work done with thermogenic plants specially regarding metabolism. In the introduction and discussion metabolism and previous studies were superficially described.

Reviewer #2 (Comments for the Author):

General Comments

This well-written paper describes a powerhouse of results from analyses of the transcriptome and metabolome in relation to stage of development in a thermogenic plant. There were no less than a dozen techniques involved in measures of gene expression and biochemical metabolites apparent in three stages of floral development in a flower that show intense self-heating (thermogenesis) and biochemical temperature regulation. The results are quite detailed and complex yet are clearly described with numerous attractive figures and tables. The summary figure (Fig 12C) illustrates the major findings of the study that lead to fascinating novel hypotheses for the control of heat production.

Major revisions

1. Thermogenesis means heat-production, a feature of all respiring organisms including plants and animals. In this context, thermogenesis in plant flowers is high enough to raise the floral temperature significantly above the ambient temperature. Some plant species show a degree of thermoregulation, which is floral temperature being somewhat constant and independent of ambient temperature. So, thermogenesis and thermoregulation are not unique to Asian skunk cabbage. I suggest clarifying the distinction between the two terms early in the paper, because the study addresses the 'development' of thermogenic capacity (involving pre, hot and post thermogenic stages) and the phenomenon of thermoregulation (involving respiration at 15, 23 and 30C tissue temperatures where respiration rate decreases at progressively higher temperatures). Without this distinction a reader may become confused later in the paper.

For example, and as a suggestion, the first two sentences of the Abstract could be revised something like this:
The inflorescence of the Asian skunk cabbage, Symplocarpus renifolius, is strongly thermogenic and can regulate its temperature at around 23{degree sign}C even when the ambient temperature drops below freezing. Thermogenesis and thermoregulation are unexpected phenomena in plants. To elucidate the mechanisms underlying developmentally-controlled ability for thermogenesis, and to understand the mechanism of thermoregulation, we conducted a comprehensive analysis of the transcriptome and metabolome across three developmental stages of the inflorescence.

2. (lines 635-659) This section suggests that a 'futile cycle' occurs between F6P and FBP, which could result in heat production without oxygen. The evidence is higher expression of the genes for PFK and FBPase in the florets (Fig. S4). However, there is

little support for this process in thermogenic species. The study of Newsholme et al. (1972) proposed that the high levels of fructose bisphosphtase activity in the flight muscles of bumblebees might be used to keep the flight muscle warm while resting between flights between flowers. However, they did not show that this was true. A later study by Bernd Heinrich showed that heat production in resting bumblebees never occurred without action potentials in flight muscle, indicating that muscle cycling was essential. The study by Staples et al. (2004), cited by the authors, also does not support significant thermogenesis by the futile cycle in bumblebees. In other thermogenic flowers, there is good matching of heat production measured by direct calorimetry and oxygen consumption, suggesting that the futile cycle does not produce significant heat. I suggest that this section weakens the paper.
(A minor point is that the availability of oxygen is not diminished under snow unless there is a solid layer of ice. The porosity of snow is generally between 0.4 to 0.98, which is higher than sand or soils. Not only is oxygen diffusion in snow very high, the large amount of oxygen in snow would not inhibit respiration.)

3. (lines 697-705) This is an interesting discovery that seems very important. However, it is not clear how lower tissue temperature stimulates SBP1/MTO to produce more hydrogen sulphide. This is the key idea here, since HS shifts respiration away from energy-conserving COX to heat-liberating AOX. The respirometry results in Figure 11C and D do show the effect of HS on the ratios of AOX/COX, however, the absolute respiration rates of both pathways do not show the highest values at 15C and lowest at 30C as occurs in the intact skunk cabbage spadices. Also, the rates of both pathways decrease with higher HS concentrations. Evidently this has something to do with measurements from isolated mitochondria without their cellular milieu. These things are worth discussing.

Minor revisions

157 What is meant by 'a higher temperature distribution'? Is 'distribution' necessary?
179 and 182 It is not clear what 'this study' and 'that study' refer to.
233 Please define what 'small molecule processes' are.
237 'Conversely' does not seem appropriate here.
263 Please say what the insights are.
279-281 It appears to me that Group 2 reveals the largest change between pre-thermogenic and thermogenic stages. However, this group might not receive the attention that it deserves. (It does return later in the paper (325), but maybe it should be flagged earlier.
295-312 What is the functional origin of the titles for these groups? It is difficult for the non-expert to see how they relate to the groups 1-6.

Reviewer #3 (Comments for the Author):

The study by Tanimoto et al. uses a combined transcriptomic and metabolomic approach to investigate potential mechanisms and regulation of thermogenesis in skunk cabbage. Plant thermogenesis is a very interesting and rare phenomenon, limited to a small subset of species. It is, however, of extreme importance to these species, typically in the promotion of pollination by various means, including pollen development and/or the attraction of pollinators. Additionally, the pathway typically associated with plant thermogenesis, alternative respiration, is also vitally important for managing plant stress responses even in non-thermogenic species, therefore broadening the target audience.
The 'omics approach taken here reconfirms old players in skunk cabbage thermogenesis, including the alternative oxidase gene, but importantly also uncovers new potential contributors. Some new candidates included orthologs of known metabolic proteins such as phosphfructokinase and fructose-1,6-bisphosphatase, suggesting a role for futile metabolic cycles in promoting thermogenesis, in addition to electron leakage via alternative respiration. Other candidates included nucleotide metabolism proteins, which could explain altered energy status of the tissue, including a decrease in ATP:AMP during thermogenesis. Sucrose transporter SWEET6 was up-regulated specifically at the thermogenic stage, suggesting that carbohydrate supply may be involved in regulating the initiation of thermogenesis.
One gene candidate in particular, is quite novel and interesting. The authors denoted this gene as SrSBP1/MTO. It was the most highly expressed gene in the thermogenic floret tissue. A protein potentially encoded by this gene was detected by immunoblotting using newly designed antibodies. The authors then purified and partially sequenced this protein from the same tissue to characterise key amino acid residues. They conclude that SBP1/MTO is likely involved in the generation of hydrogen sulfide, which was also shown to accumulate in the thermogenic floret tissue. The authors also show that H_2S more effectively inhibits COX compared to AOX, thus high H_2S levels may promote alternative respiration within mitochondria purified from floret tissue. Furthermore, at colder temperatures, H_2S was more effective at inhibiting COX, therefore favouring AOX and promoting thermogenesis. At warmer temperatures, H_2S did not inhibit COX as effectively. This study has therefore uncovered a potential new player in regulation of mitochondrial respiration, which could explain the ability of this plant to not only carry out floral thermogenesis, but thermoregulation in response to fluctuating ambient temperature. This will likely inform future studies on the regulation of alternative respiration, both in thermogenic and non-thermogenic plants.

Below are minor comments/questions relating to specific aspects of the article, in order of appearance.
1. L266 - could a similar analysis be done for pith samples? Do any of the transcript or metabolite changes in the pith confirm a

role for this tissue during thermogenesis, e.g. supplying respiratory substrates to the florets?
2. L380-383 - interesting that alanine levels are high in florets - this could be a source of mitochondrial pyruvate, thus activating AOX (Le et al. 2022, doi: 10.1038/s41477-022-01165-3)
3. L453-457 - clarify that this was experimentally validated at the transcript level, not protein nor activity. PFK1 and F1,6BPase activities are regulated by levels of F2,6BP - are there any data on F2,6BP or the transcript abundance of the enzymes involved in its metabolism, PFK2 / F2,6BPase?
4. L616-618 - Zheng et al 2022 also suggested importance of SWEET in sugar transport for thermogenesis.
5. L620-624 - would be good to see an immunoblot to determine if pre-thermogenic transcription of AOX leads to accumulation of the protein at this stage. Also, pyruvate activation has not been mentioned. Could also comment on how the alternative dehydrogenases, e.g. NDA1 from Group 1, might be involved. Looks like an NDB transcript is also up in thermogenic floret (Fig 10).
6. L694-697 - and in animals, see Bremer et al. 2021. doi: 10.1242/jeb.242985
7. L690-705 - is it possible that the activity of SBP1/MTO (and therefore H2S concentration) is also temperature-regulated?
8. L726-727 - consider whether increased operation of alternative respiration pathway would also lead to a decrease in ATP levels relative to ADP or AMP.
9. L935-945 - why was mannitol used as osmoticum in mitochondrial grinding and wash media, but sucrose in the percoll and assay solutions? Were mitochondria still reasonably intact after freeze-thawing for respiratory activity measurements?
10. Fig 2A-C - are DEGs calculated as florets relative to pith? It seems so, based on methods, but should explicitly state this in legend.
11. Figs 3A and 7 - Pre_P1 missing from metabolomics clustering analysis (Fig 7) but Pre_P4 missing from DEG clustering analysis (Fig 3A). Why were different replicates excluded?
12. Figure 10 - clarify which data were used - is it only from thermogenic stage as stated in L447-448? Because the legend states it is across different thermogenesis stages - if so, were the data from different thermogenic stages averaged together?
13. Figure 11 C,D - when measured in the presence of inhibitors, AOX and COX should be reported as "capacities" rather than "activities". Same at L945-950.
14. Figure S1 - why are some conditions not reported, e.g. Group 6 Hot? Also check order of graphs from Groups 5 and 6.

Manuscript ID: PP2023-RA-01320
Title: A Convergence of Gene and Metabolite Expression in the Thermogenic Spadix of Skunk Cabbage, *Symplocarpus renifolius*
Authors: Haruka Tanimoto, Yui Umekawa, Hideyuki Takahashi, Kota Goto, and Kikukatsu Ito

Monitoring Editor

Comments to the Author:

The three reviewers provide instructive and detailed advise to guide the revision. Those points deserve your specific attention and should be addressed where possible. I would like to add that I think that no further experimentation will be needed to address the raised points. Yet, specific datasets will require to be analysed further and sections of the text will need to be re-developed. Specifically, the introduction and discussion sections will benefit from being linked more tightly to previous work, that deserves to be discussed. Examples that were raised during the discussion with the reviewers: Umekawa et al. (2016) proposed a mechanism for temperature regulation in skunk cabbage based on Le Chatelier's Principle, which does not depend on gene expression. Reference to Seymour et al. (2010) on respiration rates of thermogenic flowers clamped at desired experimental temperatures to clarify the difference between thermoregulation and thermogenesis.

The revised MS has the revisions indicated in red type.

Responses from the authors (in blue italic type).

--
We thank the editors for offering us the opportunity to revise and resubmit this paper. In response to the invaluable comments and suggestions from the reviewers, we have made an extensive revision to this paper. In our revised manuscript, we have clearly clarified the difference between thermogenesis and thermoregulation in both the Introduction and Discussion sections, incorporating the thermal clamping experiments detailed in Seymour et al. (2010), and our previous study on thermodynamic analyses of thermoregulation by Umekawa et

al., (2016). Moreover, our transcriptomic and metabolomic analytical processes are now thoroughly outlined, accompanied by comprehensive figures and panels that present detailed data for both the florets and pith tissues. Additional datasets have been included as separate Supplemental Data files. We believe these revisions have significantly strengthened our manuscript, making it a strong candidate for publication in Plant Physiology. Our detailed responses to the comments of the three reviewers are provided below.

Referee(s)' Comments to Author:

Reviewer #1 Manuscript Review...

In the present manuscript - A Convergence of Gene and Metabolite Expression in the Thermogenic Spadix of Skunk Cabbage, Symplocarpus renifolius - the authors combined transcriptome and metabolome to get insights on the regulation of thermogenesis. This manuscript holds promise for advancing our understanding of plant thermogenesis. A particularly intriguing discovery is the inhibition of COX respiration by H2S, while AOX remains unaffected. The experiments were well-executed, and the data clearly demonstrate that the samplings and stage separations clustered together. However, I urge the authors to further explore their data and construct hypotheses and models that elucidate the regulation of thermogenesis. While the results are described effectively, there's room to delve deeper by hypothesizing how each finding contributes to thermogenesis regulation. Additionally, it would be valuable for the authors to identify and discuss the primary changes occurring during thermogenesis, moving beyond enriched pathways, such as metabolism. To align with the standards of Plant Physiology, it's advisable for the authors to conduct a more thorough exploration of their data, providing not only descriptive insights into different stages but also hypotheses connecting the key players in thermogenesis. I have outlined several major points I would appreciate if the authors addressed.

Firstly, we would like to express our sincere gratitude to Reviewer #1 for its constructive and insightful comments on our manuscript. We address each of the points raised by Reviewer #1 as shown below.

Regarding the text:

157-160: The authors mention that the temperature is increased in florets compared to the pith. However, it's unclear if the pith is also thermogenic. Please clarify this in the text.

In the Introduction, we explain the structural features of the spadix, the thermogenic organ of S. renifolius. We refer to our previous thermoscopic analysis, which indicates a temperature difference between the florets and the pith:

"The spadix of S. renifolius is composed of florets and pith. Our previous thermoscopic analyses of the thermogenic stage of spadices revealed that the florets, located on the outer parts of the spadix, maintain higher temperatures compared to the inner pith tissue (Onda et al., 2008). Thus, it has been suggested that the thermogenesis predominantly occurs in the florets, while the pith does not directly contribute to this process in the spadix (Onda et al., 2008)." (p5, ls 99-104)

Additionally, we elaborate on the expression of AOX, which is significantly higher in the florets than in the pith tissue during the thermogenic stage:

"In S. renifolius, AOX transcripts and proteins are remarkably abundant in the thermogenic spadix (Onda et al., 2007). Moreover, the expression levels of AOX transcripts are specifically found in the florets, particularly the petal and pistil (Sayed et al., 2016). Given the tissue-specific expression of the AOX in the petal and pistil, it is probable that these tissues in the florets play a significant role in developmentally controlled thermogenesis in S. renifolius, while the pith, does not directly contribute to this process." (p7, ls 177-183)

In the Results section, we clarify the roles of the florets and pith in thermogenesis by providing detailed explanations as shown below:

"*It is important to note that the florets, located in the outer part of the spadix, are the primary tissues for thermogenesis, whereas the inner pith generates less heat (Onda et al., 2008), and is thus referred to as non-thermogenic tissue in*

this study." (p10, ls 228-231)

We believe that these modifications and clarifications in our revised manuscript will facilitate a better understanding of the significance of utilizing both the florets and pith for our series of comparative transcriptomic, metabolomic, and biochemical analyses.

Figure 1B: The text should clarify whether the spadix picture shows a cross-section to demonstrate thermogenesis in both pith and florets or just in one of them. Make it clear whether both pith and florets are thermogenic.

We have revised the figure caption to provide a clearer explanation. The updated caption for Figure 1B now reads:

"(B) Images showing the thermogenic stage of intact S. renifolius (upper left), a thermal image capturing the surface temperature (lower left), and a longitudinal section indicating the positions of florets and pith (right)."

205-207: Confirm whether the pith is thermogenic or not, as this seems to be unclear in the text. Explain the rationale for analyzing both pith and florets.

As previously mentioned, we have provided a clearer explanation regarding the role of the pith, along with the rationale for analyzing both pith and florets in our study.

Figure 1C: Specify whether "Ta" represents air temperature or spadix temperature. Explain why it varies in different stages and clarify the duration over which sampling was conducted.

We have modified the caption to clearly specify the terms used in the figure. The updated caption now reads:

"(C) Comparative data for air temperature (Ta), spadix temperature (Ts), and their difference (Ts - Ta)."

Both air temperature (Ta) and spadix temperature (Ts) across different stages is

reflective of the dynamic nature of thermogenesis during the developmental stages of S. renifolius.

Figure 1D: Consider removing Figure 1D if it doesn't provide informative content.

We respectfully propose retaining Figure 1D in our manuscript, as it serves as a succinct presentation of the current study's samples and offers essential information on their characteristics.

The caption for Figure 1D has been carefully crafted to provide detailed explanations, particularly regarding the empty boxes in the 'Pre_1' and 'Pre_4' samples. These denote instances where samples were not subjected to CE-MS or RNA-seq analysis due to the insufficient tissue quantity, a result of the small size of the pith tissues at the pre-thermogenic spadices. This additional information in the caption further clarifies our sampling methodology and the constraints we encountered, thereby enriching the readers' understanding of our study's design and execution.

“Figure 1D. Details of the analyzed samples, indicated by labels corresponding to the thermogenic stages of the spadix in S. renifolius. Stages are labeled as 'Pre_x' for pre-thermogenic, 'Hot_x' for thermogenic, and 'Post_x' for post-thermogenic samples, where 'x' denotes the specific sample number. Each label also includes information on the spadix temperature (Ts), the sampling site (either Fujine (F) or Kanegasaki (K)), and the analysis method used (RNA-seq or CE-MS). Empty boxes in 'Pre_1' and 'Pre_4' denote samples that were not subjected to CE-MS or RNA-seq analysis due to insufficient tissue quantity resulting from the small size of the pith-tissues at the pre-thermogenic spadices.”

Figure 2A. Analysis and Comparisons: The analysis and comparisons depicted in Figure 2A require more detailed explanation to ensure clarity:

Specify the nature of the comparisons: It's important to outline how the analysis was conducted and what specific comparisons were made. Clarify whether

each stage of the florets was compared to a non-thermogenic stage of florets or to a non-thermogenic stage of pith.

In our revised manuscript, we have added the Supplemental Figure S1, which provides an overview of the trans-omics analyses conducted in our study. This addition helps to contextualize the overall approach and methodology employed in our research. Furthermore, to address the specific nature of the comparisons, we have included the Supplemental Figure S2. This figure explicitly shows that the comparisons were made between the florets and the pith within the same thermogenic stage of spadix.

Rationale for chosen comparisons: Elaborate on the rationale behind choosing these particular comparisons. If the authors compared florets to non-thermogenic pith, explain the scientific reasoning behind this choice.

As mentioned in our previous response, we have provided a detailed explanation for the comparisons chosen in our study. To reiterate, the rationale behind comparing florets to pith within the same thermogenic stage is to elucidate the distinct roles these tissues play in the thermogenesis of S. renifolius. This comparative approach allows us to highlight the differential thermogenic activity and molecular profiles between the florets, which are actively thermogenic, and the pith, which exhibits less thermogenic activity. By focusing on these specific comparisons, our study aims to provide a comprehensive understanding of the localized mechanisms of thermogenesis within the plant. We believe that the supplemental figures added, as mentioned earlier, further clarify and support the scientific reasoning behind our choice of comparisons.

Inclusion of pith in the analysis: Address the apparent contradiction in the text where pith is described as non-thermogenic, yet differentially expressed genes (DEGs) for pith are included in all three stages of analysis. Provide a clear explanation for the inclusion of pith in the analysis.

In our initial version, our analysis focused predominantly on the florets, but in the revised manuscript, we performed k-means clustering of DEGs and grouping based on expression patterns in both florets and pith, with specific

gene names listed for each group (shown in Figure 3C, 3D, 3E, and Supplemental Figure S3). We have also extended the GO analysis related to Figure 3C to include the pith (Supplemental Figure S4). Furthermore, in Figure 4, we present the top 10 DEGs in the pith alongside those in the florets. We have supplemented the manuscript text with explanations pertaining to these related results. The rationale for including the pith in our analysis, as stated earlier, is to elucidate its role, albeit non-thermogenic, in the overall thermogenic process of S. renifolius. This inclusion allows for a more comprehensive understanding of the plant's thermogenic mechanism by comparing active (florets) and less active (pith) tissues.

Temperature data for pith and florets: Specify the temperatures at which pith and florets were harvested. Confirm whether these temperatures were indeed the same and, if not, explain any variations observed.

We have detailed the temperature measurement methods in the Materials and Methods section of our revised manuscript as shown below.

"Both the spadix and ambient temperatures at the study sites were measured using a portable digital thermometer (TR-52; T&D Corporation, Nagano, Japan) The sensor probe was inserted with minimal tissue disruption, as previously described (Ito et al., 2004; Onda et al., 2008)." (p32, ls 863-866)

In our study, we assumed that the internal temperatures of intact spadices of S. renifolius are uniformly distributed. It is noteworthy that when a spadix is cut open for a longitudinal section and exposed to external air, temperature differences between the florets and the pith can be observed using an infrared camera. This is depicted in a video presented by Onda et al., 2008, in Plant Physiology. The video (available at https://academic.oup.com/plphys/article/146/2/323/6107264) demonstrates that prior to cutting, the temperatures of florets and pith are the same, but as time progresses post-cutting, a temperature difference emerges. This observation recorded in our research aligns with the understanding that intact spadices maintain a uniform temperature distribution, while exposure to external conditions post-cutting leads to temperature variances.

For the purposes of our study, we considered the temperatures at which the florets and pith were sampled to be effectively identical, given that the spadices remained intact. This approach ensures the validity of our comparative analysis and is consistent with observations in the field of plant thermogenesis research.

Focus on up-regulated genes: Explain why the authors chose to primarily showcase up-regulated genes and did not include information about down-regulated genes. Provide the rationale for this decision.

In addressing the focus on up-regulated genes in our study, it is important to clarify that our analysis, particularly when comparing florets to pith, considers genes that are highly expressed in florets as up-regulated, and those highly expressed in pith as down-regulated. This approach is showcased in various parts of our analysis, including Figure 2A.

By categorizing the genes in this manner, we aimed to emphasize the differential expression patterns that are most relevant to the thermogenic process in S. renifolius. The up-regulated genes in the florets typically represent the active thermogenic mechanisms, whereas the down-regulated genes, or those more highly expressed in the pith, offer insights into the non-thermogenic or less active thermogenic processes. This distinction is crucial for understanding the contrasting roles of these tissues in the plant's overall thermogenesis.

We believe this approach provides a comprehensive view of gene expression dynamics in S. renifolius, highlighting the specific contributions of each tissue type to the thermogenic phenomenon.

Interpretation of the volcano plot: Clarify the interpretation of the volcano plot. Explain what is represented on the left side (DEGs for pith) and on the right side (DEGs for florets) and how the comparison between these sets of genes was conducted.

For clarification on the interpretation of the volcano plot in Figure 2, we have modified the figure's caption to provide a more detailed explanation. In the revised caption, we explicitly indicate that the DEGs for florets are represented

in red, while those for pith are shown in blue on the volcano plot.

“Figure 2A. Volcano plots highlighting the DEGs between the florets and pith at various thermogenic stages. Red dots represent DEGs in florets, while blue dots indicate DEGs in the pith.”

This visualization allows for a straightforward comparison between the sets of genes differentially expressed in the florets and pith, thereby providing insights into the distinct molecular mechanisms underpinning thermogenesis in these two tissues. We believe this enhanced explanation in the figure caption will aid in a clearer understanding of how the DEGs for both florets and pith are represented and interpreted in our study.

Moreover, to provide a clear understanding of the process leading to the identification of the DEGs, we have displayed this process in Supplemental Figure S2. This supplemental figure details the process we employed in identifying and categorizing the DEGs in our study.

Furthermore, to provide a comprehensive understanding of the statistical analysis employed in our study, we have included detailed descriptions in the Materials and Methods section of our revised manuscript.

“Differentially Expressed Genes (DEGs) were identified through an organ-wise comparison between the florets and the pith of the spadix at various thermogenic stages. We conducted a quantitative analysis using four biological replicates, except for the pre-thermogenic stage pith samples, where only three replicates were used. The Strand NGS statistical replicate analysis tool was employed for statistical analysis, operating under Benjamini-Hochberg-corrected moderated t-test conditions. This approach adjusts for small sample sizes to improve the estimation of gene variances (Benjamini and Hochberg, 1995; Smyth, 2004). We considered gene expression significant if the p-value was less than 0.05 and the absolute fold change exceeded 2.0. Unless otherwise stated, all parameters adhered to the software's default settings. For the identified DEGs, we counted the number of gene overlaps between thermogenic stages in both florets and pith, and subsequently drew a Venn diagram.” (p33, ls 907-p34, ls 919)

Address apparent bias in data plotting: Address the concern that the way the data are plotted may give the impression that all DEGs in pith are down-regulated and in florets are up-regulated. Provide a detailed explanation of the distribution of up and down-regulated genes in both tissues.

In our organ-wise comparisons between the florets and pith, genes that are statistically significantly more highly expressed in the florets are defined as upregulated, and this definition serves as the criterion for identifying DEGs. In other words, the DEGs identified in the florets are all genes that are upregulated compared to those in the pith tissue.

To address any potential misinterpretation regarding the distribution of up- and down-regulated genes in both florets and pith, we have made efforts to clearly explain this in the manuscript and Supplemental Figure S2A, ensuring that the distribution of up- and down-regulated genes in both tissues is accurately represented and understood.

Details on data input and statistical analysis: Explain in intricate detail the data that were used as input to create the volcano plots and the statistical methods employed in the analysis. Provide transparency about how these plots were generated and how statistical significance was determined.

We have presented diagrams showing the in silico analysis procedure in Supplemental Figure S1 and Supplemental Figure S2. These figures aim to provide a visual overview of the analytical workflow and the steps involved in processing the data.

Additionally, we have presented the TPM normalized count data obtained in our study (Supplemental Data S1-S3). These data sets, which form the basis of our statistical analysis, are crucial for understanding the gene expression dynamics observed. The lists of genes identified before and after the statistical analysis are explicitly shown in Supplemental Data S1-S3. This comprehensive presentation of our data and the corresponding gene lists is intended to provide a clear view of the input used for generating the volcano plots.

Furthermore, we have included detailed descriptions of the statistical methods employed in our analysis in the Materials and Methods section of the manuscript. This includes the criteria for determining statistical significance, the specific tests used, and how these were applied to our data. By providing these intricate details, we aim to ensure that the process of generating the volcano plots and determining statistical significance is fully transparent and understandable.

We believe these measures collectively enhance the comprehensibility of our statistical approach and the reliability of the findings presented in our study.

Figure 2B: Specify what comparisons are being made in Figure 2B.

Regarding the Figure 2B, we wish to clarify that the Venn diagram represents the distribution of DEGs in either the florets or the pith. This diagram is critical for understanding the genes that are expressed in either of these tissues across various thermogenic stages.

In the revised manuscript, we have moved the Venn diagram to Supplemental Figure S2B. Along with this diagram, we have also presented the workflow for the identification of DEGs. This relocation and the inclusion of the workflow aim to provide a clearer context for the interpretation of the Venn diagram. It shows the overlaps and distinctions in gene expression between the florets and pith at various stages, offering a comprehensive view of the DEG distribution in these tissues.

Figure 2C: Explain whether piths are thermogenic and describe their contribution to thermogenesis. Ensure consistency in temperature data between pith and florets.

In our revised manuscript, we have described the role that the pith of S. renifolius plays in the thermogenic phenomenon, focusing on the gene expression patterns identified in this tissue.

"Analysis of the pith identified several genes with noteworthy expression patterns. For instance, in Group P1b, sucrose synthase 2 is notable for its

expression during the pre-thermogenic stage. The presence of genes encoding heat shock 70kDa proteins 1 and 2 in Group P2 indicates that the increased temperature induces temperature stress in the non-thermogenic pith tissues. Notably, within Group P3, the presence of genes coding for cysteine protease RD21A highlights their potential role in the post-thermogenic pith tissues. For Groups P4 to P6, YTH domain-containing protein ECT2 (Group P4), defensin-like protein P322 (Group P5), and glycine-rich RNA binding protein GRP1A (Group P6) were found (Figure 3E)." (p14, ls 353-362)

Other Points:

237-238: If "hot pith" is thermogenic, provide a clear explanation of why the analysis includes both florets and pith.

Already been addressed in the responses above.

Figure 3: Describe Figure 3 in more detail, and consider moving relevant content from Figure S1 to the main figure. Discuss both up-regulated and down-regulated genes.

We have moved the results of the HCA to Figure 2B and revised Figure 3 to focus on interpreting the results of non-hierarchical clustering (k-means). Also, we mentioned the functions of representative genes categorized in each group.

Figure 4: Mention the genes that are strictly up and down-regulated during the thermogenic phase for clarity.

In the comparison of florets and pith, genes that are statistically significantly more expressed in florets are defined as upregulated, and vice versa for downregulated genes.

Line 329 and Analysis of Gene Expression in Florets:

In Line 329, where the authors showcase the top 10 genes with the highest expression in thermogenic florets, there are some potential improvements to enhance clarity and provide a more comprehensive analysis:

Clarify Terminology: To avoid potential confusion, consider changing the term "thermogenic florets" to simply "florets." While it's true that thermogenic florets are the ones producing heat, using the term "florets" is more inclusive and accurate.

Throughout the manuscript, we have consistently referred to florets during the thermogenic phase as 'thermogenic florets.' For stages without heat-production, we have used terms like 'pre-thermogenic' to indicate the presence or absence of heat-production.

Additional Analysis: Highlight the importance of certain genes showing differences in expression during thermogenesis and note that some genes exhibit reduced expression during this process. Provide a more in-depth analysis of these genes and their potential significance in the context of thermogenesis.

Comparison with Pre and Post Hot Stages: Suggest conducting additional analyses to better understand the gene expression changes during thermogenesis. Plotting both up-regulated and down-regulated genes during thermogenesis compared to stages before and after can provide valuable insights. This comparison would shed light on the major changes that occur during thermogenesis in contrast to the stages before and after.

In our revised manuscript, we already show groups of genes whose expression levels vary with the changes in the thermogenic stage (Figure 3A). Moreover, this includes genes that are induced when the spadix temperature rises and those whose expression decreases during thermogenesis (Figure 3B and 3C). Figures 3D and 3E provide important information for considering the relationship between gene expression and thermogenesis in S. renifolius.

Enhance Panel B: Panel B currently appears to depict similar information as Panel A. To maximize the information conveyed, consider including more differentially expressed genes (DEGs) in Panel B, specifically in a comparison with the stages before and after thermogenesis. This approach would offer a deeper understanding of the key alterations occurring during thermogenesis.

In our previous manuscript, Figure 5B showed a heatmap of the top 10 highly expressed genes in hot florets. In the revised version, this figure has been removed and replaced with k-means clustering and related figures (Figures 3D and 3E), allowing for comparison of gene expression patterns of major genes before, during, and after thermogenesis.

By implementing these suggestions, the authors can improve the clarity of their analysis and provide a more detailed exploration of gene expression dynamics during thermogenesis in florets.
Supplemental Figure S3 is being shown before Supplemental Figure S2

Revised accordingly.

Figure 6: Explain the choice of comparisons in Figure 6A and why a p-value of 0.1 was used. Suggest to show Figure 6B first. It's important to provide an explanation for why florets were compared to pith rather than to other florets. Clarify the scientific rationale behind this choice and why it was considered more meaningful for the study.

To maintain consistency on all statistical hypothesis testing conducted in our revised manuscript, we have changed the threshold for expression variation in metabolome analysis to a p-value < 0.05, identical to transcriptome analysis. This change is reflected in the current Figures 5, 7, Supplemental Figures S6, S8, S14, and S15. The rationale for comparing florets and pith has already been addressed.

Reorganization of Figures:
Suggest reorganizing the figures as follows for better flow and clarity:
Figure 6B to be placed before Figure 6A.
Figure 6A to be followed by Figure 6C.
Figure 7 could be incorporated as Figure 6B.
Continue with Figure 6C, followed by Figure 6D.
Figure 6D to be succeeded by Figure 6E.
Finish with Figure 6E leading into Figure 6F.

Combination of Figures:

Suggest merging Figures 6, 7, and 8 into one single figure to consolidate related information and make it more comprehensible. This combined figure can provide a comprehensive overview of the main changes observed between stages, streamlining the presentation of results.
By implementing these suggestions, the authors can enhance the clarity and coherence of their figures and better communicate the main findings and changes between different stages of the study.

We have restructured the three figures related to metabolome analysis and integrated them into a new Figure 5. Additionally, the volcano plot has been moved to Supplemental Figure S6.

Figure 9: Define "energy charge" and explain its calculation. Discuss why AMP changed but not ATP in Figure 9B.

We have included the formula for calculating energy charge in the caption of Figure 6. Although ATP levels tend to remain constant as a result of cellular homeostasis, our manuscript discusses the possibility of decreased ATP levels when the activity of AOX increases.

"Furthermore, the increased capacities of the mitochondrial alternative respiration pathway in the florets of thermogenic spadices (Onda et al., 2007), also contribute to the decreased ATP levels, which in turn lead to an increased level of AMP." (p29, ls 794-797)

Figure 10: Clarify how data were plotted in Figure 10, including whether it represents averages or data from the hot stage only.

The results of the pathway analysis presented in Figure 7, Supplemental Figures S8, S14, and S15 compare expression levels in the florets and pith during the thermogenic stage. This comparison is clearly indicated in each figure.

Figures 11 and H2S Measurement: Explain the rationale for comparing pith with

florets and provide information on H2S levels in non-thermogenic and thermogenic florets.

In Figure 8A, measuring hydrogen sulfide levels in both tissues (i.e., the florets and pith in the thermogenic stage of spadices) is important for understanding the relationship between SBP1/MTO expression and hydrogen sulfide production.

"We hypothesized that SBP1/MTO, which is specifically expressed within the florets of thermogenic spadices, plays a role in the production of hydrogen sulfide, a byproduct catalyzed by MTO. To test this hypothesis, we compared hydrogen sulfide concentrations in both the florets and pith tissues from the thermogenic stage of spadices." (p20, ls 552-556)

Figure 11C: Describe the thermogenic stage at which mitochondria were isolated and provide details on the temperature changes during mitochondrial respiration. Include information on H2S concentrations and explain the rationale for using them.

The mitochondria used for respiration measurement were all isolated from the florets at the thermogenic spadices. The indicated concentrations of hydrogen sulfide were externally added to each assay.

"Mitochondria were purified from the florets at the thermogenic stage of the spadices using previously described methods with minor modifications (Umekawa et al., 2016; Umekawa and Ito, 2019)." (p37, ls 1027-1029)

Figure 12: Clarify which figures correspond to what in the results section and decide whether Figure 12B is necessary.

The convergence model of gene and metabolite expression depicted in Figure 12 (now Figure 9 in the revised version) is a novel and facilitates a comprehensive understanding that thermogenesis is not achieved by a single factor, such as AOX alone. The sources of each data set are clearly indicated in the caption for the revised Figure 9.

Material and Methods Section:

In the Materials and Methods section, provide detailed explanations of how each analysis was performed, the specific comparisons made, the statistical methods used, and the DEG identification process.
It's crucial to address these issues in the manuscript to ensure clarity and transparency in the research findings. You may need to revise the text and figures accordingly. Number of replicates should also be added to the legend of each figure.

As described above, we have added figures to help understanding the overall analysis (Supplemental Figures S1 and S2). Where samples had replicates, the number of samples is indicated in the captions (e.g., n=4). Additionally, the Materials and Methods section details the statistical processing of gene and metabolite expression data.

Introduction and discussion: Suggest inclusion of more work done with thermogenic plants specially regarding metabolism. In the introduction and discussion metabolism and previous studies were superficially described.

The Introduction and Discussion sections have been revised to include more detailed studies on thermogenic and non-thermogenic plants, with the incorporation of new references, as shown in the text and references.

Reviewer #2 Manuscript Review...

General Comments

This well-written paper describes a powerhouse of results from analyses of the transcriptome and metabolome in relation to stage of development in a thermogenic plant. There were no less than a dozen techniques involved in measures of gene expression and biochemical metabolites apparent in three stages of floral development in a flower that show intense self-heating (thermogenesis) and biochemical temperature regulation. The results are quite detailed and complex yet are clearly described with numerous attractive figures and tables. The summary figure (Fig 12C) illustrates the major findings of the study that lead to fascinating novel hypotheses for the control of heat production.

First of all, we would like to express our sincere gratitude for the reviewer's advice and valuable comments. They have been quite helpful in revising our manuscript.

Major revisions

1. Thermogenesis means heat-production, a feature of all respiring organisms including plants and animals. In this context, thermogenesis in plant flowers is high enough to raise the floral temperature significantly above the ambient temperature. Some plant species show a degree of thermoregulation, which is floral temperature being somewhat constant and independent of ambient temperature. So, thermogenesis and thermoregulation are not unique to Asian skunk cabbage. I suggest clarifying the distinction between the two terms early in the paper, because the study addresses the 'development' of thermogenic capacity (involving pre, hot and post thermogenic stages) and the phenomenon of thermoregulation (involving respiration at 15, 23 and 30C tissue temperatures where respiration rate decreases at progressively higher temperatures). Without this distinction a reader may become confused later in the paper.

For example, and as a suggestion, the first two sentences of the Abstract could be revised something like this:

The inflorescence of the Asian skunk cabbage, Symplocarpus renifolius, is strongly thermogenic and can regulate its temperature at around 23{degree sign}C even when the ambient temperature drops below freezing. Thermogenesis and thermoregulation are unexpected phenomena in plants. To elucidate the mechanisms underlying developmentally-controlled ability for thermogenesis, and to understand the mechanism of thermoregulation, we conducted a comprehensive analysis of the transcriptome and metabolome across three developmental stages of the inflorescence.

We have changed two places in the Abstract to clarify the distinction between thermogenesis and thermoregulation. The revised parts are as follows:

"The inflorescence of the Asian skunk cabbage, Symplocarpus renifolius, is strongly thermogenic and can regulate its temperature at around 23°C even when the ambient temperature drops below freezing. Thermogenesis and thermoregulation are unexpected phenomena in plants. To elucidate the mechanisms underlying developmentally controlled ability for thermogenesis, and to understand the mechanism of thermoregulation, we conducted a comprehensive analysis of the transcriptome and metabolome across three developmental stages of the inflorescence." (p2, ls 28-35)

"This synchrony may play a key role in the developmentally regulated thermogenesis. Moreover, such convergence during the thermogenic stage in the spadix may provide a solid molecular basis for thermoregulation in S. renifolius." (p2, ls 50-53)

We have also clearly stated the difference between thermogenesis and thermoregulation in the Introduction section. The revised parts are as follows:

"In S. renifolius, the spadix also exhibits a unique phenomenon known as 'thermoregulation,' maintaining a relatively stable internal temperature of approximately 23°C, regardless of external air temperature fluctuations. The distinction between thermogenesis and thermoregulation can be further elucidated through thermal clamping of the flowers of thermogenic plants (Seymour et al., 2010). In thermoregulatory S. renifolius, a decrease in tissue temperature leads to an increase in respiration rate, a response not generally

observed in flowers exhibiting only thermogenesis. Significantly, the spadices of S. renifolius exhibit their maximum respiration rate at 15°C, defining the lower limit of their thermoregulatory range that spans from 15°C to 30°C. Our previous study demonstrated that thermoregulation in S. renifolius is mediated through a biochemical equilibrium, characterized by an overall activation energy displaying a negative value within its thermoregulatory temperature range (Umekawa et al., 2016).” (p4, ls 84-p5, ls 97)

2. (lines 635-659) This section suggests that a 'futile cycle' occurs between F6P and FBP, which could result in heat production without oxygen. The evidence is higher expression of the genes for PFK and FBPase in the florets (Fig. S4). However, there is little support for this process in thermogenic species. The study of Newsholme et al. (1972) proposed that the high levels of fructose bisphosphtase activity in the flight muscles of bumblebees might be used to keep the flight muscle warm while resting between flights between flowers. However, they did not show that this was true. A later study by Bernd Heinrich showed that heat production in resting bumblebees never occurred without action potentials in flight muscle, indicating that muscle cycling was essential. The study by Staples et al. (2004), cited by the authors, also does not support significant thermogenesis by the futile cycle in bumblebees. In other thermogenic flowers, there is good matching of heat production measured by direct calorimetry and oxygen consumption, suggesting that the futile cycle does not produce significant heat. I suggest that this section weakens the paper.
(A minor point is that the availability of oxygen is not diminished under snow unless there is a solid layer of ice. The porosity of snow is generally between 0.4 to 0.98, which is higher than sand or soils. Not only is oxygen diffusion in snow very high, the large amount of oxygen in snow would not inhibit respiration.)

We have removed our explanations to the futile cycle from the Abstract and Discussion. The role of this pathway in the thermogenesis of S. renifolius is unclear at this stage, but its potential importance for future research is noted in the Results section:

*“*Nevertheless, to fully understand the biological implications of the futile cycle in thermogenic florets, further research is needed to elucidate its specific

biochemical functions and physiological roles in *S. renifolius*." (p18, ls 498- p19, ls 501)

3. (lines 697-705) This is an interesting discovery that seems very important. However, it is not clear how lower tissue temperature stimulates SBP1/MTO to produce more hydrogen sulphide. This is the key idea here, since HS shifts respiration away from energy-conserving COX to heat-liberating AOX. The respirometry results in Figure 11C and D do show the effect of HS on the ratios of AOX/COX, however, the absolute respiration rates of both pathways do not show the highest values at 15C and lowest at 30C as occurs in the intact skunk cabbage spadices. Also, the rates of both pathways decrease with higher HS concentrations. Evidently this has something to do with measurements from isolated mitochondria without their cellular milieu. These things are worth discussing.

We have discussed the possibility that data obtained from experiments using isolated mitochondria may shift the equilibrium point of the biochemical reactions that we previously proposed:

"Although the respiration analysis utilizing isolated mitochondria may not fully represent the actual cellular conditions within the florets of thermogenic spadices, the findings of this study offer a significant understanding of the mechanism of thermoregulation in S. renifolius. Specifically, observations regarding the intersection point of AOX- and COX-mediated respiration under varying concentrations of hydrogen sulfide (Figure 8C) demonstrate a dynamic interplay among temperature, hydrogen sulfide levels, and mitochondrial respiration. In our previous study, which employed thermodynamic analysis on intact spadices of S. renifolius, we demonstrated that thermoregulation in this species is governed by a negative activation energy that involves biochemical pre-equilibrium reactions, including a rate-determining reaction catalyzed by the mitochondrial terminal oxidases AOX and COX (Umekawa et al., 2016). Therefore, changes in the equilibrium point of the biochemical reactions for thermogenesis, which utilize already established gene expressions, are pivotal in thermoregulation. Thus, our current study further suggests that hydrogen sulfide, the product of SBP1/MTO, can act as a novel effector in altering the biochemical equilibrium point, thereby controlling cellular respiration and

contributing to thermoregulation in the spadices of S. renifolius." (p28, ls 757-774)

Minor revisions

157 What is meant by 'a higher temperature distribution'? Is 'distribution' necessary?

We have made changes to clarify the explanation.

"Our previous thermoscopic analyses of the thermogenic stage of spadices revealed that the florets, located on the outer parts of the spadix, maintain higher temperatures compared to the inner pith tissue (Onda et al., 2008)." (p5, ls 99-104)

179 and 182 It is not clear what 'this study' and 'that study' refer to.

We have made more specific expressions and clarified which studies are being referred to in the context.

"In the study by Ito-Inaba et al., an initial glimpse into the gene expression landscape in thermogenic and post-thermogenic spadices was provided, yet it failed to fully capture the transcriptomic complexity. This limitation was, in part, due to the inherent restrictions of the SuperSAGE technology. Most crucially, in the study by Ito-Inaba et al., key genes involved in thermogenesis, such as AOX - a central component of plant thermogenic processes, were overlooked." (p8, ls 199-205)

233 Please define what 'small molecule processes' are.

In response to this comment, we have incorporated additional explanations of the 'small molecule metabolic process' based on the definition of the GO term.

"In this study, 'small molecule metabolic process,' as defined in the GO, refers to the small molecules that are characterized by their low molecular weight. These molecules, which include ions, metabolites, and a variety of bioactive

compounds, are involved in a range of cellular processes and functions." (p12, ls 286-289)

237 'Conversely' does not seem appropriate here.

According to the reviewer's comment, we have changed as shown below.

"In contrast, in the pith collected from the thermogenic stage of spadices (Fig 2C), referred to as 'Pith Hot', gene expressions associated with DNA replication and DNA metabolic processes were exclusively increased, suggesting an upregulation in cell division in the pith tissue at this stage." (p12, ls 290-293)

263 Please say what the insights are.

We have added specific explanations as follows.

"For instance, certain clusters, such as clusters 13 and 14, demonstrate a pronounced upregulation of gene expressions associated with the thermogenic stage of florets. Similarly, clusters 2 and 7 highlight unique gene expressions in the pith during the thermogenic stage of spadices, exemplifying potential specialized roles in supporting the thermogenic activity of the florets or in managing stress responses under elevated temperatures in the pith." (p12, ls 306-311)

279-281 It appears to me that Group 2 reveals the largest change between pre-thermogenic and thermogenic stages. However, this group might not receive the attention that it deserves. (It does return later in the paper (325), but maybe it should be flagged earlier.

We have moved the mention of Group F2 earlier in the explanation of the k-means clustering results.

"A particularly notable observation emerged from Group F2, comprising clusters 13 and 14. This group contained genes that exhibited the highest expression levels during the thermogenic stage, in contrast to relatively lower levels at

other stages. Such a distinct expression pattern in Group F2 suggests the involvement of critical gene(s), potentially playing a pivotal role in the thermogenic process.” (p13, ls 317-322)

295-312 What is the functional origin of the titles for these groups? It is difficult for the non-expert to see how they relate to the groups 1-6.

GO terms are assigned based on the known functions of genes. To access the details of GO terms enriched in each group, we have included Supplemental Data S1 and S3.

Reviewer #3 Manuscript Review...

The study by Tanimoto et al. uses a combined transcriptomic and metabolomic approach to investigate potential mechanisms and regulation of thermogenesis in skunk cabbage. Plant thermogenesis is a very interesting and rare phenomenon, limited to a small subset of species. It is, however, of extreme importance to these species, typically in the promotion of pollination by various means, including pollen development and/or the attraction of pollinators. Additionally, the pathway typically associated with plant thermogenesis, alternative respiration, is also vitally important for managing plant stress responses even in non-thermogenic species, therefore broadening the target audience.

The 'omics approach taken here reconfirms old players in skunk cabbage thermogenesis, including the alternative oxidase gene, but importantly also uncovers new potential contributors. Some new candidates included orthologs of known metabolic proteins such as phosphfructokinase and fructose-1,6-bisphosphatase, suggesting a role for futile metabolic cycles in promoting thermogenesis, in addition to electron leakage via alternative respiration. Other candidates included nucleotide metabolism proteins, which could explain altered energy status of the tissue, including a decrease in ATP:AMP during thermogenesis. Sucrose transporter SWEET6 was up-regulated specifically at the thermogenic stage, suggesting that carbohydrate supply may be involved in regulating the initiation of thermogenesis.

One gene candidate in particular, is quite novel and interesting. The authors denoted this gene as SrSBP1/MTO. It was the most highly expressed gene in the thermogenic floret tissue. A protein potentially encoded by this gene was detected by immunoblotting using newly designed antibodies. The authors then purified and partially sequenced this protein from the same tissue to characterise key amino acid residues. They conclude that SBP1/MTO is likely involved in the generation of hydrogen sulfide, which was also shown to accumulate in the thermogenic floret tissue. The authors also show that H2S more effectively inhibits COX compared to AOX, thus high H2S levels may promote alternative respiration within mitochondria purified from floret tissue. Furthermore, at colder temperatures, H2S was more effective at inhibiting COX, therefore favouring AOX and promoting thermogenesis. At warmer temperatures, H2S did not inhibit COX as effectively. This study has therefore uncovered a potential new player in regulation of mitochondrial respiration, which could explain the ability of this plant to not only carry out floral thermogenesis, but thermoregulation in response to fluctuating ambient temperature. This will likely inform future studies on the regulation of alternative respiration, both in thermogenic and non-thermogenic plants.

We would like to express our sincere gratitude to Reviewer #3 for his/her insightful comments and suggestions, which have been instrumental in enhancing the quality of our manuscript.

Below are minor comments/questions relating to specific aspects of the article, in order of appearance.

1. L266 - could a similar analysis be done for pith samples? Do any of the transcript or metabolite changes in the pith confirm a role for this tissue during thermogenesis, e.g. supplying respiratory substrates to the florets?

We have included results from k-means clustering analysis for both florets and pith in Figure 3. Additionally, Figures 4 and Supplemental Figure S4 now present information for both florets and pith. We supplemented the discussion about the role of pith during thermogenesis with the following text:

"Analysis of the pith identified several genes with noteworthy expression patterns. For instance, in Group P1b, sucrose synthase 2 is notable for its expression during the pre-thermogenic stage. The presence of genes encoding heat shock 70kDa proteins 1 and 2 in Group P2 indicates that the increased temperature induces temperature stress in the non-thermogenic pith tissues. Notably, within Group P3, the presence of genes coding for cysteine protease RD21A highlights their potential role in the post-thermogenic pith tissues. For Groups P4 to P6, YTH domain-containing protein ECT2 (Group P4), defensin-like protein P322 (Group P5), and glycine-rich RNA binding protein GRP1A (Group P6) were found (Figure 3E). The list of genes, along with their expression profiles across the thermogenic stages and GO enrichment analyses, is presented in Supplemental Figures S3 and S4. The full dataset of the DEGs categorized by k-means clustering is provided in Supplemental Data S4." (p14, ls 353-365)

2. L380-383 - interesting that alanine levels are high in florets - this could be a source of mitochondrial pyruvate, thus activating AOX (Le et al. 2022, doi: 10.1038/s41477-022-01165-3)

We have mentioned the potential of Ala as a source of mitochondrial pyruvate, potentially leading to the activation of AOX, and included relevant literature.

"Notably, this group also includes Ala, which may act as a precursor for mitochondrial pyruvate, potentially leading to the activation of AOX (Le et al., 2022)." (p17, ls 460-462)

3. L453-457 - clarify that this was experimentally validated at the transcript level, not protein nor activity. PFK1 and F1,6BPase activities are regulated by levels of F2,6BP - are there any data on F2,6BP or the transcript abundance of the enzymes involved in its metabolism, PFK2 / F2,6BPase?

In our study, the experimental data related to the futile cycle were obtained at the transcript level using qRT-PCR. Accordingly, we have revised as shown below.

“Our data indicate a potential involvement of a futile cycle in the florets, comprising ATP-dependent 6-phosphofructokinase (PFK) and fructose-1,6-bisphosphatase (FBPase). Transcript levels for PFK and FBPase were examined using qRT-PCR, which demonstrated their increased co-expression with AOX (Supplemental Figure S7).” (p18, ls 494-498)

Additionally, information about the expression of PFK2 / F2,6BPase genes, although they did not show a significant difference between the florets and pith tissues, has been added to Figure 7A.

4. L616-618 - Zheng et al 2022 also suggested importance of SWEET in sugar transport for thermogenesis.

We have cited literature describing the importance of SWEET in sugar transport for thermogenesis in thermogenic plants.

“For example, sugar transporters, including SWEET, may be involved in the initiation of thermogenesis by enhancing the glycolytic pathway in the florets as previously reported in the carpels of lotus (Zheng et al., 2022).” (p25, ls 686-689)

5. L620-624 - would be good to see an immunoblot to determine if pre-thermogenic transcription of AOX leads to accumulation of the protein at this stage. Also, pyruvate activation has not been mentioned. Could also comment on how the alternative dehydrogenases, e.g. NDA1 from Group 1, might be involved. Looks like an NDB transcript is also up in thermogenic floret (Fig 10).

We have added a discussion about alternative NAD(P)H dehydrogenases as shown below.

“Similarly, the expression levels of genes for alternative dehydrogenases (NDA and NDB) are heightened in the florets during the pre-thermogenic stage of spadices, suggesting their role in enhancing tolerance to the environmental stresses encountered where S. renifolius grows (Sweetman et al., 2019; Jethva et al., 2023).” (p26, ls 696-700)

As for the Western analysis for pre-stage tissues, we could not conduct it due to the small sample size of these tissues.

6. L694-697 - and in animals, see Bremer et al. 2021. doi: 10.1242/jeb.242985

We have incorporated the indicated paper as shown below.

"In plants and invasive marine invertebrates, sulfide treatment has been shown to upregulate AOX and enhance cyanide-tolerant alternative respiratory pathways (Bremer et al., 2021; Fang et al., 2022; Zhong et al., 2023)." (p27, ls 746-748)

7. L690-705 - is it possible that the activity of SBP1/MTO (and therefore H2S concentration) is also temperature-regulated?

While we have not experimentally tested the temperature sensitivity of SBP1/MTO activity, we have discussed this aspect towards the end of the Discussion section.

"Nevertheless, a more extensive investigation is warranted to thoroughly understand the physiological role, including the temperature dependency of its activities, of SBP1/MTO in S. renifolius." (p28, ls 780-782)

8. L726-727 - consider whether increased operation of alternative respiration pathway would also lead to a decrease in ATP levels relative to ADP or AMP.

We have discussed the potential impact of the increased operation of the alternative respiration pathway on the adenylate nucleotide composition.

"Furthermore, the increased capacities of the mitochondrial alternative respiration pathway in the florets of thermogenic spadices (Onda et al., 2007), also contribute to the decreased ATP levels, which in turn lead to an increased level of AMP." (p29, ls 794-797)

9. L935-945 - why was mannitol used as osmoticum in mitochondrial grinding

and wash media, but sucrose in the percoll and assay solutions? Were mitochondria still reasonably intact after freeze-thawing for respiratory activity measurements?

After various preliminary experiments, we have adopted the most effective buffer composition for each stage of mitochondrial purification and respiration measurement. Our experimental method for mitochondrial isolation in the present study follows the same fundamental procedure as our previous studies (e.g., Onda et al., 2007; Umekawa et al., 2016). The integrity of mitochondria may be partially compromised due to freeze-thawing. However, in our study, we used an experimental system where artificial substrates are directly supplies to the terminal oxidases (AOX & COX), as presented in the Supplemental Figure S13. Therefore, even if freeze-thawing caused damage, it would not be a problem for determining oxygen consumption in our experiments. Moreover, cytochrome c, which may leak out due to the freeze-thawing, it is experimentally supplemented during our assay.

10. Fig 2A-C - are DEGs calculated as florets relative to pith? It seems so, based on methods, but should explicitly state this in legend.

The fold change (FC) was calculated based on Florets/Pith comparison, and they are clearly stated in the Materials and Methods section and relevant figure captions.

11. Figs 3A and 7 - Pre_P1 missing from metabolomics clustering analysis (Fig 7) but Pre_P4 missing from DEG clustering analysis (Fig 3A). Why were different replicates excluded?

We have described in the caption of Figure 1 as shown below.

"Empty boxes in 'Pre_1' and 'Pre_4' denote samples that were not subjected to CE-MS or RNA-seq analysis due to insufficient tissue quantity resulting from the small size of the pith-tissues at the pre-thermogenic spadices."

12. Figure 10 - clarify which data were used - is it only from thermogenic stage as stated in L447-448? Because the legend states it is across different

thermogenesis stages - if so, were the data from different thermogenic stages averaged together?

Figures 7, Supplemental Figures S8, S14, and S15 clearly show that comparison was made between Hot Florets and Hot Pith during the thermogenic stage. The figures display the average values of four biological replications for both Hot Florets and Hot Pith.

13. Figure 11 C,D - when measured in the presence of inhibitors, AOX and COX should be reported as "capacities" rather than "activities". Same at L945-950.

We have changed the terms 'AOX activities' and 'COX activities' to 'capacities.' Thank you.

14. Figure S1 - why are some conditions not reported, e.g. Group 6 Hot? Also check order of graphs from Groups 5 and 6.

Supplemental Figure S3 now presents the top 10 genes in both the florets and pith tissues across the pre-, hot, and post-thermogenic stages of the spadices. Additionally, the order of the graphs is correctly arranged.

18-Dec-2023

Prof. Kikukatsu Ito
Faculty of Agriculture, Iwate University
Morioka, Iwate 020-8550
Japan

RE: A Convergence of Gene and Metabolite Expression in the Thermogenic Spadix of Skunk Cabbage, *Symplocarpus renifolius*

Dear Kikukatsu,

We have now received the final review and I am delighted to share good news: we are pleased to accept your manuscript for publication in the Plant Physiology. This acceptance is contingent on revision based on the comments of our reviewers; those are all very minor points that will be starightforward for you to implement. In particular, reviewer #1 raises several useful points that deserve clarification / correction; also reviewer #3 raises a question that will be important to be clarified in the published paper and will be quick and straightforward to consider.

Please highlight all changes and include a detailed annotation to changes to the text, with line numbers, and noting your responses to the comments.

To submit your revised manuscript, click:

Link Not Available

If you cannot return the revised manuscript within 8 weeks of receipt, please let us know. Otherwise, we will assume that you have elected not to revise the manuscript and are withdrawing it.

Thank you for allowing us to review your work. We look forward to hearing from you soon.

Sincerely,
Plant Physiology Board of Editors

--- Reviewer comments:
Reviewer #1 (Comments for the Author):

The authors of the manuscript titled "A Convergence of Gene and Metabolite Expression in the Thermogenic Spadix of Skunk Cabbage, Symplocarpus renifolius," incorporated the majority of suggestions provided by the reviewers and addressed the main points raised in the revised version. I am content with this updated version and anticipate that the scientific community will benefit from a valuable study on the regulation of thermogenesis. Nevertheless, I would appreciate further clarification on certain aspects that remain unclear. Additionally, I have some suggestions that the authors may choose to incorporate at their discretion.

Points not clear: Are piths thermogenic or not? It appears to me that piths exhibit elevated temperatures, although not as high as in florets. I don't believe there is an issue with the way the authors conducted the analysis and comparisons; however, clarity is lacking in some sections of the text and the response letter. I will provide specific examples below:

In lines 99-104, it is stated, "The spadix of S. renifolius is composed of florets and pith. Our previous thermoscopic analyses of the thermogenic stage of spadices revealed that the florets, located on the outer parts of the spadix, maintain higher temperatures compared to the inner pith tissue (Onda et al., 2008). Thus, it has been suggested that thermogenesis predominantly occurs in the florets, while the pith does not directly contribute to this process in the spadix (Onda et al., 2008)." In the study available at https://doi.org/10.1104/pp.107.113563, the analyzed spadix was at the stigma stage and had a temperature of 23.4{degree sign}C before cutting when the ambient temperature was 8.5{degree sign}C. The spadix was cut to generate a longitudinal section (Fig. 2, C and F; Supplemental Movie S1), and the surface temperatures of this longitudinal section revealed that the florets surrounding the spadix displayed a higher temperature than the rest of the spadix (Fig. 2, D and G; Supplemental Movies S2 and S3). Even at 2 days after the preparation of the longitudinal sections, the temperature of the florets was found to be 20.7{degree sign}C {plus minus} 0.1{degree sign}C (n = 3), whereas the temperature at the center of the

spadix was measured as 19.5{degree sign}C {plus minus} 0.2{degree sign}C (Supplemental Movie S4). I agree with the authors that florets display higher temperatures than pith; however, I am not entirely convinced that piths are entirely non-thermogenic. In the case of the previously published paper, there was only a one-degree difference between the temperatures of florets and piths. Could the minimal temperature difference between piths and florets be attributed to heat transfer from florets to piths?

The authors responded that this comparative approach aims to highlight the differential thermogenic activity and molecular profiles between actively thermogenic florets and the less thermogenic pith. Does this imply that piths are thermogenic but to a lesser degree? Moreover, it is unclear when the statement is made that piths are not thermogenic, while data on pre, hot, and post-hot stages are presented for piths. Did this mean that pre-hot piths were collected with pre-hot florets, and so on? If so, please clarify this in the text and the Materials and Methods section. The current wording suggests that piths were collected based on their temperatures. Additionally, regarding the comparisons made in the transcriptome and metabolome, when florets in each state are compared to piths, what are the piths in different states being compared to? Clarification on this point is needed.

Line 199: In the study by Ito-Inaba et al., mention the year

Line 256: To compare gene expression profiles in thermogenic florets with those in non-thermogenic pith. Author answer to me: This comparative approach allows us to highlight the differential thermogenic activity and molecular profiles between the florets, which are actively thermogenic, and the pith, which exhibits less thermogenic activity. I think authors should remove statements like pith is not thermogenic or explain better why the temperatures in pith are also high. It is not clear, to me it seems piths are thermogenic but in a lower degree than florets.

Lines 256 - 261: To compare gene expression profiles in thermogenic florets with those in non-thermogenic pith, we identified differentially expressed genes (DEGs) for each thermogenic stage (Figure 2A, Supplemental Figure S2A and Supplemental Data S2). This analysis revealed a total of 3,624 DEGs, among these 1,302 in the florets and 2,322 in the pith (Supplemental Figure S2B and Supplemental Data S3). Here one thing was not clear to me: were the comparisons of florets-pith made in the same stage? For example, a hot floret was compared to the pith from the same spadix? Please clarify in the text.

272-276: Similarly, within cluster B, pre-thermogenic, thermogenic, and post-thermogenic pith samples were categorized into subclusters Bba, Bbb, and Ba, respectively. These findings suggested that the samples we collected accurately reflected the tissue- and developmental stage-specific aspects of thermogenesis. If authors say piths are not thermogenic, how did they cathegorized piths in pre, hot and post? If those terms mean only piths collected with the respective florets in the same spadix at the same stage please clarify. If not, state in the text that piths are also thermogenic but in a lower degree.

Line 85: put comma after '

Figure 5D - Please check if it is really sugar phosphates

Lines 445-451: revise metabolites changed, the writing does not fit with the figure. Furthermore, how is the regulation of AOX activity in this species? There was an increase in succinate that was not mentioned and that could play a role on that.

470 - 472: Our results revealed that only the AMP level in thermogenic florets significantly increased over the three thermogenic stages, and between the florets and the pith. ADP also changed from pre to hot.

560-566: Moreover, from western blot analysis of two distinct epitopes against SBP1/MTO, we detected specific expression in the thermogenic florets, compared to the non-thermogenic pith, leaf, and spathe (Figure 8B). I disagree that the expression was specific for thermogenic florets because you also see a bit of expression in piths, it just more expressed in florets than in piths. Furthermore I was curious to know the levels of H2S between hot/non hot florets and hot/non hot piths. It is fine if authors do not have that, I just wondered if there is an induction of H2S production during thermogenesis when comparing with the same tissue but not thermogenic.

794-797: Furthermore, the increased capacities of the mitochondrial alternative respiration pathway in the florets of thermogenic spadices (Onda et al., 2007), also contribute to the decreased ATP levels, which in turn lead to an increased level of AMP. You just observed a trend of ATP reduction.

Suggestion: Show Figure 2B; Figure 3D-E; Figure 6 as mean (averaged). It will be easier for visualization. Authors could show the individual samples in the supplemental data. Furthermore, there would be more space to increase the size of the panels making it easier to read the name of genes/metabolites. I also suggest a creation of a PCA in figure 1 for transcriptome and it was done for metabolome to show a clear separation between stages/tissues.

Reviewer #2 (Comments for the Author):

Congratulations for your hard work and new discoveries.

Reviewer #3 (Comments for the Author):

I am generally satisfied with the alterations made to the ms. My own previous comments were adequately addressed, and it appears the other reviewers' comments also received the attention they deserved.

I am undecided about whether down-regulated DEGs in florets should be defined as up-regulated DEGs in pith (e.g. Fig 2 volcano plots). If looking for DEGs in pith in response to floral thermogenesis, then shouldn't comparisons be made between timepoints, (e.g. hot pith c.f. pre-thermogenic pith), rather than between tissues at the same thermogenic stage? Nevertheless, this should not prevent publication - it is a robust and interesting paper.

Manuscript ID: PP2023-RA-01320R2
Title: A Convergence of Gene and Metabolite Expression in the Thermogenic Spadix of Skunk Cabbage, *Symplocarpus renifolius*
Authors: Haruka Tanimoto, Yui Umekawa, Hideyuki Takahashi, Kota Goto, and Kikukatsu Ito

The revised MS has the revisions indicated in red type.
Responses from the authors (in blue italic type).

Reviewer comments:

Reviewer #1 (Comments for the Author):

The authors of the manuscript titled "A Convergence of Gene and Metabolite Expression in the Thermogenic Spadix of Skunk Cabbage, Symplocarpus renifolius," incorporated the majority of suggestions provided by the reviewers and addressed the main points raised in the revised version. I am content with this updated version and anticipate that the scientific community will benefit from a valuable study on the regulation of thermogenesis.

Nevertheless, I would appreciate further clarification on certain aspects that remain unclear. Additionally, I have some suggestions that the authors may choose to incorporate at their discretion.

We deeply appreciate your constructive comments and the opportunity to further clarify and enhance our manuscript. Your insights have been invaluable in refining our study. Below, we address each of your points in detail.

Points not clear: Are piths thermogenic or not? It appears to me that piths exhibit elevated temperatures, although not as high as in florets. I don't believe there is an issue with the way the authors conducted the analysis and comparisons; however, clarity is lacking in some sections of the text and the response letter.
I will provide specific examples below:

In lines 99-104, it is stated, "The spadix of S. renifolius is composed of florets

and pith. Our previous thermoscopic analyses of the thermogenic stage of spadices revealed that the florets, located on the outer parts of the spadix, maintain higher temperatures compared to the inner pith tissue (Onda et al., 2008). Thus, it has been suggested that thermogenesis predominantly occurs in the florets, while the pith does not directly contribute to this process in the spadix (Onda et al., 2008)." In the study available at https://doi.org/10.1104/pp.107.113563, the analyzed spadix was at the stigma stage and had a temperature of 23.4{degree sign}C before cutting when the ambient temperature was 8.5{degree sign}C. The spadix was cut to generate a longitudinal section (Fig. 2, C and F; Supplemental Movie S1), and the surface temperatures of this longitudinal section revealed that the florets surrounding the spadix displayed a higher temperature than the rest of the spadix (Fig. 2, D and G; Supplemental Movies S2 and S3). Even at 2 days after the preparation of the longitudinal sections, the temperature of the florets was found to be 20.7{degree sign}C {plus minus} 0.1{degree sign}C (n = 3), whereas the temperature at the center of the spadix was measured as 19.5{degree sign}C {plus minus} 0.2{degree sign}C (Supplemental Movie S4). I agree with the authors that florets display higher temperatures than pith; however, I am not entirely convinced that piths are entirely non-thermogenic. In the case of the previously published paper, there was only a one-degree difference between the temperatures of florets and piths. Could the minimal temperature difference between piths and florets be attributed to heat transfer from florets to piths?

We are grateful for your additional comments regarding the thermogenic role of piths in the spadix. As you rightly pointed out, our previous findings (Onda et al., 2008) do indicate that the thermogenic activity in the pith tissue is notably less pronounced compared to that of the florets during the thermogenic stage. The observed temperature variation between the florets and the pith tissues may indeed be attributed to the differing extents of thermogenic activities of each tissue, as well as the possible heat transfer from the more thermogenically active florets to the piths.

Based on our previous thermoscopic analysis (Onda et al., 2008), the florets, positioned in the outer part of the spadix, are the primary sites of thermogenesis. The inner pith tissue, exhibiting lower temperatures, is thus characterized as having less thermogenic capacity compared to the florets. This

differential thermogenic role is crucial for a comprehensive understanding of the thermogenesis in the spadix and is now clearly conveyed in our revised manuscript. Specifically, to address your concerns and enhance clarity, we have revised the first part of the results section as follows:

"In our previous study, we demonstrated that the florets, located in the outer part of the spadix, were the primary tissues for thermogenesis (Onda et al., 2008). Therefore, in the present study, we assume that the inner pith is the less thermogenic tissue in the spadix." (lines 225-229)

The authors responded that this comparative approach aims to highlight the differential thermogenic activity and molecular profiles between actively thermogenic florets and the less thermogenic pith. Does this imply that piths are thermogenic but to a lesser degree?

Yes, as stated above, we have clearly indicated that in the present study, we hypothesize that the inner pith exhibits lesser thermogenic activity compared to the florets in the spadix.

Moreover, it is unclear when the statement is made that piths are not thermogenic, while data on pre, hot, and post-hot stages are presented for piths. Did this mean that pre-hot piths were collected with pre-hot florets, and so on? If so, please clarify this in the text and the Materials and Methods section. The current wording suggests that piths were collected based on their temperatures.

Based on the reviewer's comments, we have revised the text, as exemplified below:

"in the pith collected from the thermogenic stage of spadices (Figure 2C), referred to as 'Pith Hot'." (lines 309-310)

"To explore the temporal and spatial dynamics of metabolic shifts in the florets and pith tissues from pre- to post-thermogenic stages of spadices, we first conducted a PCA." (lines 428-430)

“Specifically, both the florets and pith samples from pre-thermogenic stage of spadices were located at the top of the panel, demonstrating a degree of overlap.” (lines 435-437)

“Cluster A consisted of the pith tissue samples from spadices at pre-thermogenic, thermogenic, and post-thermogenic stages, along with samples from the florets of pre- and post-thermogenic spadices. In contrast, Cluster B encompassed four samples from the florets of a thermogenic spadix (Hot_F1-F4) and one sample from the pith of pre-thermogenic florets (Pre_F2). This cluster could be further divided into three sub-clusters: Baa, Bab, and Bb. Sub-cluster Baa contained a florets sample from a pre-thermogenic spadix (Pre_F2), while Bab and Bb included the four florets samples from thermogenic spadices (Hot_F1-F4).” (lines 445-453)

“It is also worth noting that the gene expression profile of Pre_F2 fell into the same cluster as other florets samples from pre-thermogenic spadices (Figure 2B and Supplemental Figure S3A).” (lines 461-464)

“Each sample is identified by its thermogenic stage (Pre, Hot, or Post) and tissue type (F for florets, P for pith), as represented by respective identifiers (e.g., Hot_P4 refers to the fourth pith sample collected from the thermogenic stage of spadices).” (lines 1590-1593)

“Sample labels are combinations of tissue type (florets or pith) and thermogenic stage (pre-thermogenic (Pre), thermogenic (Hot), and post-thermogenic (Post) spadices), e.g., 'Pre_Florets' indicates the floret samples from the pre-thermogenic stage of spadices.” (lines 1811-1814)

“'Pre Florets', 'Hot Florets', and 'Post Florets' refer to florets collected from spadices at pre-thermogenic, thermogenic, and post-thermogenic stages, respectively. Similarly, 'Pre Pith', 'Hot Pith', and 'Post Pith' denote pith tissues collected from spadices during the corresponding stages of thermogenesis.” (lines 1886-1890)

“In both panels (A) and (B), the data analyzed were divided into six differently colored groups to represent different stages and parts of the spadix i.e. florets

from pre-thermogenic spadices (Pre Florets), florets from thermogenic spadices (Hot Florets), florets from post-thermogenic spadices (Post Florets), pith from pre-thermogenic spadices (Pre Pith), pith from thermogenic spadices (Hot Pith), and pith from post-thermogenic spadices (Post Pith)." (lines 1970-1976)

Additionally, regarding the comparisons made in the transcriptome and metabolome, when florets in each state are compared to piths, what are the piths in different states being compared to? Clarification on this point is needed.

In the Results section, under the subtitle 'Integrated Transcriptome and Metabolome Analyses at the Thermogenic Stage of the Spadix,' we have clearly stated the following to clarify our comparison approach:

"We next focused on a comparative analysis of gene expressions and metabolite accumulations in the florets and the pith in the same thermogenic spadix. This in-depth comparison uncovered specific changes associated with the molecular processes in the florets of the thermogenic spadix." (lines 521-524)

Line 199: In the study by Ito-Inaba et al., mention the year

We have updated the text to include the year of the Ito-Inaba et al. study, accordingly. (lines 197 & 201)

Line 256: To compare gene expression profiles in thermogenic florets with those in non-thermogenic pith. Author answer to me: This comparative approach allows us to highlight the differential thermogenic activity and molecular profiles between the florets, which are actively thermogenic, and the pith, which exhibits less thermogenic activity. I think authors should remove statements like pith is not thermogenic or explain better why the temperatures in pith are also high. It is not clear, to me it seems piths are thermogenic but in a lower degree than florets.

We have revised the term 'non-thermogenic pith' to 'less thermogenic pith' in the manuscript. The revised text now reads:

“To compare gene expression profiles in thermogenic florets with those in less thermogenic pith, we identified differentially expressed genes (DEGs) for each thermogenic stage (Figure 2A, Supplemental Figure S2A and Supplemental Data S2).” (lines 254-257)

“Similarly, within cluster B, less thermogenic pith samples from the Pre, Hot, and Post stages of spadices were categorized into subclusters Bbb, Bba, and Ba, respectively.” (lines 273-276)

Lines 256 - 261: To compare gene expression profiles in thermogenic florets with those in non-thermogenic pith, we identified differentially expressed genes (DEGs) for each thermogenic stage (Figure 2A, Supplemental Figure S2A and Supplemental Data S2). This analysis revealed a total of 3,624 DEGs, among these 1,302 in the florets and 2,322 in the pith (Supplemental Figure S2B and Supplemental Data S3). Here one thing was not clear to me: were the comparisons of florets-pith made in the same stage? For example, a hot floret was compared to the pith from the same spadix? Please clarify in the text.

According to the reviewer’s comments, we have revised the text as follows:

“To compare gene expression profiles in thermogenic florets with those in less thermogenic pith, we identified differentially expressed genes (DEGs) for each thermogenic stage (Figure 2A, Supplemental Figure S2A and Supplemental Data S2). We first created a pooled set (5,081 genes) by combining genes from both the florets and the pith at various thermogenic stages. Redundant genes in this pool, found either in the florets or the pith, were subsequently removed, resulting in the exclusion of 1,457 genes. This analysis revealed a total of 3,624 DEGs, with 1,302 in the florets and 2,322 in the pith (Supplemental Figure S2B and Supplemental Data S3).” (lines 254-262)

272-276: Similarly, within cluster B, pre-thermogenic, thermogenic, and post-thermogenic pith samples were categorized into subclusters Bba, Bbb, and Ba, respectively. These findings suggested that the samples we collected accurately reflected the tissue- and developmental stage-specific aspects of thermogenesis. If authors say piths are not thermogenic, how did they cathegorized piths in pre, hot and post? If those terms mean only piths collected

with the respective florets in the same spadix at the same stage please clarify. If not, state in the text that piths are also thermogenic but in a lower degree.

According to the reviewer's comment, we have explicitly stated that the pith tissue is less thermogenic, as shown in the following texts:

"To compare gene expression profiles in thermogenic florets with those in less thermogenic pith, we identified differentially expressed genes (DEGs) for each thermogenic stage (Figure 2A, Supplemental Figure S2A and Supplemental Data S2)." (lines 254-257)

"Similarly, within cluster B, less thermogenic pith samples from the Pre, Hot, and Post stages of spadices were categorized into subclusters Bbb, Bba, and Ba, respectively." (lines 273-276)

"While both florets and pith undergo thermal changes during the development of the spadix, it is essential to note here that the pith exhibits a less intense thermogenic activity compared to the florets." (lines 276-278)

Line 85: put comma after '

Revised accordingly.

Figure 5D - Please check if it is really sugar phosphates

We have replaced 'sugar phosphates' with 'sugars and sugar phosphates' accordingly. Thank you.

Lines 445-451: revise metabolites changed, the writing does not fit with the figure.

The reviewer rightly pointed out the absence of CDP in the text, which we have now been updated accordingly to reflect this addition. We thank the reviewer for highlighting this oversight.

"Of the metabolites examined, 2-oxoglutarate, GTP, CDP, Cys, 4-hydroxyproline, and glucuronate were markedly upregulated in the florets, displaying a more than two-fold increase in accumulation compared to the pith (Figure 5D)." (lines 478-481)

Furthermore, how is the regulation of AOX activity in this species? There was an increase in succinate that was not mentioned and that could play a role on that.

In S. renifolius, AOX is activated by pyruvate, not succinate (Onda et al., FEBS Lett., 2007, doi.org/10.1016/j.febslet.2007.11.061), and the level of pyruvate did not show any significant changes in our analysis as shown in Fig. 5D.

470 - 472: Our results revealed that only the AMP level in thermogenic florets significantly increased over the three thermogenic stages, and between the florets and the pith. ADP also changed from pre to hot.

As stated in our statistical analysis presented in Fig. 6A, it is evident that the AMP level in the florets of the thermogenic stage of spadices significantly increased compared to the florets and pith samples from pre-, thermogenic, and post-thermogenic stages. Although the ADP level in the florets increased from the pre- to the thermogenic stage, there was no statistical significance between the thermogenic and post-thermogenic stages. Therefore, we concluded that the increased levels of AMP in the florets of thermogenic spadices play a specific role in developmentally and tissue-specific thermogenesis in this plant.

560-566: Moreover, from western blot analysis of two distinct epitopes against SBP1/MTO, we detected specific expression in the thermogenic florets, compared to the non-thermogenic pith, leaf, and spathe (Figure 8B). I disagree that the expression was specific for thermogenic florets because you also see a bit of expression in piths, it just more expressed in florets than in piths.

Following the reviewer's comment, we have revised the text as follows:

"In our western blot analysis, which targeted two distinct epitopes of SBP1/MTO in various samples from the thermogenic stage of S. renifolius, we observed a

pronounced expression of SBP1/MTO proteins in the florets with both antibodies (Figure 8B). Additionally, we detected a faint but detectable expression in the pith with the antibodies against the N-terminal epitope." (lines 593-597)

Furthermore I was curious to know the levels of H2S between hot/non hot florets and hot/non hot piths. It is fine if authors do not have that, I just wondered if there is an induction of H2S production during thermogenesis when comparing with the same tissue but not thermogenic.

In the present study, we did not conduct experiments to measure H_2S levels between hot and non-hot florets or piths. However, we recognize the significance of this issue. It is certainly an important aspect that we aim to explore in future research.

794-797: Furthermore, the increased capacities of the mitochondrial alternative respiration pathway in the florets of thermogenic spadices (Onda et al., 2007), also contribute to the decreased ATP levels, which in turn lead to an increased level of AMP. You just observed a trend of ATP reduction.

We have modified the text in response to the reviewer's comment, as shown below:

"Furthermore, the increased capacities of the mitochondrial alternative respiration pathway in the florets of thermogenic spadices, as previously reported by Onda et al., (2007) could decrease the ATP/ADP ratio; this change may, in turn, result in an increased level of AMP through the upregulated gene expression of adenylate kinase (Figure 6B)." (lines 830-835)

Suggestion: Show Figure 2B; Figure 3D-E; Figure 6 as mean (averaged). It will be easier for visualization. Authors could show the individual samples in the supplemental data. Furthermore, there would be more space to increase the size of the panels making it easier to read the name of genes/metabolites. I also suggest a creation of a PCA in figure 1 for transcriptome and it was done for metabolome to show a clear separation between stages/tissues.

We respectfully disagree with the suggestion to display average values in the heatmap of Figure 2B. Displaying individual sample data is crucial, as it allows for the discernment of variations among samples with different tissue types and thermogenic stages. Consequently, we have opted to maintain the current representation in Figure 2B.

Figures 3D-E have been updated to display z-score mean values, and individual TMP values are now included in Supplemental Data S4. Although Figure 6 already presents mean values, we have compiled individual data into a new Excel file, designated as Supplemental Data S6.

Additionally, we have performed a new PCA on the transcriptome data. The results of this analysis are now included as Supplemental Figures S3 and S6.

The descriptive texts for Supplemental Figures S3 and S6 are as follows:

"To further clarify the relationship between the gene expression profile and the thermogenic stages of the spadix, we performed a principal component analysis (PCA) on DEGs (Supplemental Figure S3A). In our PCA analysis, all samples collected in the present study were mapped into six major clusters, which primarily differentiated them in a tissue- and thermogenic stage-specific manner. Namely, PC1 accounted for 37.1% of the variance, corresponding to the differences between the florets and pith, while PC2 contributed to 23.2% of the separation, associated with transitions between different thermogenic stages (Supplemental Figure S3A). DEGs specific to either florets or pith clearly possess significant contributions to the tissue-specific gene expressions, as accounted for by PC1 loadings (Supplemental Figure S3B and Supplemental Data S4). However, it is notable that these tissue-specific classifications do not fully explain the variance for thermogenic stage-specific gene expression, as indicated by PC2 loadings (Supplemental Figure S3B)." (Supplemental Figures S3; lines 282-295)

"We further investigated whether the individual groups, as classified above, contributed to the PCA variance related to tissue- and thermogenic stage-specific gene expressions, as depicted in Supplemental Figure S3. Our results confirmed that each group associated with the florets (Groups F1-F6) and pith

(Groups P1a & 1b - P6) substantially contributed to the tissue-specific gene expression, as indicated by PC1 loadings (Supplemental Figure S6A). In contrast, DEGs in Groups F1-F4 and Groups P1a, P3, P5, P6 appeared to contribute more significantly to the thermogenic stage-specific gene expression, as indicated by PC2 loadings (Supplemental Figure S6B). Detailed data for this analysis can be found in Supplemental Data S6." (Supplemental Figures S6; lines 386-395)

--

Reviewer #2 (Comments for the Author):

Congratulations for your hard work and new discoveries.

--

Reviewer #3 (Comments for the Author):

I am generally satisfied with the alterations made to the ms. My own previous comments were adequately addressed, and it appears the other reviewers' comments also received the attention they deserved.

I am undecided about whether down-regulated DEGs in florets should be defined as up-regulated DEGs in pith (e.g. Fig 2 volcano plots). If looking for DEGs in pith in response to floral thermogenesis, then shouldn't comparisons be made between timepoints, (e.g. hot pith c.f. pre-thermogenic pith), rather than between tissues at the same thermogenic stage? Nevertheless, this should not prevent publication - it is a robust and interesting paper.

We are grateful for your insightful feedback regarding the interpretation of DEGs in our study.

Indeed, the DEGs that are down-regulated in the florets correspond to those up-regulated in the pith during the same developmental stage of the spadix. This relationship is explained in the revised text (lines 254-262) and further detailed in Supplementary Figure S2A. In the present study, we first compared the gene

expressions between the florets and pith at identical thermogenic stages (pre-thermogenic, thermogenic, or post-thermogenic) of the spadix. We then selected genes specifically expressed in either the florets or pith at each stage. After combining these selected genes and removing redundant ones, we created a final gene pool. This pool, identified as the DEGs for our study, forms the basis for further analyses.

In our research, as depicted in Figure 3, we specifically employed non-hierarchical k-means clustering to facilitate comparisons across different timepoints of the identified DEGs, reflecting the developmental progression of the spadix. This methodological approach enables us to analyze gene expression changes not only between florets and pith during the same thermogenic stage but also across the various developmental stages of the spadix. We believe that this approach significantly strengthens our findings, offering a multi-dimensional perspective on the thermogenic process in the spadix of S. renifolius.

11-Jan-2024
Prof. Kikukatsu Ito
Faculty of Agriculture, Iwate University
Morioka, Iwate 020-8550
Japan

MSID: PP2023-RA-01320R2
MS TITLE: A Convergence of Gene and Metabolite Expression in the Thermogenic Spadix of Skunk Cabbage, Symplocarpus renifolius

Dear Dr. Kikukatsu Ito:

Thank you for the resubmission of your carefully revised manuscript. I am pleased to inform you that your revised manuscript "A Convergence of Gene and Metabolite Expression in the Thermogenic Spadix of Skunk Cabbage, Symplocarpus renifolius" has been accepted for publication in Plant Physiology. I would like to relay my congratulations on this excellent piece of work.

Your manuscript will be checked for consistency with journal formatting and image standards by one of our Science Editors, who will contact you shortly. They may have a number of queries and suggestions for you. Please keep in mind that they work to increase the online visibility of your article and its attraction for readers from the widest of backgrounds. You will have the opportunity to review any revisions, and I hope you will work constructively with them to get the most out of your research. You will also have the opportunity to review all changes when you receive the author proofs for your article.

Once validated for publication, your article will be sent to a Science Editor for a final review of your manuscript and source files. After your final files are uploaded, your article will appear in the next available issue and the online Preview version of your article will be posted after your signed Copyright and Checklist forms and final source files are received.

Please be sure to include the following in your manuscript file:
Harvard style citations
List of all author contributions and funding information at the beginning of manuscript after the titles and author list.
List all supplemental materials associated with the article, with titles, at the end of the ms, before the references and acknowledgments section.
Complete Figure Legends for all figures appearing in the article listed before the Literature Cited.

Thank you and congratulations on your Plant Physiology paper!

Sincerely,
Plant Physiology Board of Editors

=====
FIRST AUTHOR PROFILES
=====

We recognize the hard work that goes into being First Author of a Plant Physiology paper by publishing a "First Author Feature" for each First Author alongside your paper.

If the first author(s) would like to submit information for a feature on Facebook and Twitter, fill out the form by clicking on the link below:

Plant Physiology First Author Feature Form: https://forms.office.com/r/ZBCGZfhm8F
If you cannot access the form through the link above, please contact Rachel at rbelsky@aspb.org.

To upload an image of yourself to accompany your profile, please put your photo in the correct journal folder at the link below. You must label your image: Author First Name Last Name Article Title.

First Author Profile Image Dropbox: https://www.dropbox.com/scl/fo/xz029p7qjr2jml2rp2kmq/h?dl=0&rlkey=6fkxcaqmxx35ap152b2osy1fz

=====
IMPORTANT REMINDER: PEER REVIEW REPORTS
=====

If you opted to publish a peer review report along with your article during the original submission process, it will be prepared by the editorial staff and publicly posted with your manuscript, inside the zip file that contains any other supplemental material. As a reminder, the peer review report is a public record of all comments from editors and reviewers, as well as your prior responses, as you received them in the decision letters for each draft of your manuscript. If you agreed to publish this report and have changed your mind, or are not sure if you selected this option, please contact the editorial office as soon as possible before signing the license agreement from our publisher.
